# Primary intracranial myxopapillary ependymoma: two case reports and literature review

**DOI:** 10.3389/fonc.2026.1763325

**Published:** 2026-05-21

**Authors:** Lan Zhong, Zhen Xu, Shiyuan Yin, Hongjuan Tian, Feizhou Du, Chuande Zhang, Yihua Chen, Chao Wang

**Affiliations:** 1 Department of Pathology, The General Hospital of Western Theater Command, Chengdu, China; 2 Department of Radiology, The General Hospital of Western Theater Command, Chengdu, China

**Keywords:** case reports, cerebral parenchymal, frontal lobe, myxopapillary ependymoma, neuro-oncology, pathology

## Abstract

Myxopapillary ependymoma (MPE), a histological variant of ependymoma, is characterized by a radial arrangement of spindled or epithelioid tumor cells surrounding blood vessels, accompanied by perivascular myxoid changes and microcyst formation. MPE typically occurs in young adults, predominantly in the conus medullaris, cauda equina, and filum terminale. However, primary intracranial MPEs, especially those arising in the cerebral parenchyma, are exceptionally rare. Here, we present two cases of isolated MPEs located in the frontal lobe to underscore their extraordinary rarity. We provide a comprehensive analysis of their clinical characteristics, imaging findings, and pathological features to deepen the understanding and recognition of this rare entity.

## 1 Introduction

MPE is a glial neoplasm that represents a slow-growing variant of ependymoma (1). According to the 2021 WHO classification of central nervous system (CNS) tumors, MPE is classified as grade 2 (2).

Due to its rarity in the intracranial compartment, there are no established guidelines for diagnosing and treating primary intracranial MPE. Thus, the management strategies are primarily extrapolated from experiences with spinal MPEs. Primary intracranial MPEs can present with a range of symptoms depending on their location. Pathological examination is the primary method for diagnosing intracranial MPE. The preferred treatment for MPE is surgical resection. Complete tumor removal, followed by adjuvant radiotherapy, can significantly improve patient prognosis (3, 4).

## 2 Case presentation

### 2.1 Case 1

**Figure 1:**
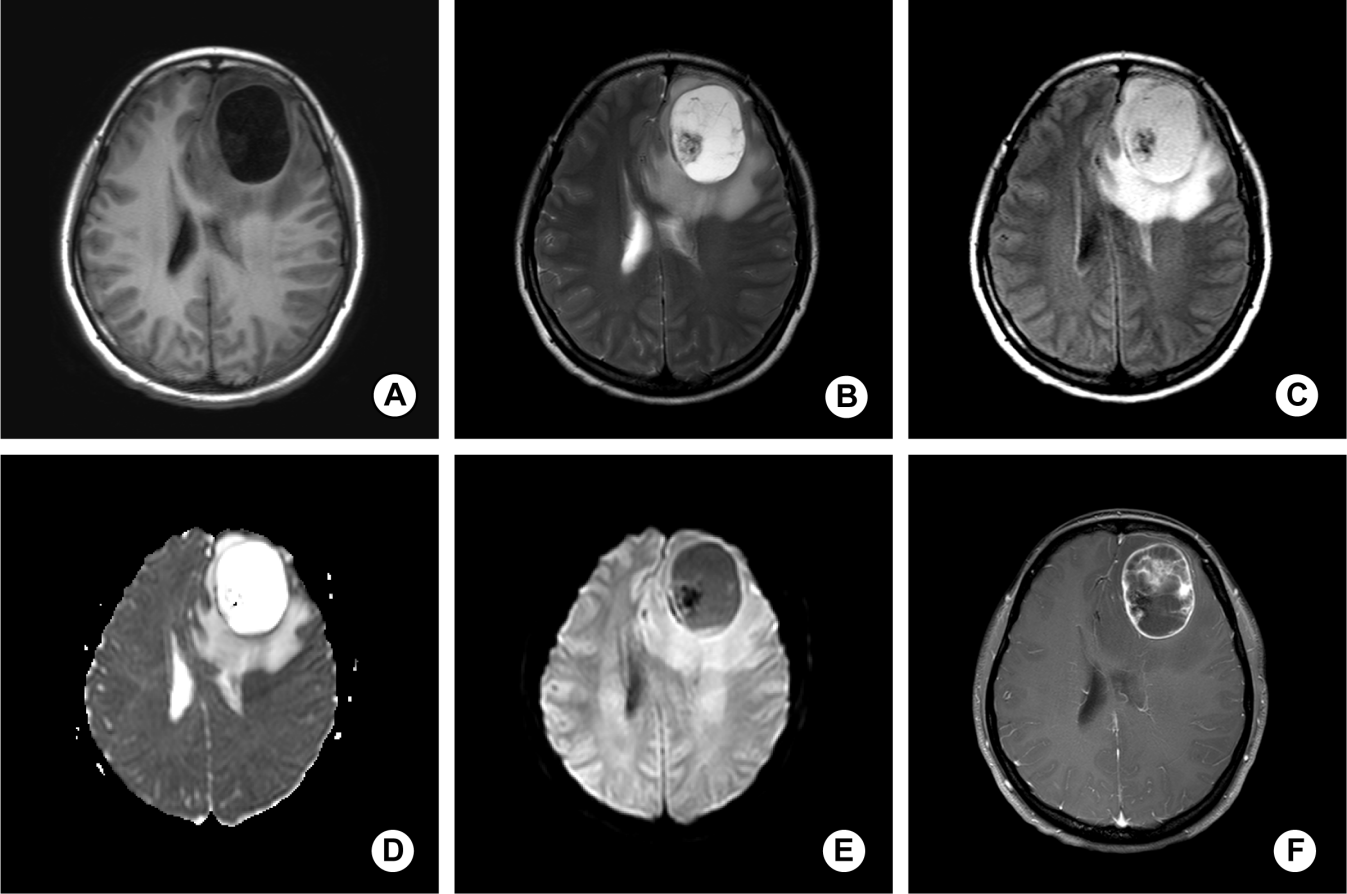
**(A)** MRI showed the tumor located in the frontal lobe was a well-circumscribed mass, and the lesion was predominantly hypointense with an isointense solid nodule on T1-weighted images. **(B)** The tumor appeared predominantly hyperintense with the solid component showing iso- to hypointense signals on T2-weighted images. **(C)** The tumor was hyperintense on FLAIR sequences. **(D)** The tumor showed no diffusion restriction on DWI. **(E, F)** Post-contrast T1-weighted fat-suppressed images showed peripheral rim enhancement and patchy enhancement of solid components.

A 31-year-old woman presented with an acute onset of pulsatile cephalalgia within 72 hours following vigorous aerobic exercise (distance running), with the pain intensity fluctuating between attacks. Brain magnetic resonance imaging (MRI) revealed a cystic-solid mass in the left frontal lobe, measuring approximately 4.8 × 3.6 cm. The lesion exhibited well-defined margins, with compression of the adjacent anterior horn of the left lateral ventricle and a mild rightward shift of the midline structures. On T1-weighted images, the lesion was predominantly hypointense with an isointense solid nodule (shown in **Figure 1A**). On T2-weighted images, it appeared predominantly hyperintense, with the solid component showing iso- to hypointense signals (shown in **Figure 1B**). The lesion was hyperintense on fluid-attenuated inversion recovery (FLAIR) sequences (shown in **Figure 1C**) and showed no diffusion restriction on diffusion weighted imaging (DWI) (shown in **Figure 1D**). Post-contrast T1-weighted fat-suppressed images demonstrated peripheral rim enhancement and patchy enhancement of solid components. The lesion was surrounded by perilesional oedema, which showed no enhancement on post-contrast scans (shown in **Figures 1E, F**).

**Figure 2:**
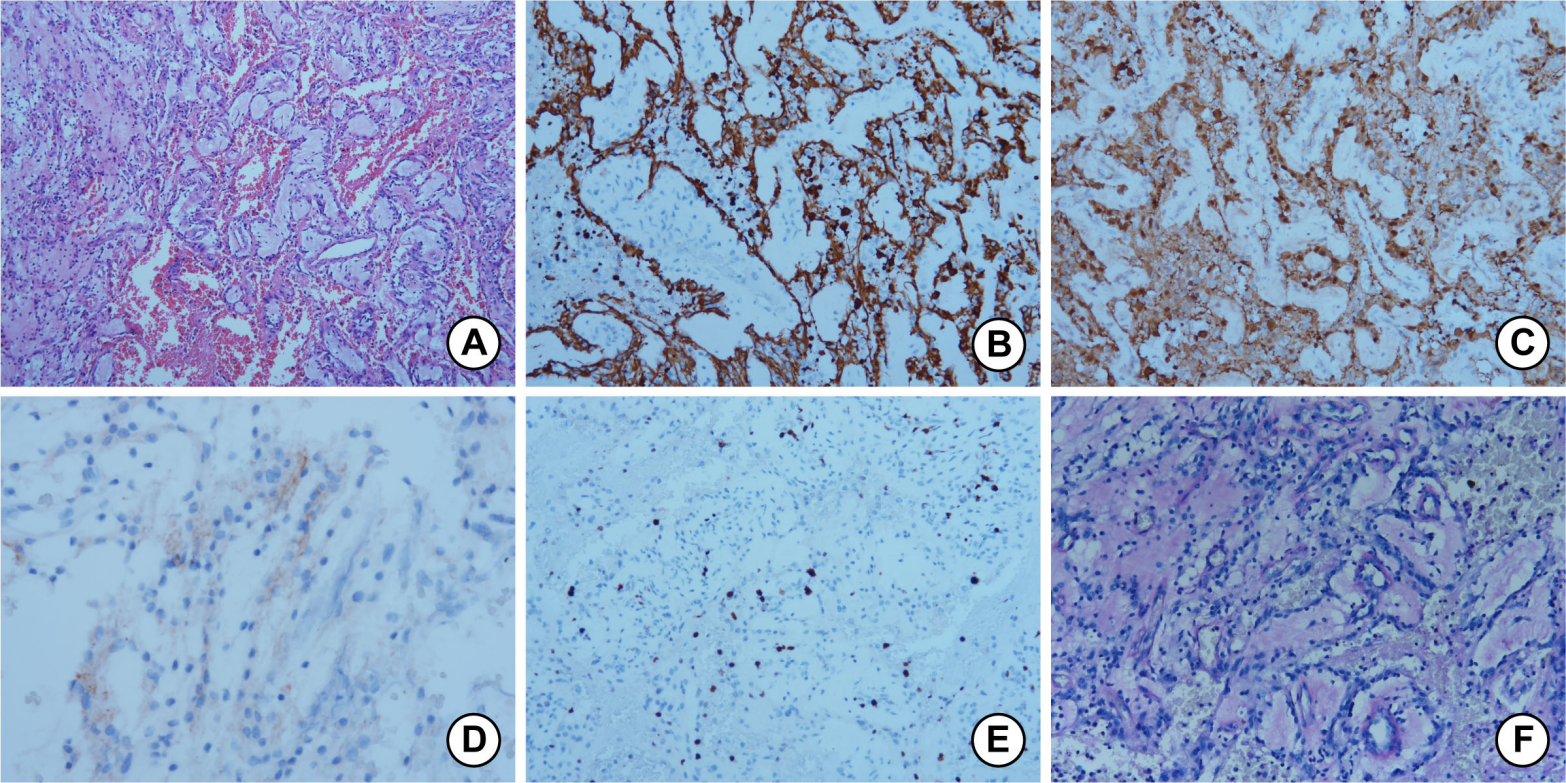
**(A)** The tumor cells formed papillary structures around fibrovascular cores, exhibiting microcapsule formation and mucin aggregation features (HE, 100X). **(B)** GFAP (glial fibrillary acidic protein) immunostaining showing diffuse and intense expression (200X). **(C)** S-100 immunostaining showing diffuse and intense expression (200X). **(D)** EMA (epithelial membrane antigen) immunostaining showing dot-like cytoplasmic expression (400X). **(E)** The Ki-67 labeling index was 3% (200X). **(F)** PAS staining was positive in the myxoid material surrounding the blood vessels, within the tumor cells, and between the tumor cells (200X).

Macroscopically, the tumor presented with a soft, cystic-solid consistency and contained approximately 20 ml of yellow cystic fluid. Histopathological examination revealed two cardinal features: prominent vascularity and distinct border characteristics. The tumor parenchyma exhibited abundant vascular proliferation, characterized by numerous dilated capillary channels. Microscopically, while lacking a definitive fibrous capsule, the neoplasm maintained a well-demarcated interface with adjacent neuropil, demonstrating neither infiltrative growth patterns nor satellitosis at the periphery. The peri-tumor brain parenchyma exhibited marked oedema but lacked evidence of hemorrhage. The neoplastic population consisted predominantly of cuboidal to spindle cells. Most tumor cells formed papillary structures around fibrovascular cores, exhibiting microcapsule formation and mucin aggregation features (shown in **Figure 2A**). Some tumor cells were polygonal and arranged in diffuse sheets, while others were spindle-shaped and organized in fascicular patterns. The tumor cells had abundant eosinophilic cytoplasm, mild atypia, and a minimal number of mitotic figures. Extensive stromal edema and areas of hemorrhage were also observed.

### 2.2 Case 2

A 17-year-old male presented with an acute onset of consciousness disturbance seven years prior, without any obvious precipitating factors. The episode was characterized by a sudden cessation of activity lasting several seconds, accompanied by staring spells and unresponsiveness to verbal stimuli. Postictal amnesia was noted following the episode. The patient experienced dizziness without limb convulsions approximately 1–2 times per month. Over the past year, the frequency of episodes increased, with each lasting over ten seconds and occurring 3–4 times per week. Brain MRI revealed a well-demarcated mass in the left frontal lobe, hypointense on T1-weighted images, hyperintense on T2-weighted sequences, and isointense to hyperintense on FLAIR imaging. The tumor was heterogeneously contrast-enhancing, measured 0.8 × 0.7 cm, and had well-defined margins without evidence of perilesional oedema. The ventricles, cisterns, and sulci were of standard size and configuration, and midline structures were unshifted.

The gross tumor tissue had a gray-white appearance and a soft consistency. Under microscopic examination, the tumor cells appeared cuboidal and were organized in a radial pattern around hyalinized fibrovascular cores, forming a myxopapillary structure. Myxoid material was present between the tumor cells and blood vessels, as well as clustered within microcysts. The tumor cells exhibited abundant eosinophilic cytoplasm with mild atypia, no mitotic figures, and scattered small areas of stromal hemorrhage.

Immunohistochemically, the tumor cells were positive for GFAP (shown in **Figure 2B**), S-100 (shown in **Figure 2C**), SOX-10, Vimentin, Olig-2, Nestin, and ATRX. Immunolabeling revealed dot-like cytoplasmic expression of EMA (show in **Figure 2D**). Synaptophysin was expressed negatively in Case 1 and positively in Case 2. The tumor cells were negative for H3K27M, IDH1 R132H, NeuN, CD34, Cytokeratin (CK), ERG, PR, STAT6, CK7, CK20, CAM5.2, CD99, and EGFR in both cases. The Ki-67 labeling index was 3% in Case 1 (show in **Figure 2E**) and 2% in Case 2. Additionally, the myxoid material surrounding the blood vessels, as well as within and between the tumor cells, showed positive staining with PAS (shown in **Figure 2F**).

Both patients underwent surgical resection of the lesion and did not receive adjuvant radiotherapy. Follow-up was conducted until the time of writing this report. Case 1 had a follow-up period of 26 months, while Case 2 had a follow-up period of 18 months. No evidence of distant metastasis was observed.

## 3 Discussion

MPE is a slow-growing subtype of ependymoma, accounting for 9% to 13% of all cases (5, 6). In the 2016 WHO classification of CNS tumors, MPE was classified as a WHO grade 1 tumor(1). However, subsequent studies revealed instances of postoperative recurrence and distant metastasis, suggesting a more aggressive biological behavior in some cases (7, 8). As a result, the updated WHO classification in 2021 reclassified MPE to grade 2, providing a more accurate assessment of its prognosis and associated risks(2).

MPE originates from remnants of ependymal cells and typically arises in the conus medullaris, cauda equina, and filum terminale (9, 10). Although it can occur at any age, it predominantly affects adults. A SEER Program analysis showed peak incidence in patients aged 25–29 and 45–59 years (4, 8), with a mean age of 36 years and a male-to-female ratio ranging from 1.4 to 2:1 (4). By contrast, primary intracranial MPE is exceedingly rare, and few cases have been reported (11). Among the 17 known cases, only 5 involved the cerebral parenchyma (**Table 1**) (11–26). The mean age of patients with intracranial MPE was 30 years (range, 7-62), with a male-to-female ratio of 1.13:1.

**Table 1:** Summary of 17 reported cases of primary intracranial MPE.

Case	Age	Sex	Primary site	Reference
1	29	Male	Right lateral ventricle	Sato et al. (12)
2	8	Female	Right occipital lobe	Maruyama et al. (13)
3	37	Female	Lateral ventricle	Warnick et al. (14)
4	37	Male	Lateral ventricle	Matyja et al. (15)
5	22	Male	Left temporal lobe	Ralte et al. (16)
6	68	Male	Left frontal lobe	Tzerakis et al. (17)
7	7	Female	Roof of fourth ventricle, upper cerebellar vermis, and adjacent quadrigeminal plate	Tseng et al. (18)
8	20	Female	Bilateral, high convexity, multicystic, attached to the falx	Tseng et al. (18)
9	62	Female	Floor fourth ventricle	Lim et al. (19)
10	30	Male	Left cerebellopontine angle	Sparaco et al. (20)
11	8	Male	Medulla	DiLuna et al. (21)
12	50	Female	Fourth ventricle	Chakraborti et al. (22)
13	50	Female	Fourth ventricle 14	Khalatbari et al. (23)
14	21	Female	Right frontal lobe 16	Patangia et al. (24)
15	14	Male	Right occipital lobe 17	Mewada et al. (25)
16	32	Male	Fourth ventricle 18	Kumaria et al. (26)
17	31	Female	Fourth ventricle 24	Mldie et al. (11)

The clinical presentation of intracranial MPE depends on tumor location and size, and may include headaches, seizures, visual disturbances, nausea, vomiting, intratumoral hemorrhage, and balance issues. Interestingly, even tumors in the same lobe can manifest different symptoms, as shown in our two cases. Case 1 presented with an acute-onset headache, while Case 2 exhibited a seven-year history of intermittent seizures. These differences may be attributed to the proximity of the tumor to eloquent cortical areas and the extent of functional brain involvement. The prolonged symptom duration in some cases suggests a slow-growing tumor phenotype, consistent with prior reports (shown in **Table 2**).

**Table 2:** Summary of 5 reported cases of cerebral parenchyma MPE.

Case	Primary site	Size	Nature	Symptoms	Duration of symptoms	Reference
1	Right occipital lobe	No data available	No data available	No data available	No data available	Maruyama et al. (13)
2	Left temporal lobe	2.3x1.8cm^*^	solid	recurrent complex partial seizures with secondary generalization	2 years	Ralte et al. (16)
3	Left frontal lobe	5.9x5.5cm^*^	solid cystic	dizziness, memory disturbance, confusion, disorientation in time and place	10 days	Tzerakis et al. (17)
4	Right frontal lobe	6.2x5.9cm	cystic	headache and vomiting	Immediately	Patangia et al. (24)
5	Right occipital lobe	2.3x1.8cm^*^	solid	seizures	6 months	Mewada et al. (25)

*The literature did not provide a specific description of the tumor’s dimensions. Using the available imaging data, we made a reasoned estimate of the tumor’s size.

Radiological and pathological evaluations are the cornerstone for diagnosing intracranial MPE. Notably, intracranial metastasis from spinal MPE must be excluded before confirming a primary intracranial origin (22, 26). MPE typically appears as a well-circumscribed mass with T1 hypointensity, T2 hyperintensity, and marked post-contrast enhancement (27). Additional imaging findings, such as cystic degeneration and hemorrhage, may also be observed. Among the 7 reported parenchymal MPE cases, all demonstrated T1 hypointensity and T2/FLAIR hyperintensity, and most were well-circumscribed. Only one case exhibited an ill-defined cystic mass.

The macroscopic appearance of spinal MPE is typically oval, sausage-shaped, or lobulated, with soft consistency and gray-tan color. These lesions are often encapsulated and may show gelatinous change, cyst formation, or hemorrhage. Similar features were also observed in our intracranial cases, especially in the larger lesions.

Histologically, MPE displays cuboidal to spindle-shaped tumor cells arranged in papillary structures around hyalinized fibrovascular cores, with myxoid material surrounding blood vessels and accumulating in microcysts. These characteristic features may result from chronic hypoxia, interstitial mucinous degeneration, and the secretion of mucin by tumor cells (28). Secondary changes include fibrosis, hemorrhage, and hemosiderin deposition, while rare cases may exhibit cartilaginous metaplasia (22). Some tumors lack papillary architecture and instead consist of polygonal sheets or fascicles of spindle cells (2). Other patterns include microcystic changes, cribriform structures, tubular or slit-like formations, and areas of pleomorphic giant cells (29). Identification of PAS- or Alcian blue-positive myxoid material is essential for recognizing tumors with minimal papillary features.

Tumor cells are typically bland, with well-defined borders, eosinophilic or hyaline cytoplasm, and oval or short spindle-shaped nuclei with small nucleoli. Mitotic activity is low, with Ki-67 labeling indices generally below 2–3% (30). Rare anaplastic variants exhibit hypercellularity and reduced mucin. To be classified as anaplastic, the tumor must meet at least two of the following criteria: ≥2 mitoses per mm², Ki-67 index >10%, microvascular proliferation, or spontaneous necrosis (31).

Case 1 exhibited diverse morphologies, including classic myxopapillary areas, spindle bundles, cystic degeneration, and hemorrhage, whereas Case 2 presented with uniform papillary structures. These differences may be associated with tumor size, as larger tumors tend to exhibit more heterogeneous features. Both cases showed low proliferation indices and positive PAS staining, supporting a non-anaplastic diagnosis.

Immunohistochemically, MPE is typically positive for GFAP, S100, and Vimentin. Dot-like cytoplasmic EMA expression is absent, while OLIG-2 nuclear staining is negative. CD99 and CD56 may also be expressed (32). MPEs are often negative for CAM5.2, CK5/6, CK7, and CK20 (33). Although CK expressions are variable in previous literature, the absence of CK in our cases is consistent with classical profiles.

Primary intracranial MPE should be distinguished from several tumors with overlapping histological features: (a) Metastatic papillary carcinoma: shows pseudopapillary structures, marked atypia, increased mitotic activity, and CK positivity. Unlike MPE, these tumors express epithelial and site-specific markers but lack GFAP, S100, and Vimentin. (b) Chordoma, myxoid chondrosarcoma, and chordoid meningioma are characterized by abundant extracellular mucin. Immunohistochemistry (using GFAP, CK, EMA, and D2-40) assists in differentiation. Chordomas have vacuolated cytoplasm and express CK, EMA, but not GFAP or D2-40. Myxoid chondrosarcomas often arise in soft tissue and only express D2-40. Chordoid meningiomas exhibit eosinophilic cytoplasm, vacuoles, and a trabecular or lobular pattern, but they express EMA and PR, not GFAP or CK. (c) Paraganglioma: composed of epithelioid cells with Synaptophysin positivity in main cells but GFAP and S100 positivity only in supporting cells. (d) Schwannoma: presents with microcystic or reticular architecture, hemorrhage, and hemosiderin. Lacks papillary pattern; cells may be palisaded with Verocay bodies. GFAP shows focal expression, and reticulin staining is positive. (e) Papillary ependymoma: rare; exhibits perivascular pseudorosettes in a pseudopapillary pattern but lacks myxoid material. (f) Choroid plexus papilloma: features actual papillary structures with fibrovascular cores. Tumor cells are CK and CK7 positive, may express GFAP, but not EMA. (g) Chordoid glioma: occurs mainly in the third ventricle or parenchyma. Shows a chordoid structure with mucinous stroma and lymphoplasmacytic infiltration. Immunoprofile includes CD34, CK, TTF-1, and a characteristic PRKCA D463H mutation.

Most MPEs are diagnosed when patients exhibit clinical symptoms, and no documented cases of familial hereditary MPE have been reported. Research indicates that MPE typically exhibits tetraploid or aneuploid characteristics. Comparative genomic hybridization (CGH) analysis has revealed a significantly higher number of MPE chromosomal variants than conventional ependymomas, accompanied by extensive chromosomal imbalances (34). To further characterize the molecular features of ependymomas, a study utilizing DNA methylation profiles analyzed various diseases and identified nine molecular groups of ependymomas across three anatomical locations: the supratentorial compartment, the posterior fossa, and the spinal compartment (35). MPEs were classified within one of the molecular groups associated with the spinal compartment. These tumors exhibited polyploidy, showed chromosomal gains across multiple loci, and were associated with an excellent prognosis. Research has revealed that the expression levels of the Neurofilament light polypeptide (NEFL), Homeobox B13 (HOXB13), and Platelet-Derived Growth Factor Receptor alpha (PDGFRα) genes were significantly upregulated in MPE compared with ordinary ependymomas, suggesting that HOXB13 may play a critical role in promoting MPE development. In addition, PDGFRα could be a potential therapeutic target for recurrent MPE (36). Furthermore, a study found that Hox transcript antisense RNA (HOTAIR) and its downstream HOX genes were expressed at considerably higher levels in MPE than in other spinal cord and intracranial ependymal tumors. These findings suggest that HOTAIR and its downstream HOX genes may serve as unique epigenetic markers for MPE and contribute to its tumorigenesis (37).

Surgical resection remains the cornerstone of treatment for MPE. Total or subtotal excision offers favorable long-term outcomes, with 10-year survival rates approaching 90% (8, 38). Adjuvant radiotherapy may enhance progression-free survival, particularly in cases with subtotal resection or atypical features (4).

## 4 Conclusion

Primary intracranial myxopapillary ependymoma (MPE) is an exceedingly rare clinical entity, with only a limited number of cases reported to date. Its presentation is variable and largely dependent on tumor location, with manifestations such as headache or seizures, as observed in our two cases involving the frontal lobe. MRI remains the cornerstone of radiologic assessment, typically demonstrating a well-circumscribed lesion with distinct signal characteristics. Histopathological examination is essential for definitive diagnosis and requires careful differentiation from other papillary or myxoid CNS neoplasms.

Given the tumor’s histological resemblance to a range of other intracranial pathologies, an integrated diagnostic approach—combining imaging, immunohistochemistry, and molecular profiling—is critical. In both of our cases, gross total resection was achieved without adjuvant therapy, and no recurrence or metastasis was observed during follow-up. Although the long-term outcomes of primary intracranial MPE remain under-investigated due to its rarity, the existing literature and our findings suggest a generally favorable prognosis, particularly when complete surgical excision is feasible.

Further accumulation of case data and molecular analyzes will be essential to deepen our understanding of this tumor subtype and to refine its diagnostic and therapeutic framework.

## Data Availability

The original contributions presented in the study are included in the article/**Supplementary Material**. Further inquiries can be directed to the corresponding authors.
