# Supplementary figures and images for: Primary intracranial myxopapillary ependymoma: two case reports and literature review (part 1 of 2)
Source: Front Oncol. 2026 May 21;16:1763325. doi: 10.3389/fonc.2026.1763325 (PMC13233268; doi:10.3389/fonc.2026.1763325)

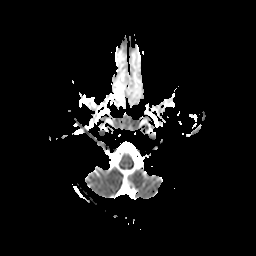

Supplement: Supplementary file 1 [file DataSheet1.zip › MRI-ADC/ADC-0.tif]

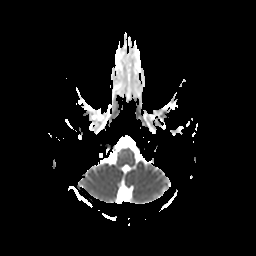

Supplement: Supplementary file 1 [file DataSheet1.zip › MRI-ADC/ADC-1.tif]

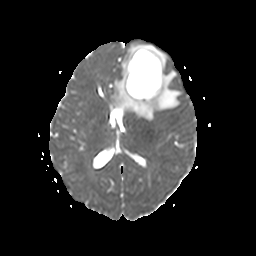

Supplement: Supplementary file 1 [file DataSheet1.zip › MRI-ADC/ADC-10.tif]

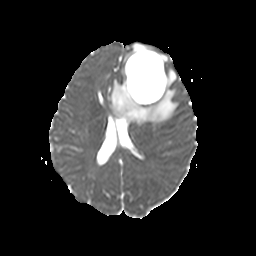

Supplement: Supplementary file 1 [file DataSheet1.zip › MRI-ADC/ADC-11.tif]

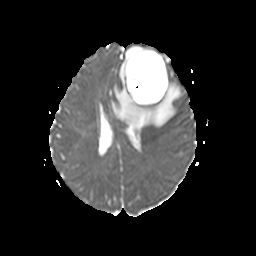

Supplement: Supplementary file 1 [file DataSheet1.zip › MRI-ADC/ADC-12.tif]

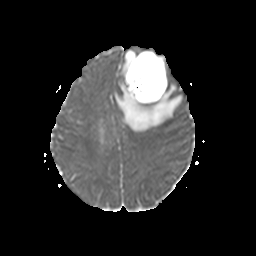

Supplement: Supplementary file 1 [file DataSheet1.zip › MRI-ADC/ADC-13.tif]

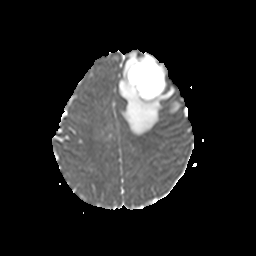

Supplement: Supplementary file 1 [file DataSheet1.zip › MRI-ADC/ADC-14.tif]

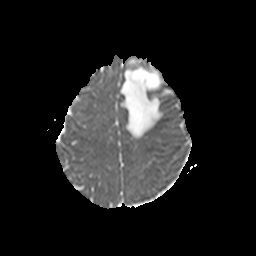

Supplement: Supplementary file 1 [file DataSheet1.zip › MRI-ADC/ADC-15.tif]

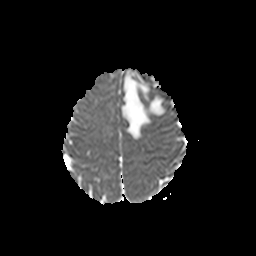

Supplement: Supplementary file 1 [file DataSheet1.zip › MRI-ADC/ADC-16.tif]

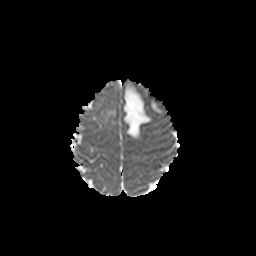

Supplement: Supplementary file 1 [file DataSheet1.zip › MRI-ADC/ADC-17.tif]

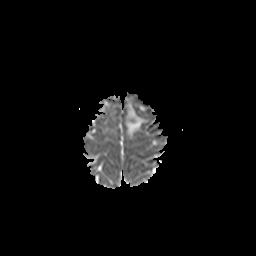

Supplement: Supplementary file 1 [file DataSheet1.zip › MRI-ADC/ADC-18.tif]

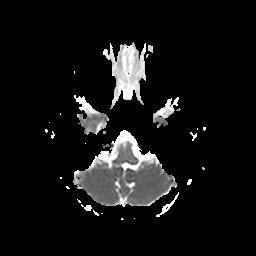

Supplement: Supplementary file 1 [file DataSheet1.zip › MRI-ADC/ADC-2.tif]

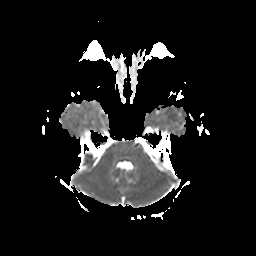

Supplement: Supplementary file 1 [file DataSheet1.zip › MRI-ADC/ADC-3.tif]

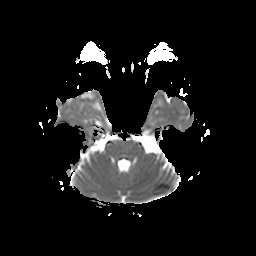

Supplement: Supplementary file 1 [file DataSheet1.zip › MRI-ADC/ADC-4.tif]

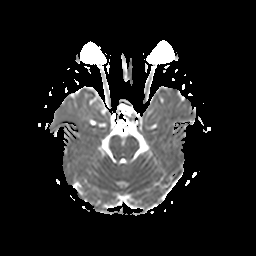

Supplement: Supplementary file 1 [file DataSheet1.zip › MRI-ADC/ADC-5.tif]

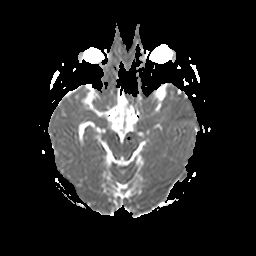

Supplement: Supplementary file 1 [file DataSheet1.zip › MRI-ADC/ADC-6.tif]

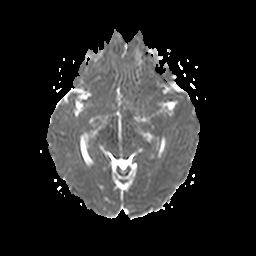

Supplement: Supplementary file 1 [file DataSheet1.zip › MRI-ADC/ADC-7.tif]

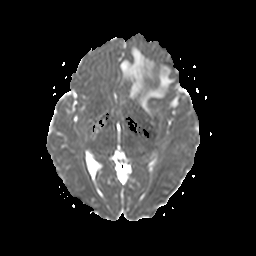

Supplement: Supplementary file 1 [file DataSheet1.zip › MRI-ADC/ADC-8.tif]

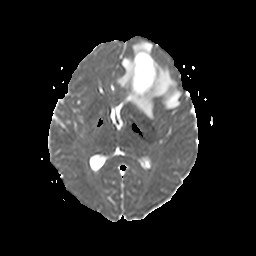

Supplement: Supplementary file 1 [file DataSheet1.zip › MRI-ADC/ADC-9.tif]

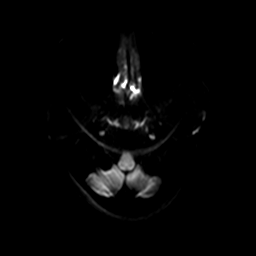

Supplement: Supplementary file 2 [file DataSheet2.zip › MRI-DWI/DWI-0.tif]

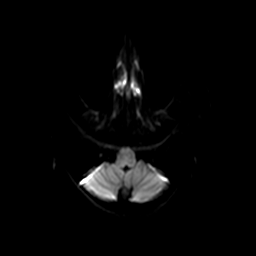

Supplement: Supplementary file 2 [file DataSheet2.zip › MRI-DWI/DWI-1.tif]

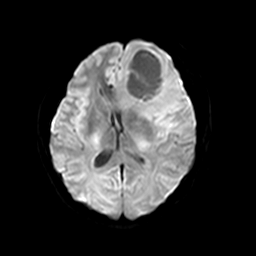

Supplement: Supplementary file 2 [file DataSheet2.zip › MRI-DWI/DWI-10.tif]

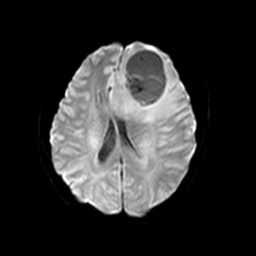

Supplement: Supplementary file 2 [file DataSheet2.zip › MRI-DWI/DWI-11.tif]

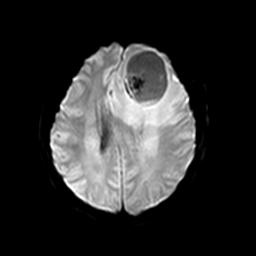

Supplement: Supplementary file 2 [file DataSheet2.zip › MRI-DWI/DWI-12.tif]

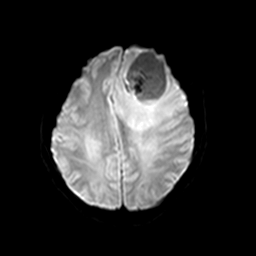

Supplement: Supplementary file 2 [file DataSheet2.zip › MRI-DWI/DWI-13.tif]

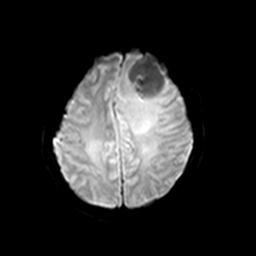

Supplement: Supplementary file 2 [file DataSheet2.zip › MRI-DWI/DWI-14.tif]

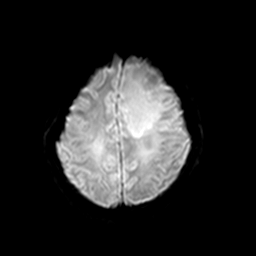

Supplement: Supplementary file 2 [file DataSheet2.zip › MRI-DWI/DWI-15.tif]

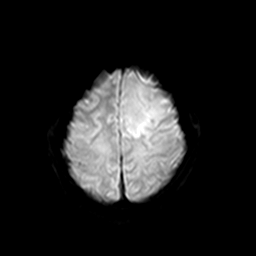

Supplement: Supplementary file 2 [file DataSheet2.zip › MRI-DWI/DWI-16.tif]

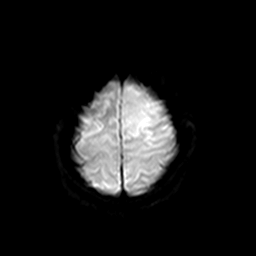

Supplement: Supplementary file 2 [file DataSheet2.zip › MRI-DWI/DWI-17.tif]

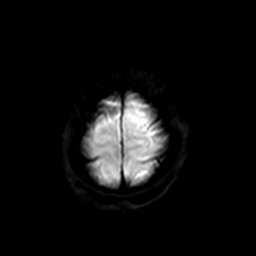

Supplement: Supplementary file 2 [file DataSheet2.zip › MRI-DWI/DWI-18.tif]

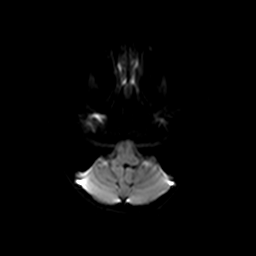

Supplement: Supplementary file 2 [file DataSheet2.zip › MRI-DWI/DWI-2.tif]

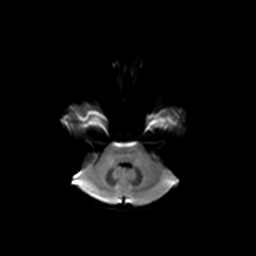

Supplement: Supplementary file 2 [file DataSheet2.zip › MRI-DWI/DWI-3.tif]

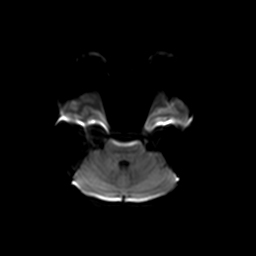

Supplement: Supplementary file 2 [file DataSheet2.zip › MRI-DWI/DWI-4.tif]

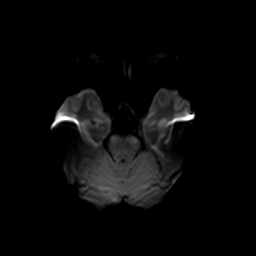

Supplement: Supplementary file 2 [file DataSheet2.zip › MRI-DWI/DWI-5.tif]

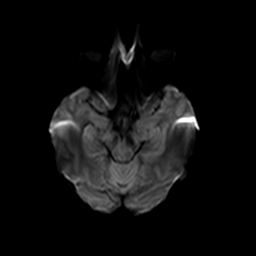

Supplement: Supplementary file 2 [file DataSheet2.zip › MRI-DWI/DWI-6.tif]

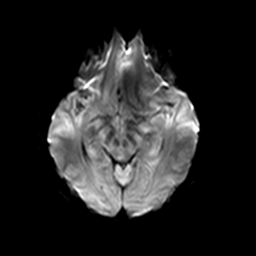

Supplement: Supplementary file 2 [file DataSheet2.zip › MRI-DWI/DWI-7.tif]

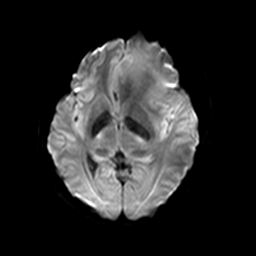

Supplement: Supplementary file 2 [file DataSheet2.zip › MRI-DWI/DWI-8.tif]

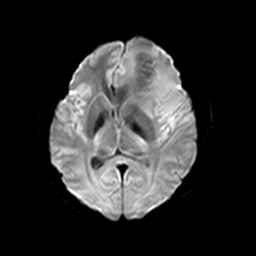

Supplement: Supplementary file 2 [file DataSheet2.zip › MRI-DWI/DWI-9.tif]

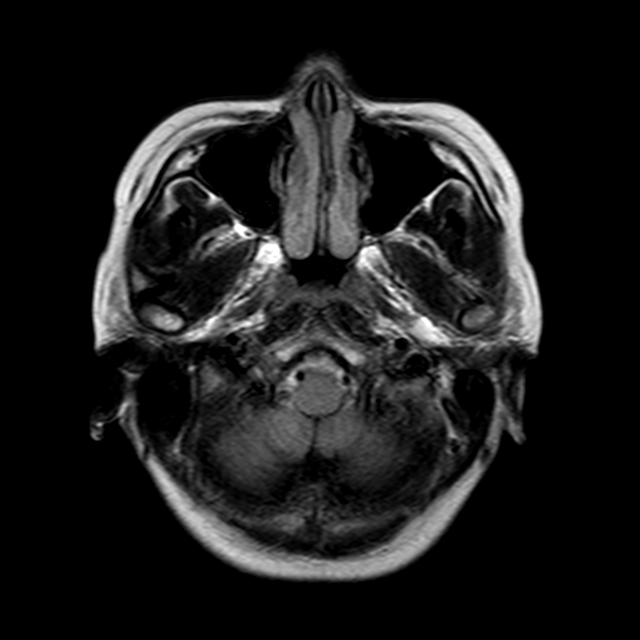

Supplement: Supplementary file 3 [file DataSheet3.zip › MRI-FLAIR/FLAIR-0.tif]

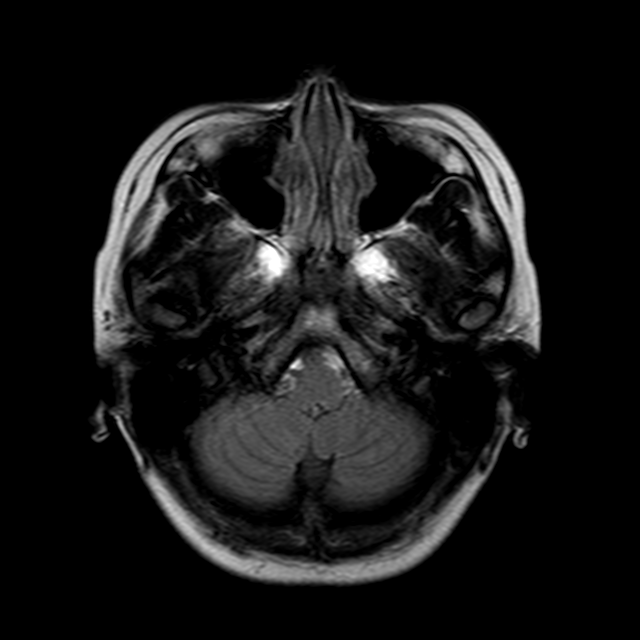

Supplement: Supplementary file 3 [file DataSheet3.zip › MRI-FLAIR/FLAIR-1.tif]

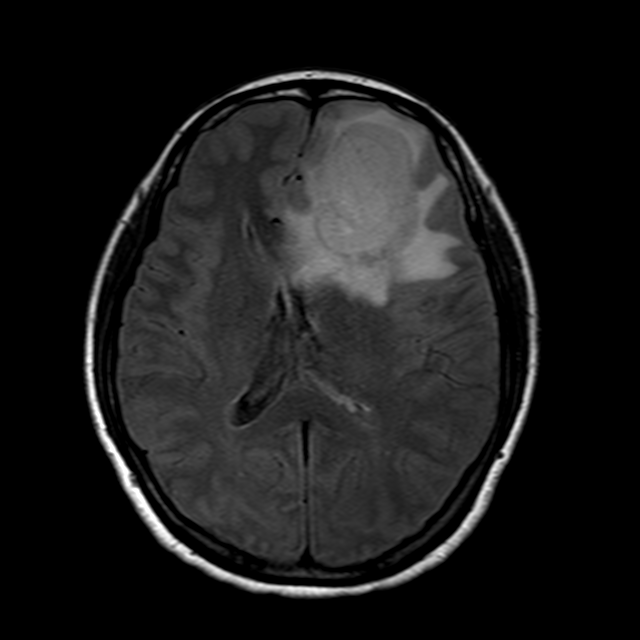

Supplement: Supplementary file 3 [file DataSheet3.zip › MRI-FLAIR/FLAIR-10.tif]

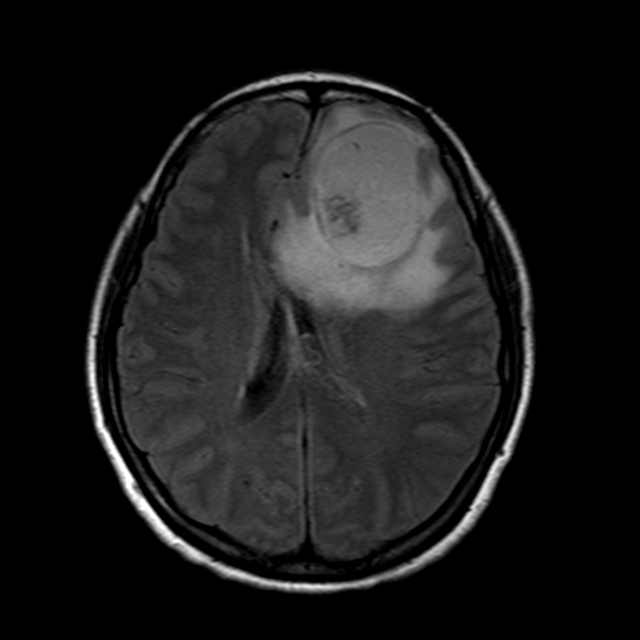

Supplement: Supplementary file 3 [file DataSheet3.zip › MRI-FLAIR/FLAIR-11.tif]

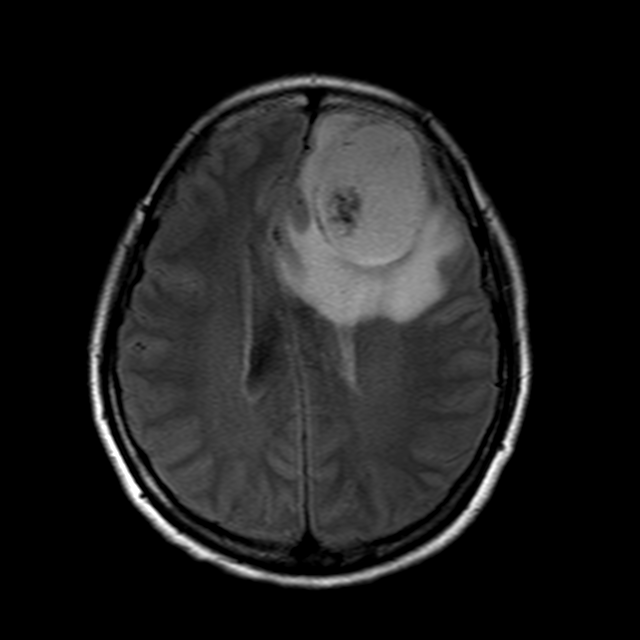

Supplement: Supplementary file 3 [file DataSheet3.zip › MRI-FLAIR/FLAIR-12.tif]

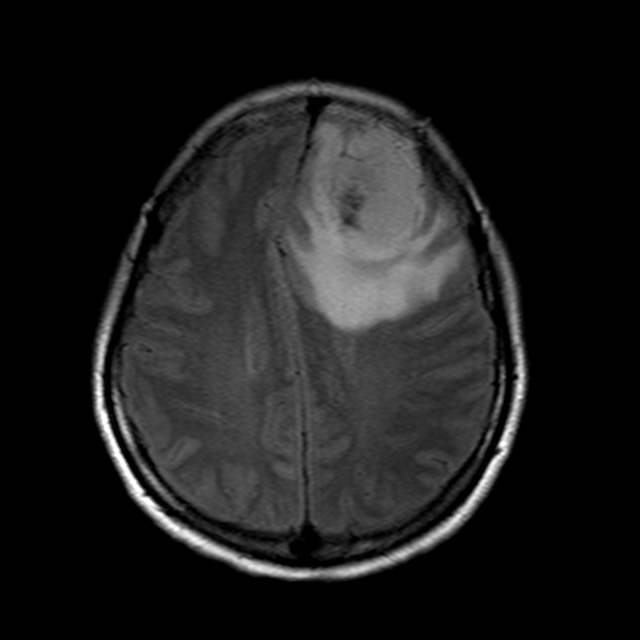

Supplement: Supplementary file 3 [file DataSheet3.zip › MRI-FLAIR/FLAIR-13.tif]

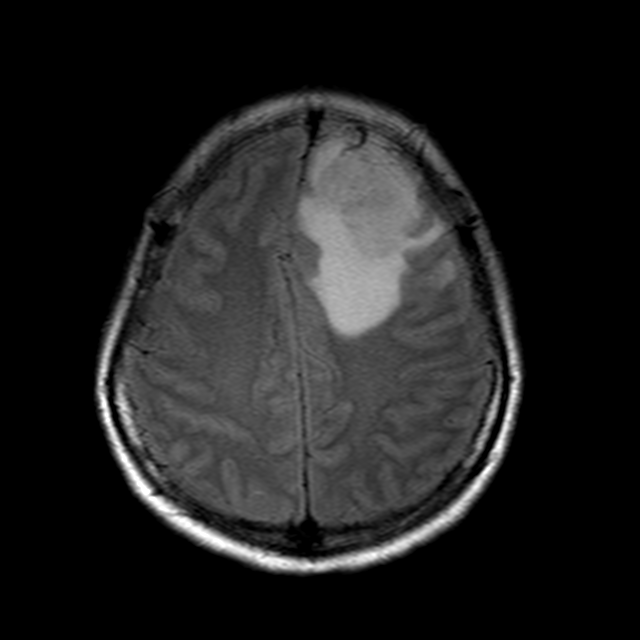

Supplement: Supplementary file 3 [file DataSheet3.zip › MRI-FLAIR/FLAIR-14.tif]

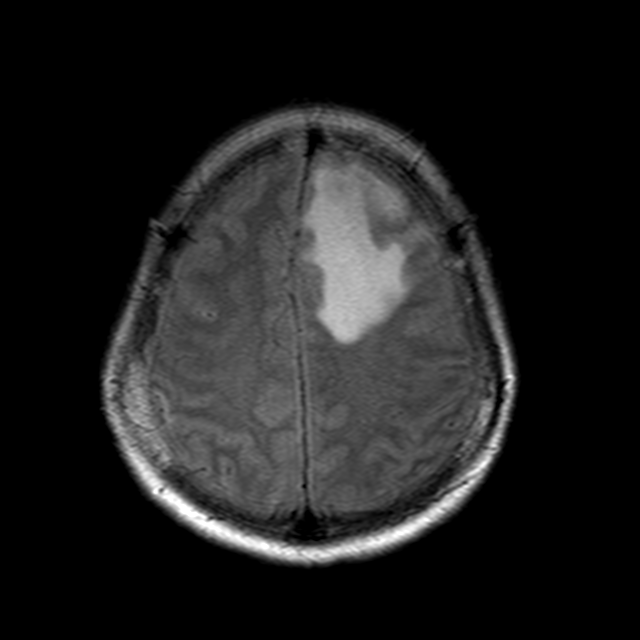

Supplement: Supplementary file 3 [file DataSheet3.zip › MRI-FLAIR/FLAIR-15.tif]

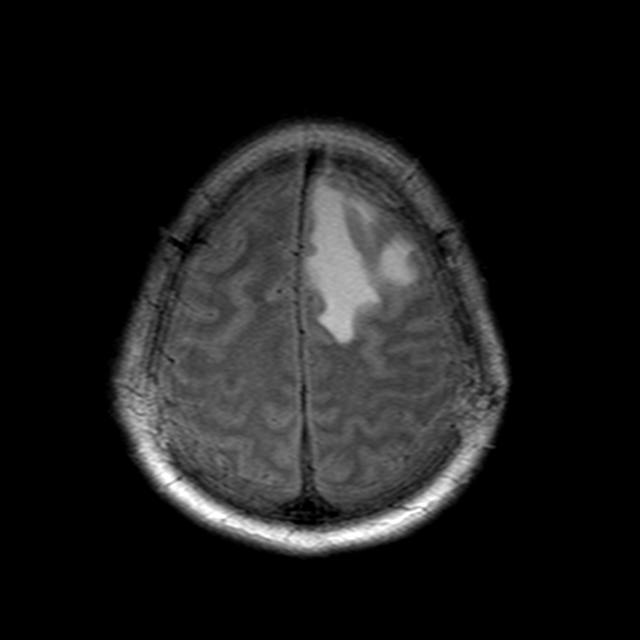

Supplement: Supplementary file 3 [file DataSheet3.zip › MRI-FLAIR/FLAIR-16.tif]

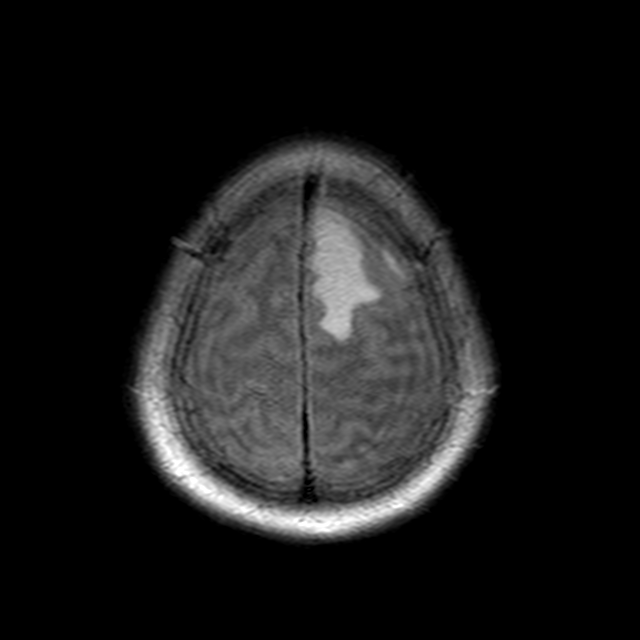

Supplement: Supplementary file 3 [file DataSheet3.zip › MRI-FLAIR/FLAIR-17.tif]

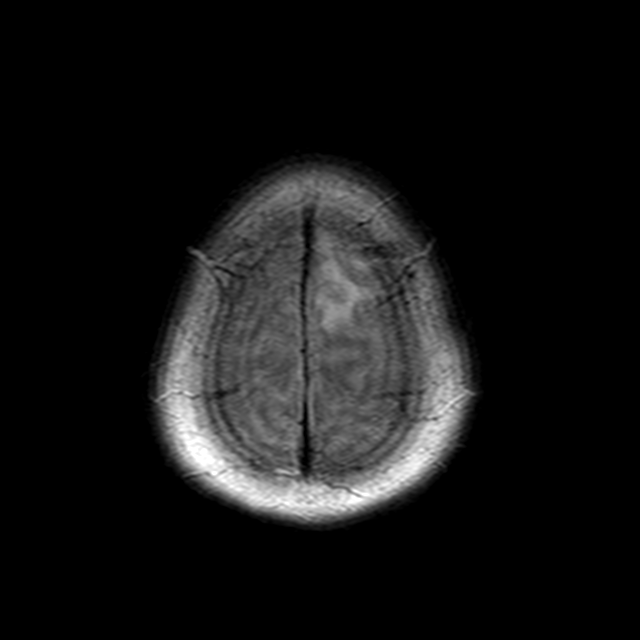

Supplement: Supplementary file 3 [file DataSheet3.zip › MRI-FLAIR/FLAIR-18.tif]

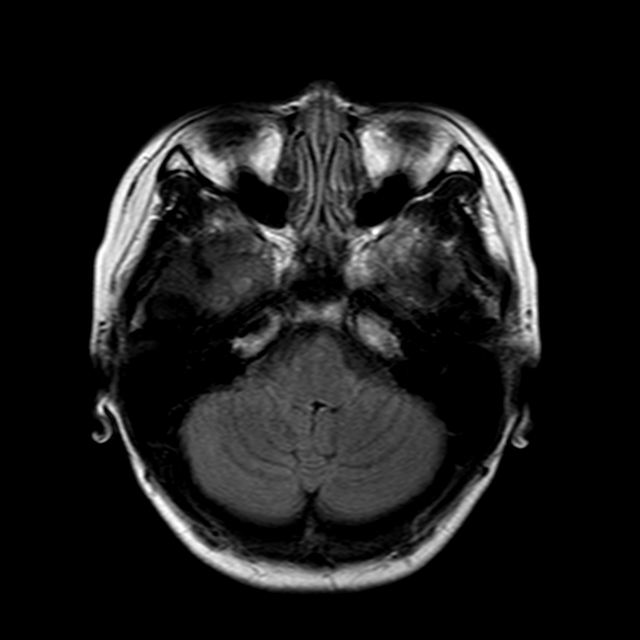

Supplement: Supplementary file 3 [file DataSheet3.zip › MRI-FLAIR/FLAIR-2.tif]

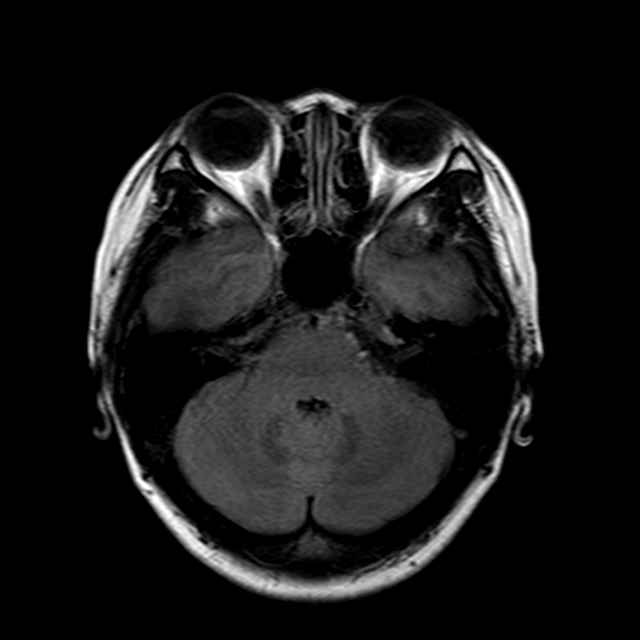

Supplement: Supplementary file 3 [file DataSheet3.zip › MRI-FLAIR/FLAIR-3.tif]

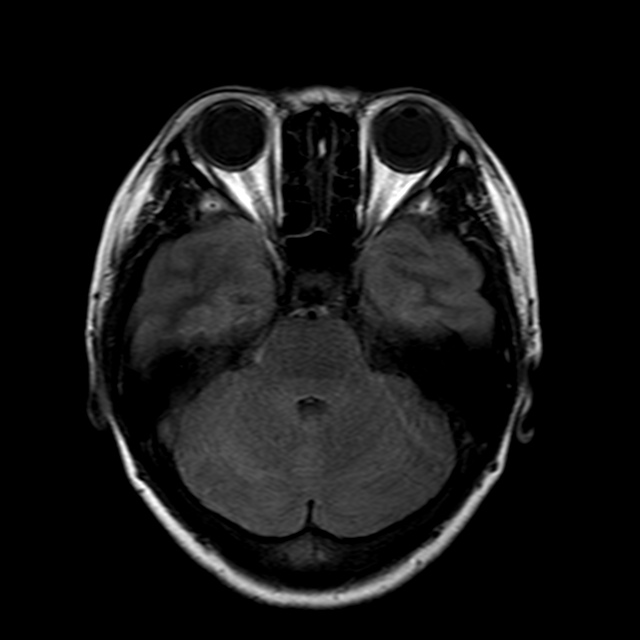

Supplement: Supplementary file 3 [file DataSheet3.zip › MRI-FLAIR/FLAIR-4.tif]

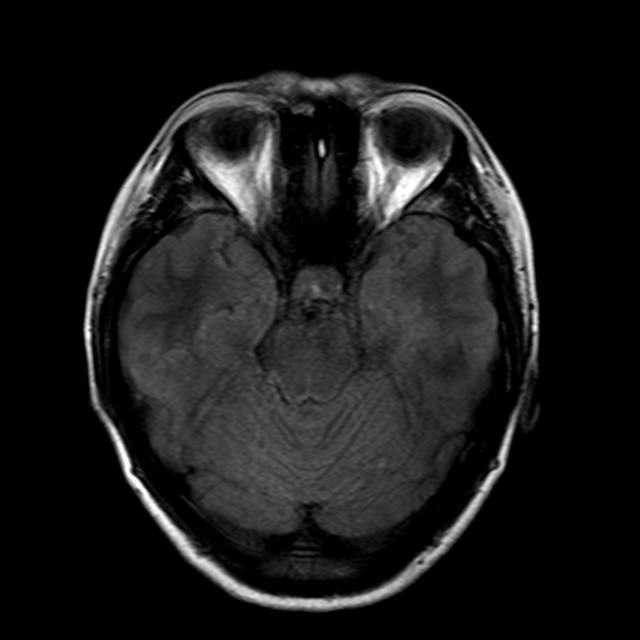

Supplement: Supplementary file 3 [file DataSheet3.zip › MRI-FLAIR/FLAIR-5.tif]

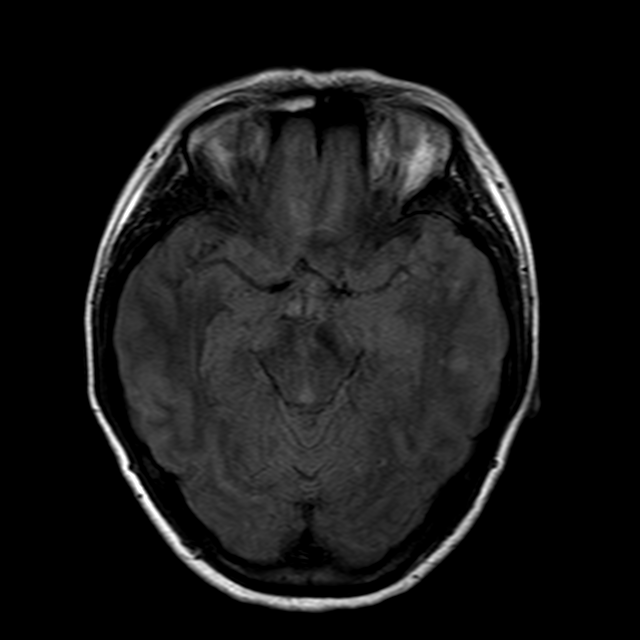

Supplement: Supplementary file 3 [file DataSheet3.zip › MRI-FLAIR/FLAIR-6.tif]

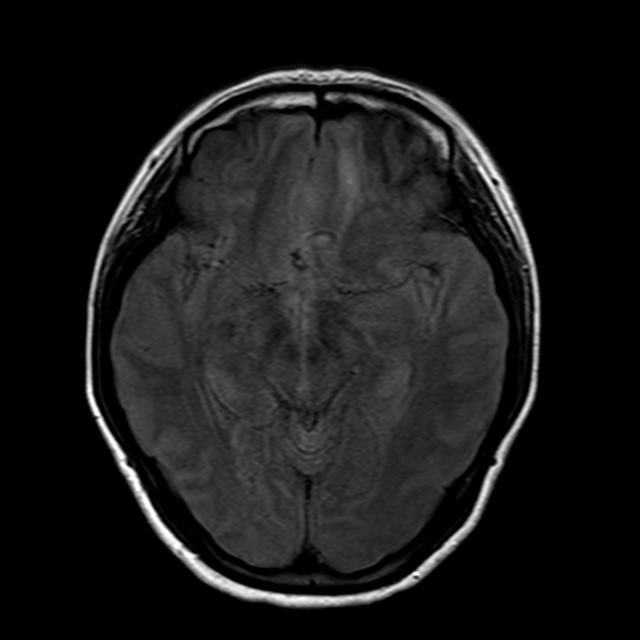

Supplement: Supplementary file 3 [file DataSheet3.zip › MRI-FLAIR/FLAIR-7.tif]

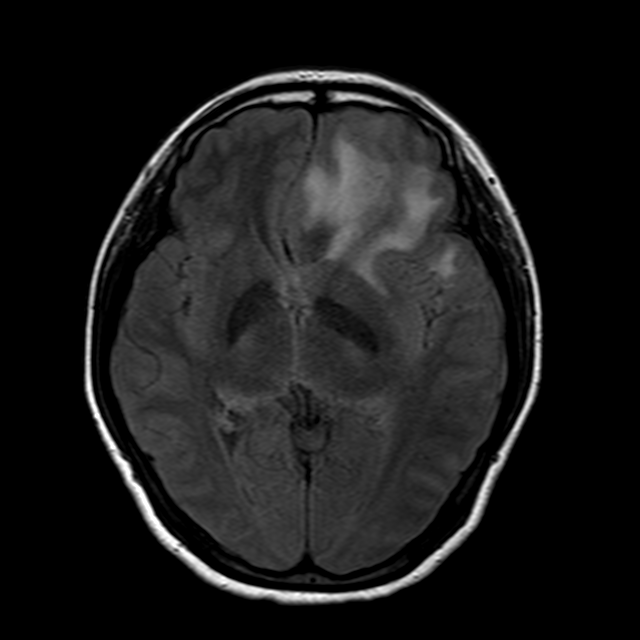

Supplement: Supplementary file 3 [file DataSheet3.zip › MRI-FLAIR/FLAIR-8.tif]

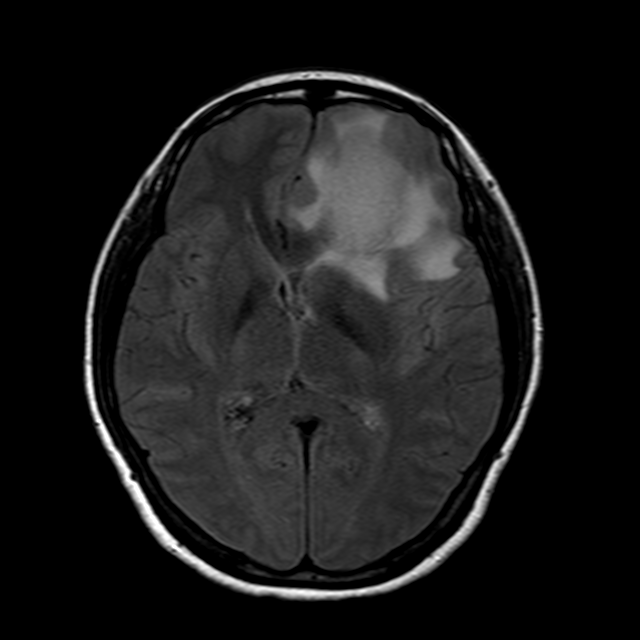

Supplement: Supplementary file 3 [file DataSheet3.zip › MRI-FLAIR/FLAIR-9.tif]

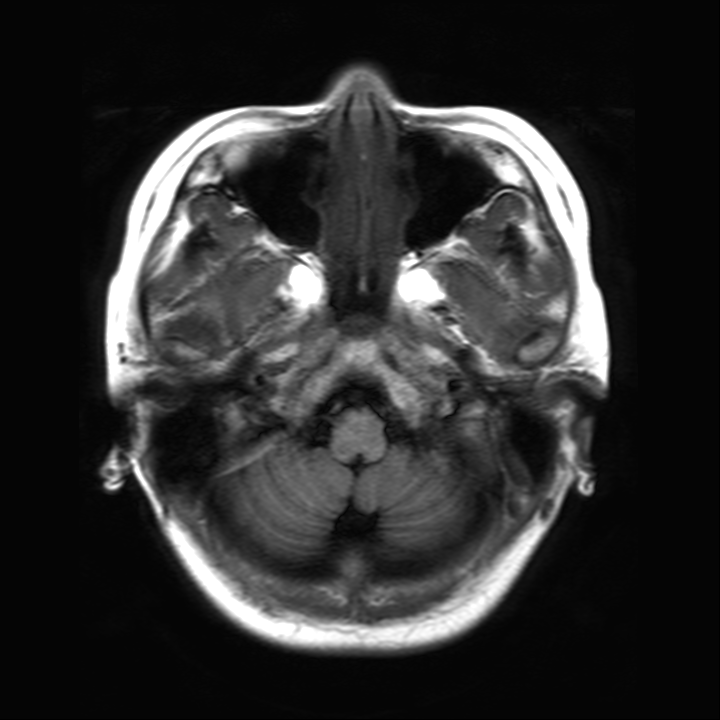

Supplement: Supplementary file 4 [file DataSheet4.zip › MRI-T1/T1-0.tif]

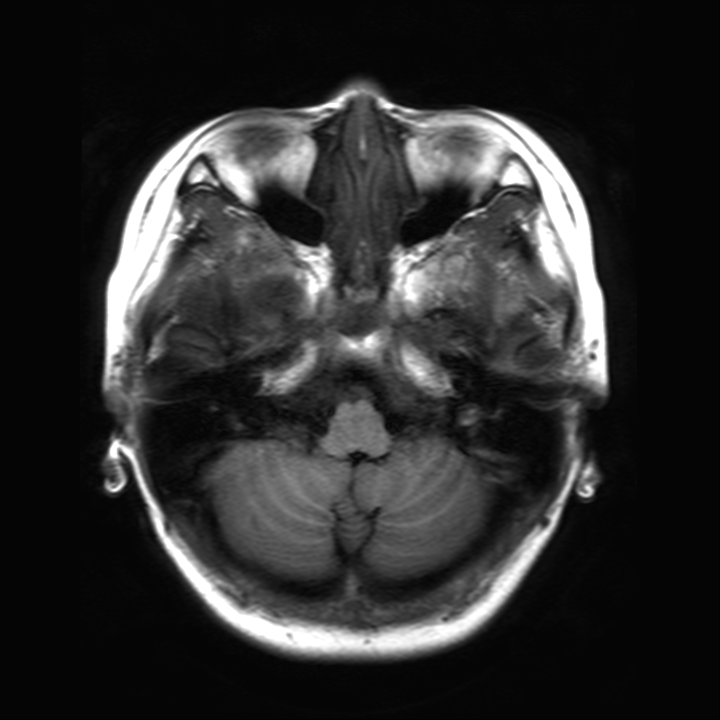

Supplement: Supplementary file 4 [file DataSheet4.zip › MRI-T1/T1-1.tif]

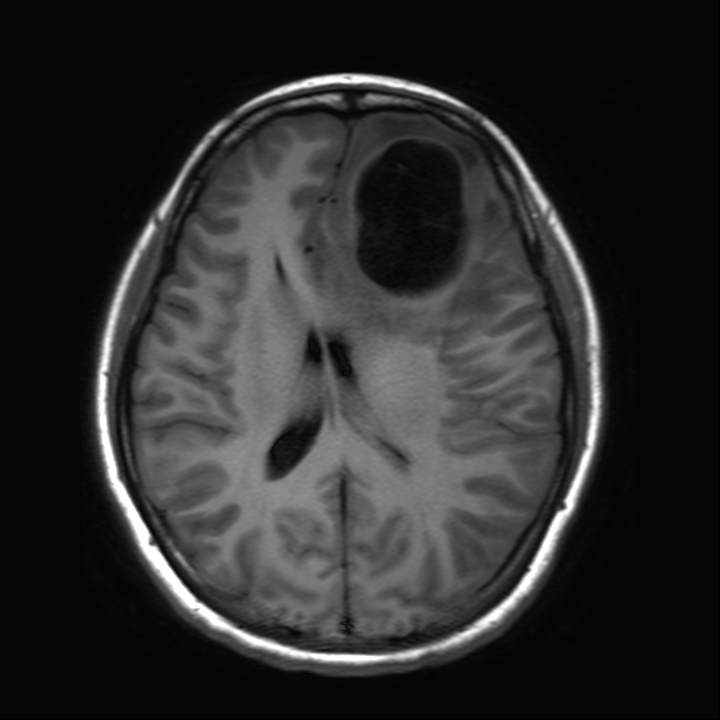

Supplement: Supplementary file 4 [file DataSheet4.zip › MRI-T1/T1-10.tif]

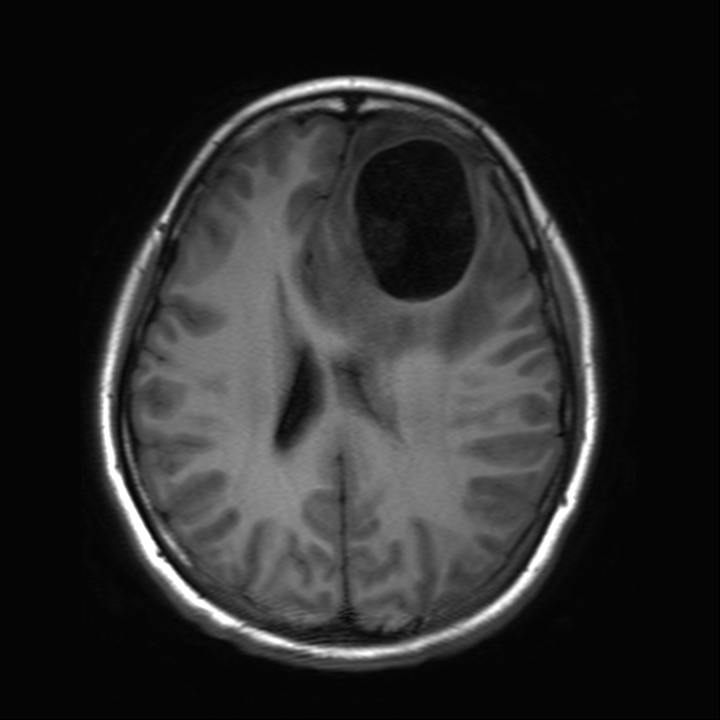

Supplement: Supplementary file 4 [file DataSheet4.zip › MRI-T1/T1-11.tif]

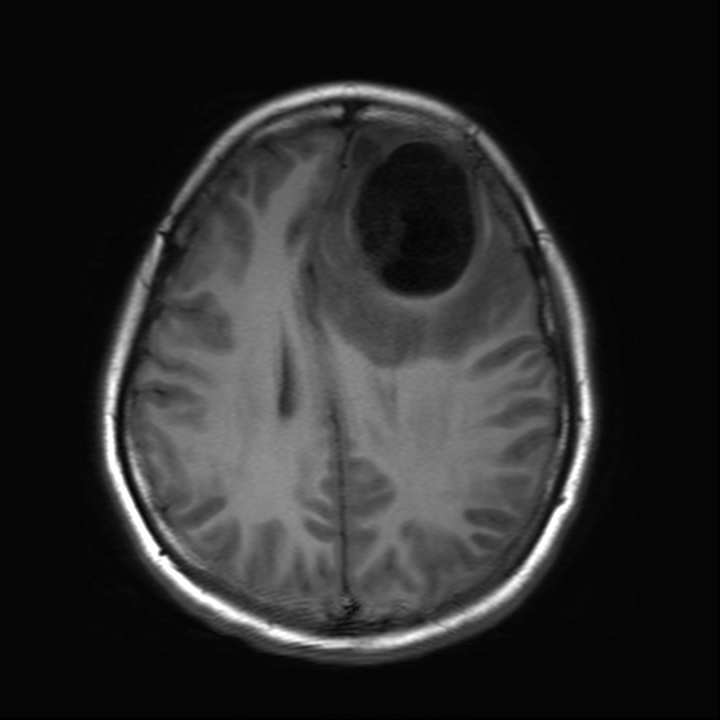

Supplement: Supplementary file 4 [file DataSheet4.zip › MRI-T1/T1-12.tif]

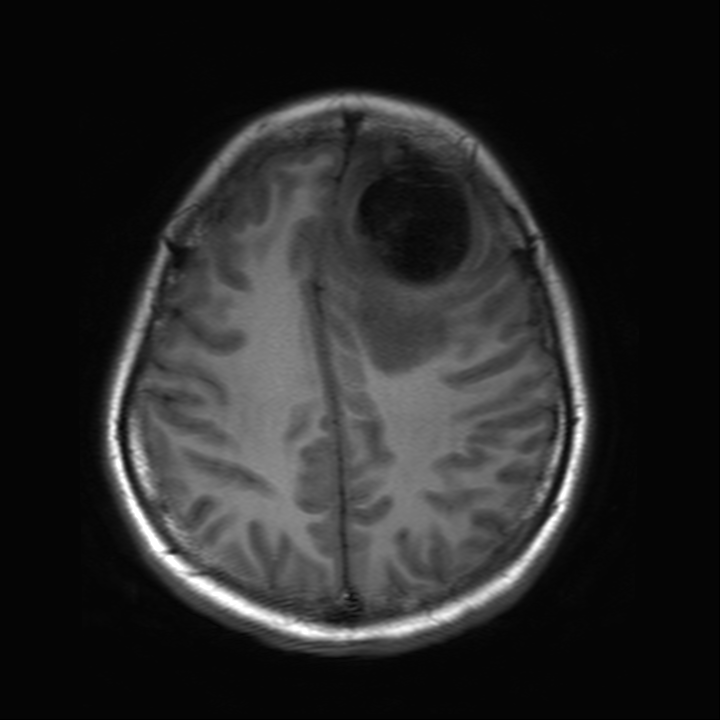

Supplement: Supplementary file 4 [file DataSheet4.zip › MRI-T1/T1-13.tif]

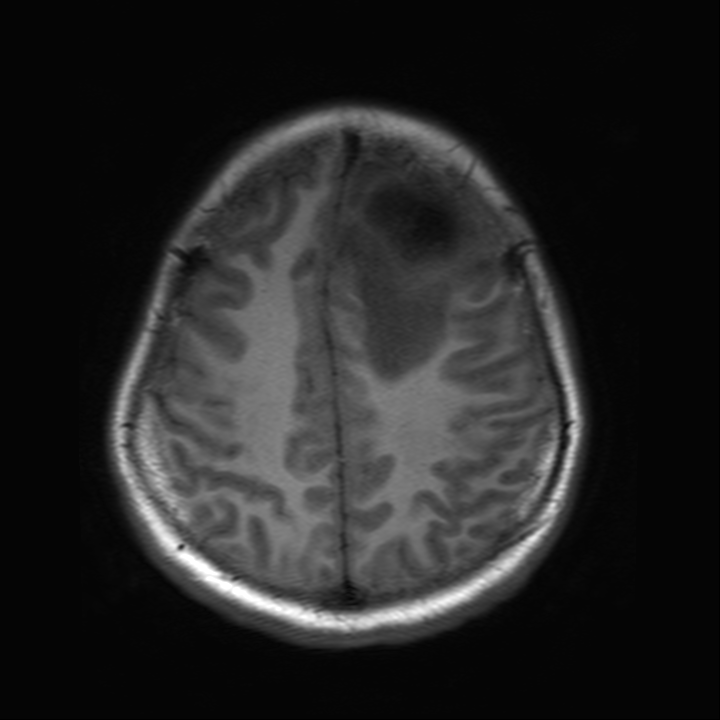

Supplement: Supplementary file 4 [file DataSheet4.zip › MRI-T1/T1-14.tif]

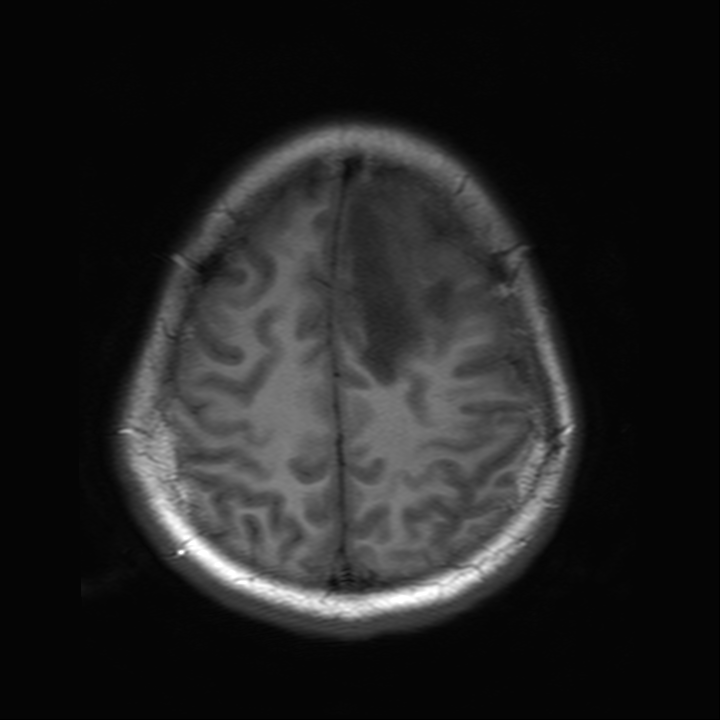

Supplement: Supplementary file 4 [file DataSheet4.zip › MRI-T1/T1-15.tif]

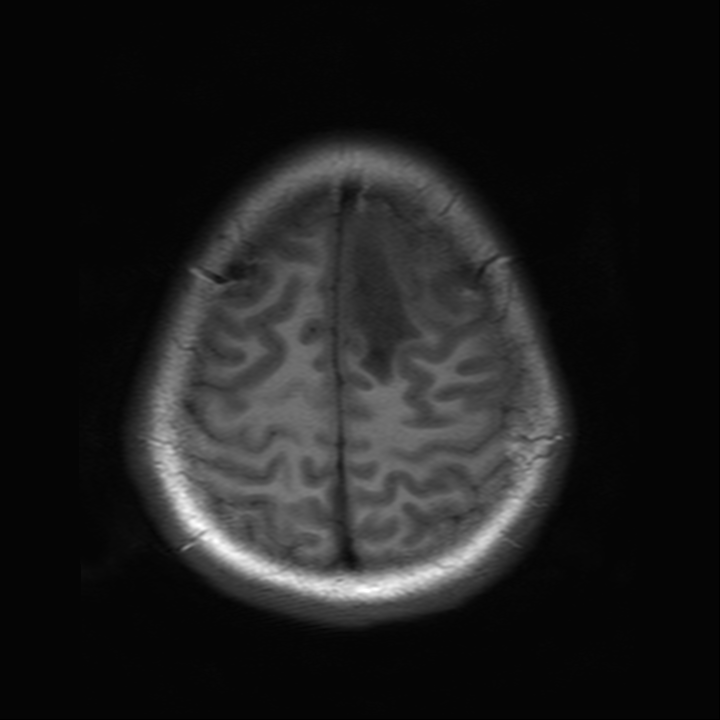

Supplement: Supplementary file 4 [file DataSheet4.zip › MRI-T1/T1-16.tif]

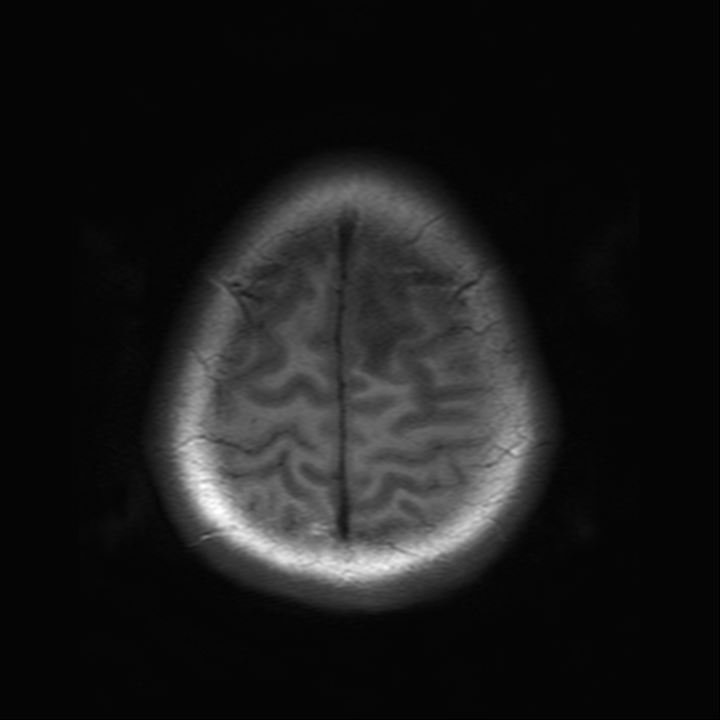

Supplement: Supplementary file 4 [file DataSheet4.zip › MRI-T1/T1-17.tif]

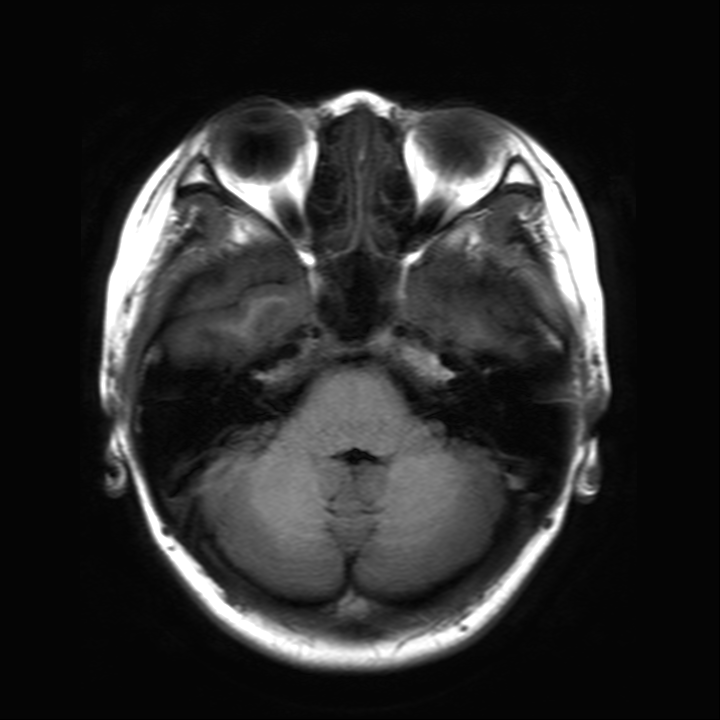

Supplement: Supplementary file 4 [file DataSheet4.zip › MRI-T1/T1-2.tif]

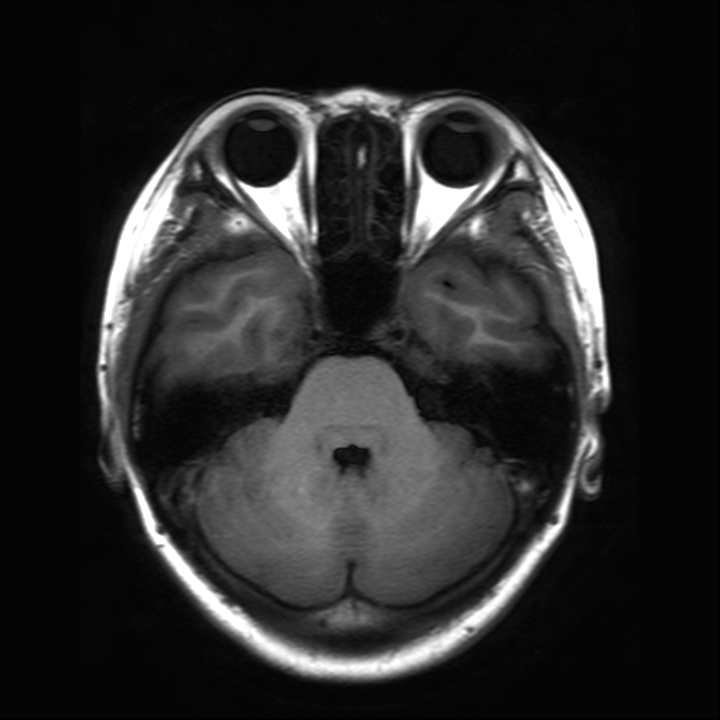

Supplement: Supplementary file 4 [file DataSheet4.zip › MRI-T1/T1-3.tif]

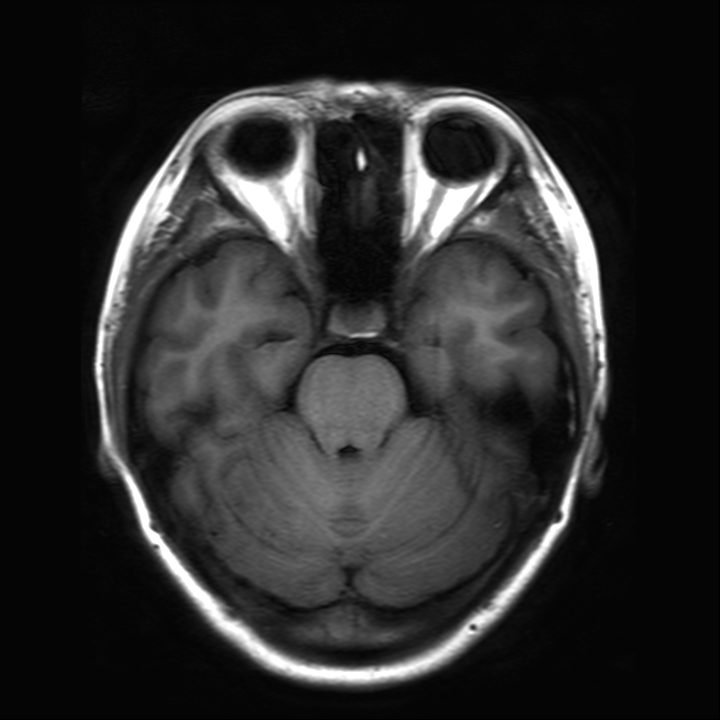

Supplement: Supplementary file 4 [file DataSheet4.zip › MRI-T1/T1-4.tif]

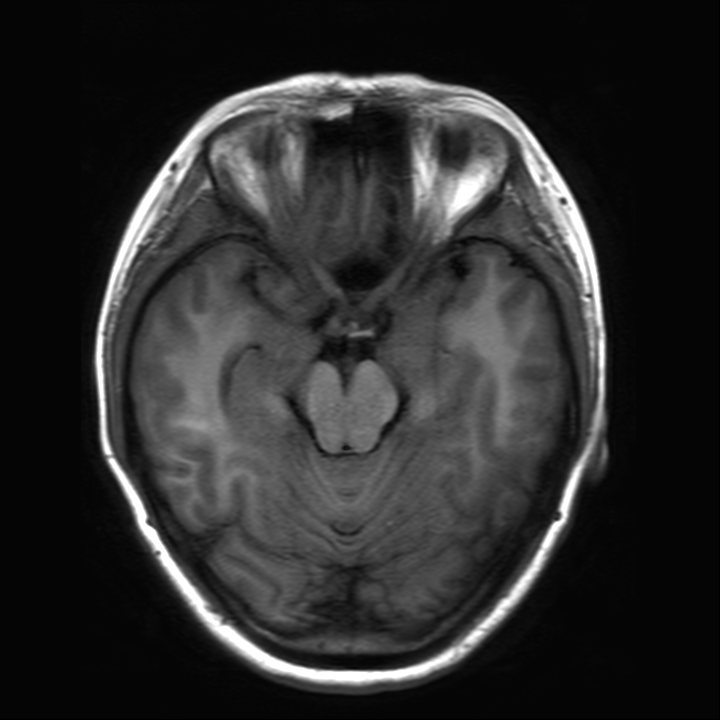

Supplement: Supplementary file 4 [file DataSheet4.zip › MRI-T1/T1-5.tif]

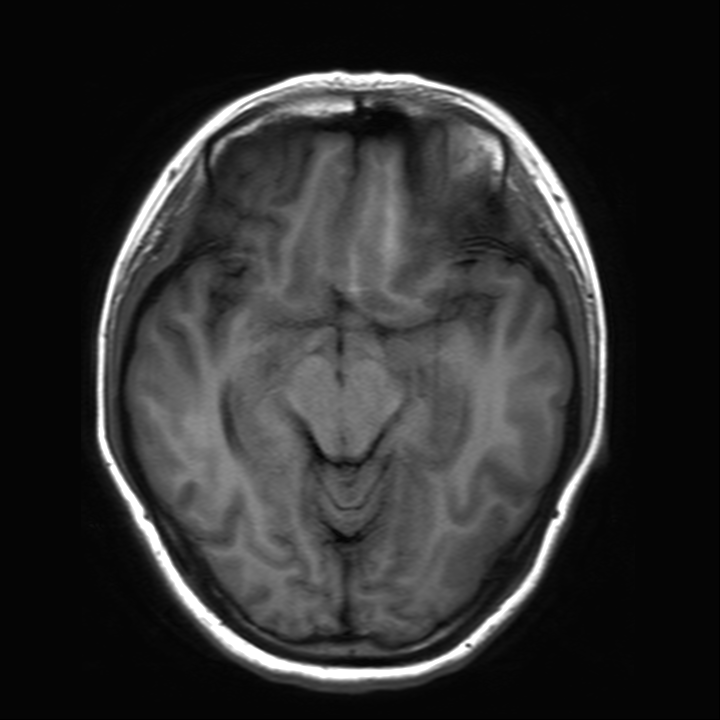

Supplement: Supplementary file 4 [file DataSheet4.zip › MRI-T1/T1-6.tif]

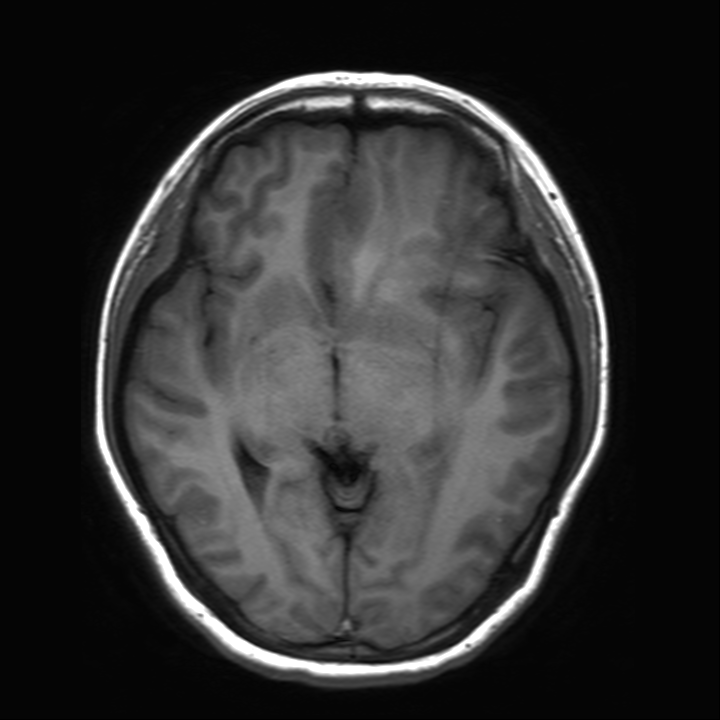

Supplement: Supplementary file 4 [file DataSheet4.zip › MRI-T1/T1-7.tif]

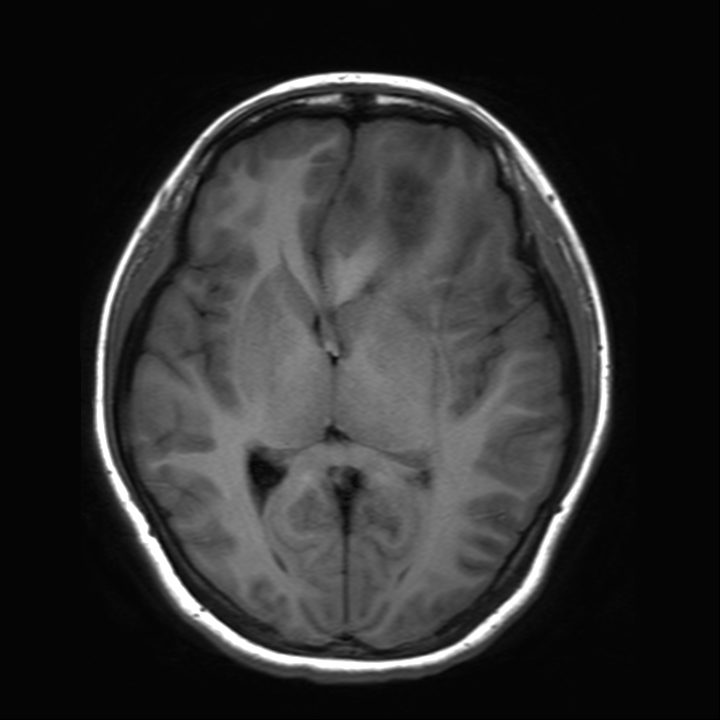

Supplement: Supplementary file 4 [file DataSheet4.zip › MRI-T1/T1-8.tif]

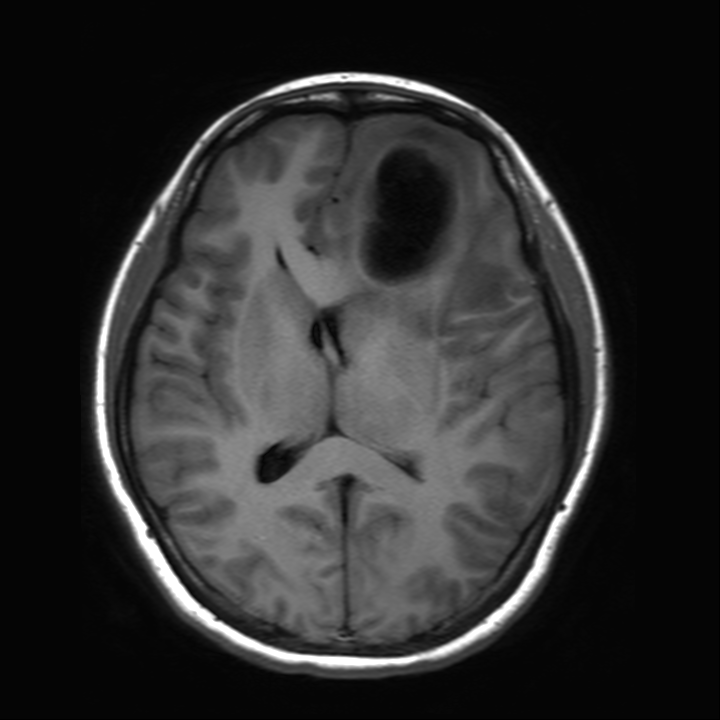

Supplement: Supplementary file 4 [file DataSheet4.zip › MRI-T1/T1-9.tif]

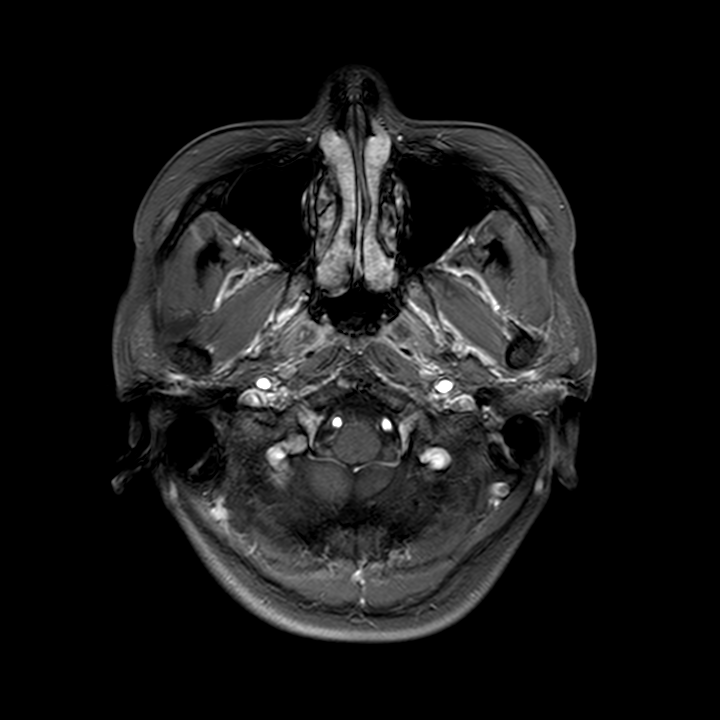

Supplement: Supplementary file 5 [file DataSheet5.zip › MRI-T1CE/T1CE-0.tif]

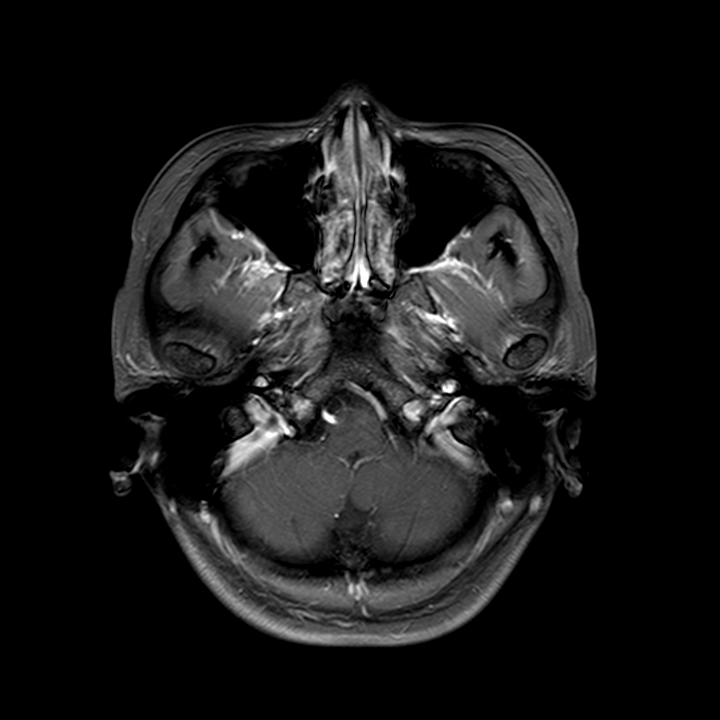

Supplement: Supplementary file 5 [file DataSheet5.zip › MRI-T1CE/T1CE-1.tif]

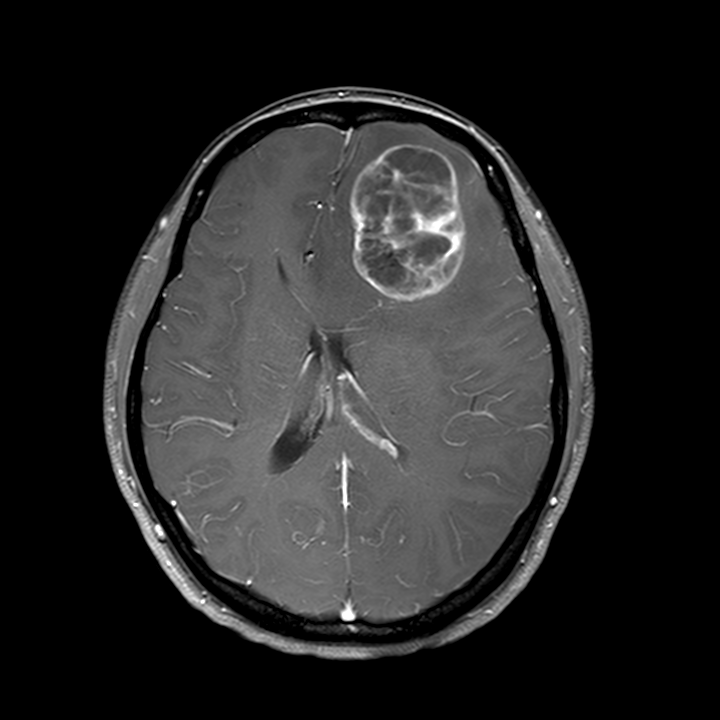

Supplement: Supplementary file 5 [file DataSheet5.zip › MRI-T1CE/T1CE-10.tif]

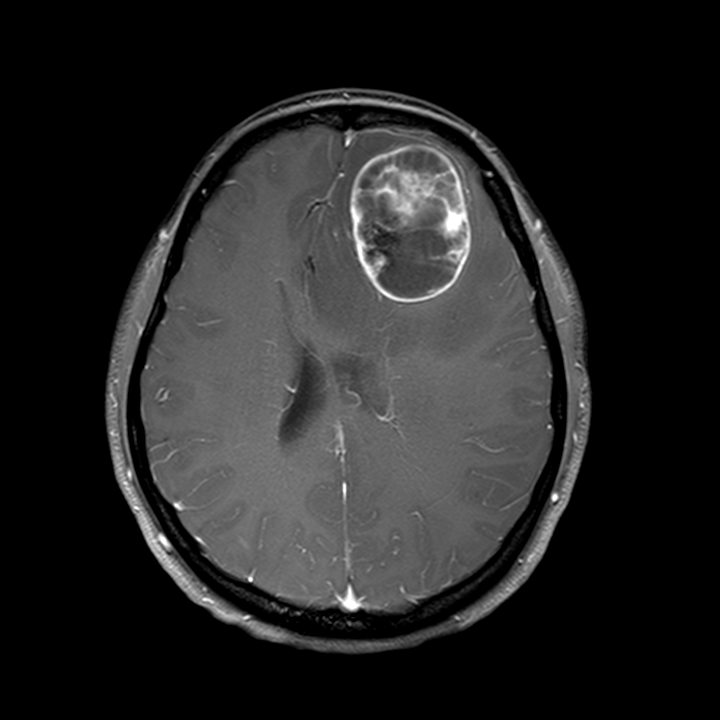

Supplement: Supplementary file 5 [file DataSheet5.zip › MRI-T1CE/T1CE-11.tif]

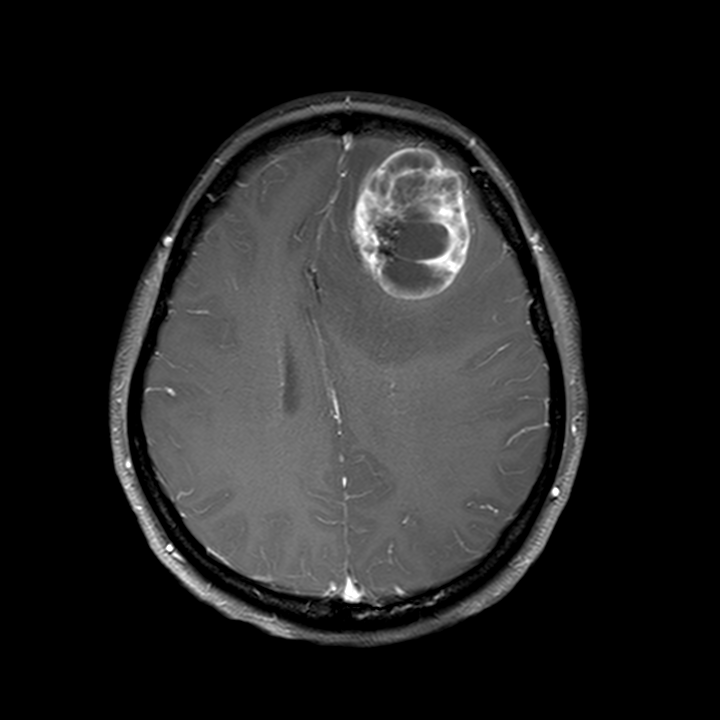

Supplement: Supplementary file 5 [file DataSheet5.zip › MRI-T1CE/T1CE-12.tif]

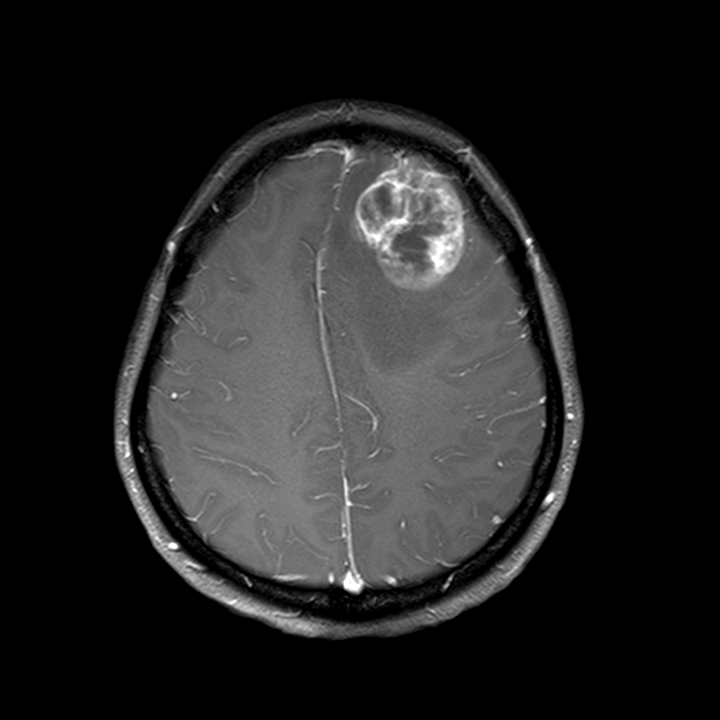

Supplement: Supplementary file 5 [file DataSheet5.zip › MRI-T1CE/T1CE-13.tif]

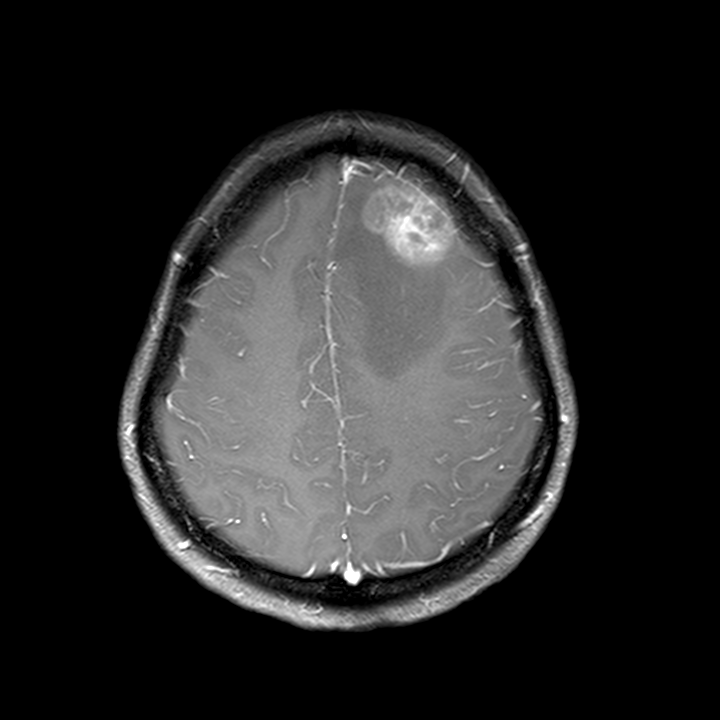

Supplement: Supplementary file 5 [file DataSheet5.zip › MRI-T1CE/T1CE-14.tif]

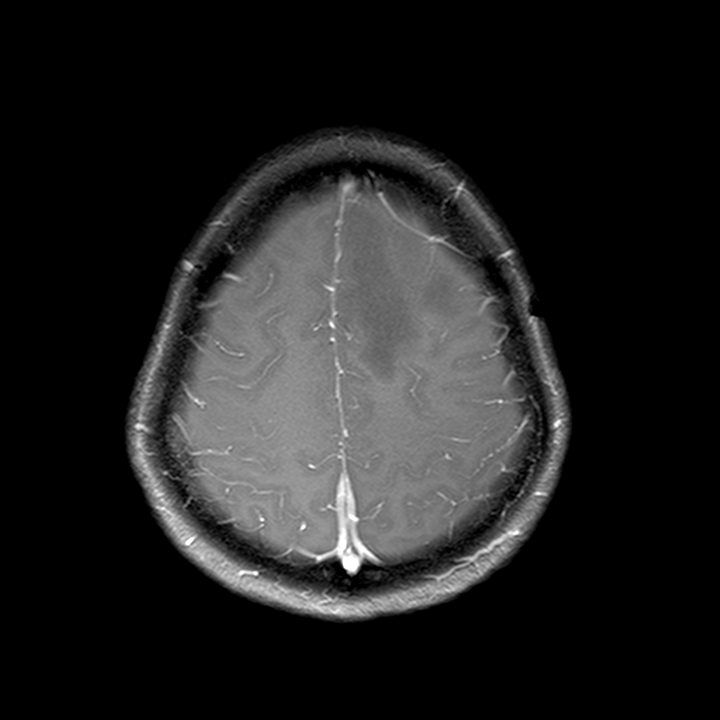

Supplement: Supplementary file 5 [file DataSheet5.zip › MRI-T1CE/T1CE-15.tif]

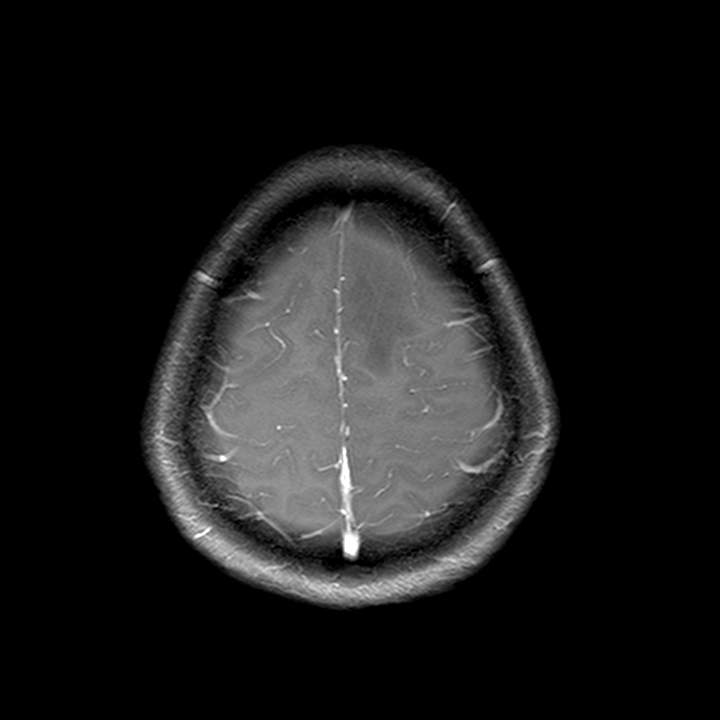

Supplement: Supplementary file 5 [file DataSheet5.zip › MRI-T1CE/T1CE-16.tif]

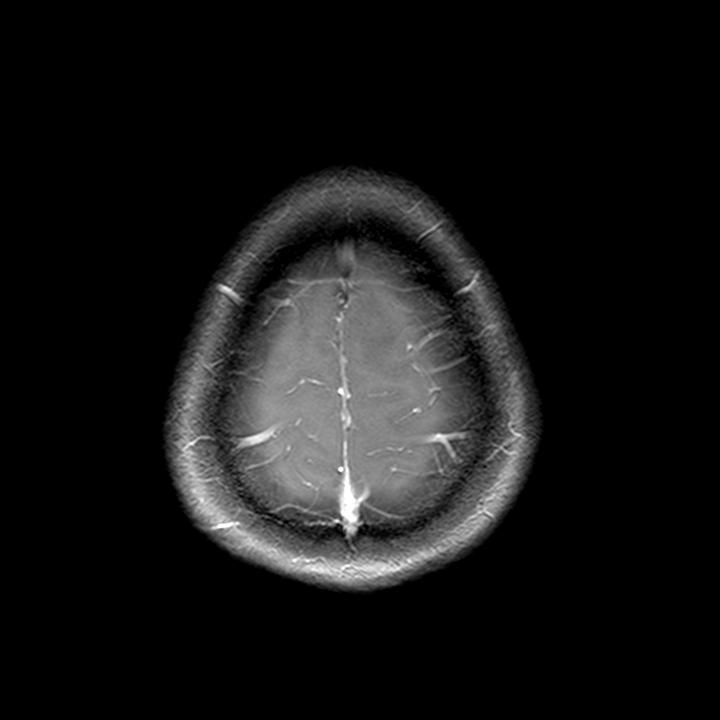

Supplement: Supplementary file 5 [file DataSheet5.zip › MRI-T1CE/T1CE-17.tif]

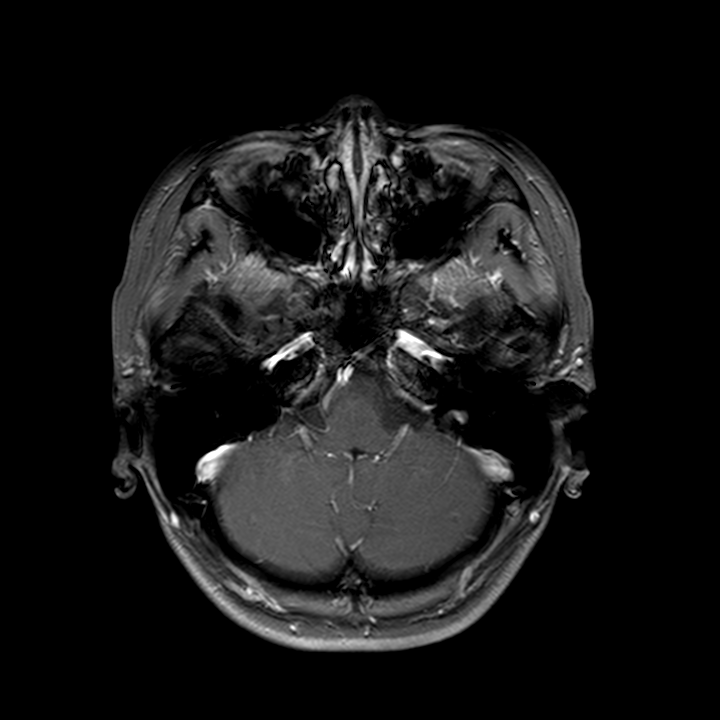

Supplement: Supplementary file 5 [file DataSheet5.zip › MRI-T1CE/T1CE-2.tif]

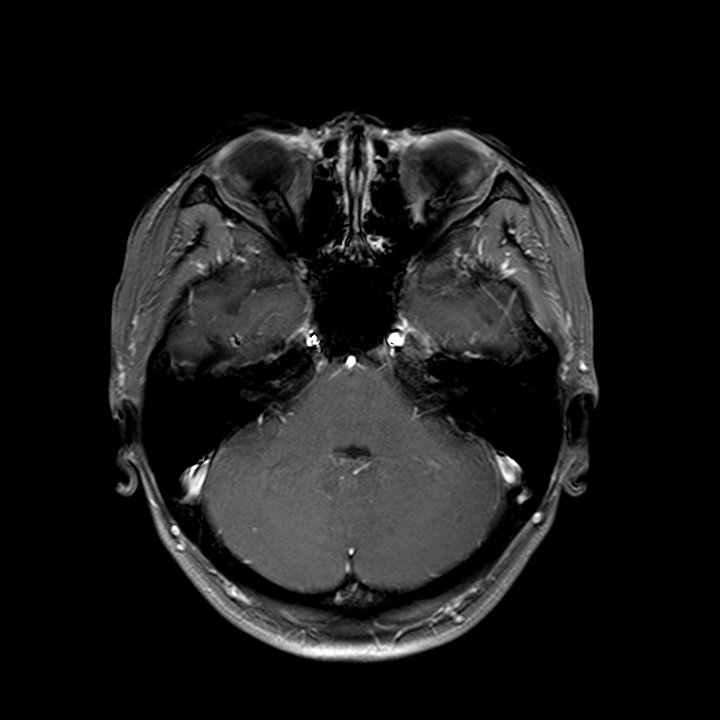

Supplement: Supplementary file 5 [file DataSheet5.zip › MRI-T1CE/T1CE-3.tif]

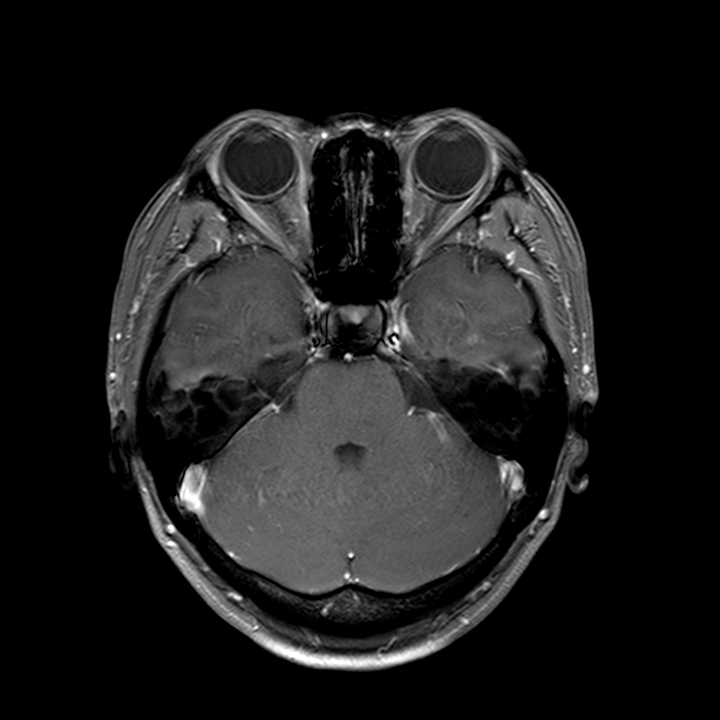

Supplement: Supplementary file 5 [file DataSheet5.zip › MRI-T1CE/T1CE-4.tif]

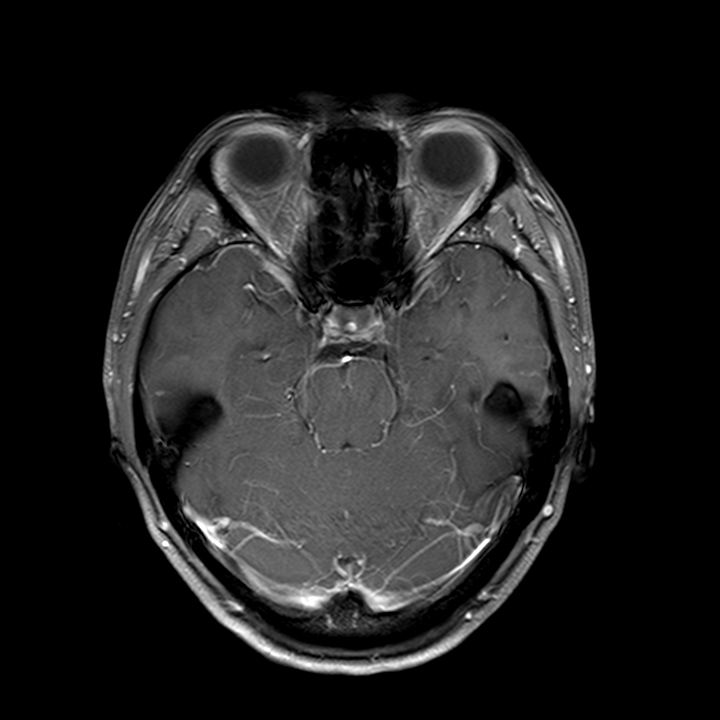

Supplement: Supplementary file 5 [file DataSheet5.zip › MRI-T1CE/T1CE-5.tif]

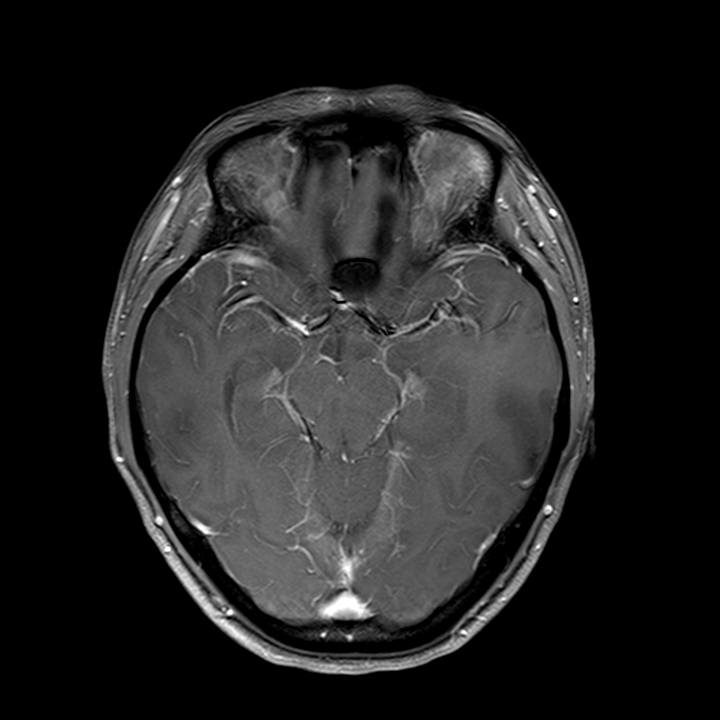

Supplement: Supplementary file 5 [file DataSheet5.zip › MRI-T1CE/T1CE-6.tif]

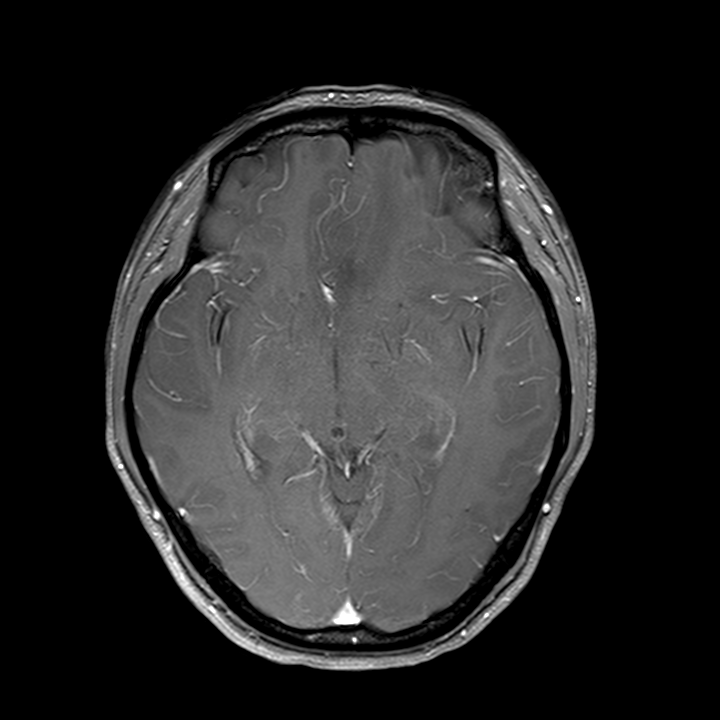

Supplement: Supplementary file 5 [file DataSheet5.zip › MRI-T1CE/T1CE-7.tif]

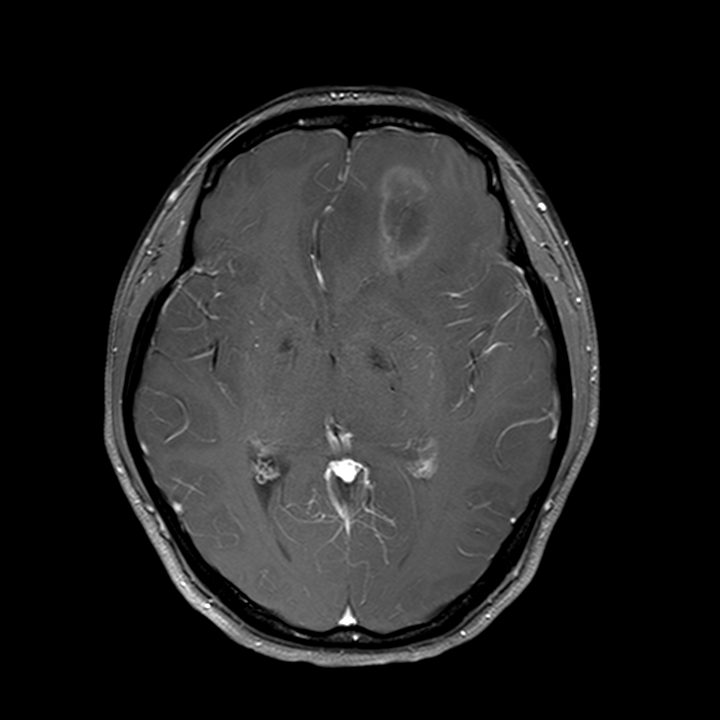

Supplement: Supplementary file 5 [file DataSheet5.zip › MRI-T1CE/T1CE-8.tif]

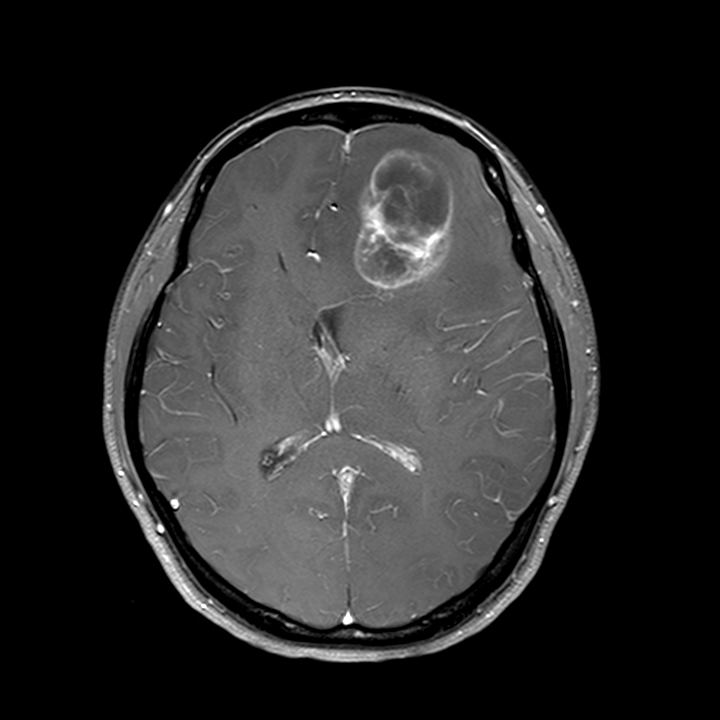

Supplement: Supplementary file 5 [file DataSheet5.zip › MRI-T1CE/T1CE-9.tif]

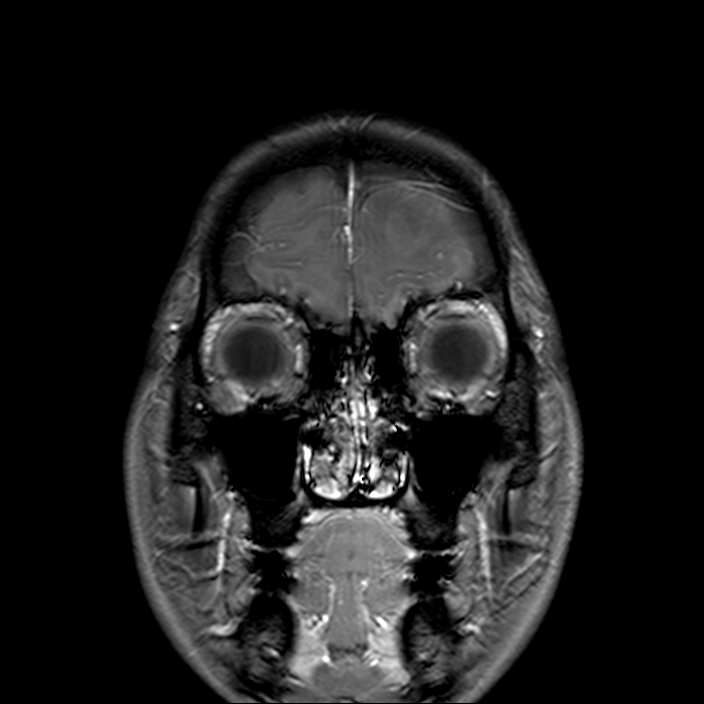

Supplement: Supplementary file 5 [file DataSheet5.zip › MRI-T1CE/T1CE-coronal0.tif]

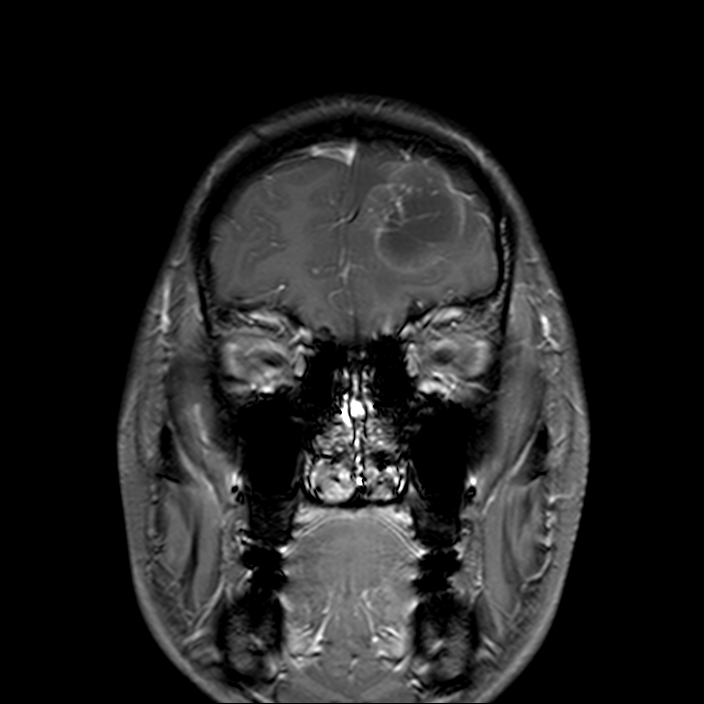

Supplement: Supplementary file 5 [file DataSheet5.zip › MRI-T1CE/T1CE-coronal1.tif]

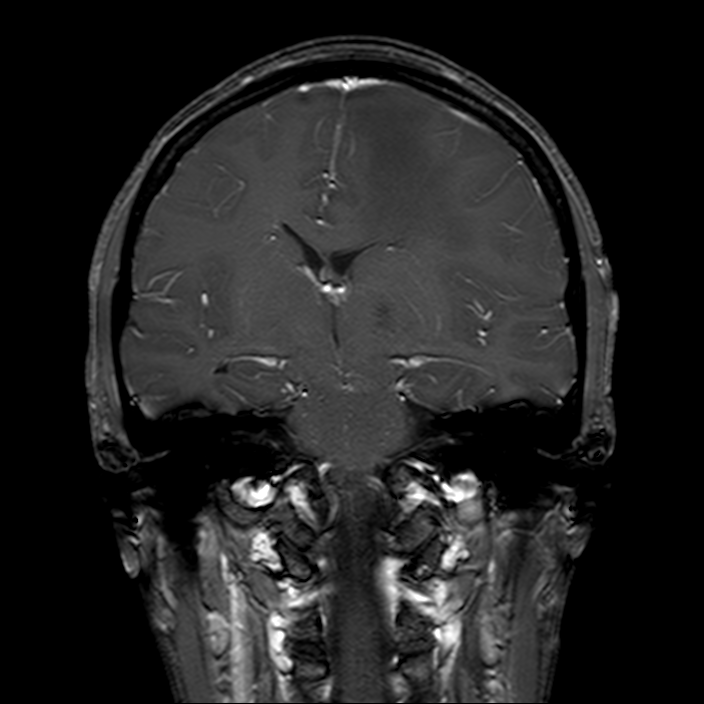

Supplement: Supplementary file 5 [file DataSheet5.zip › MRI-T1CE/T1CE-coronal10.tif]

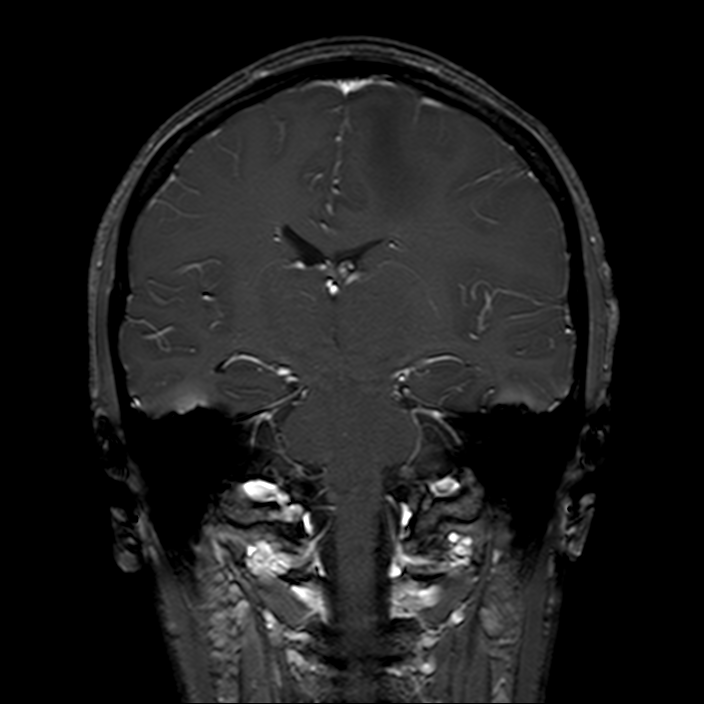

Supplement: Supplementary file 5 [file DataSheet5.zip › MRI-T1CE/T1CE-coronal11.tif]

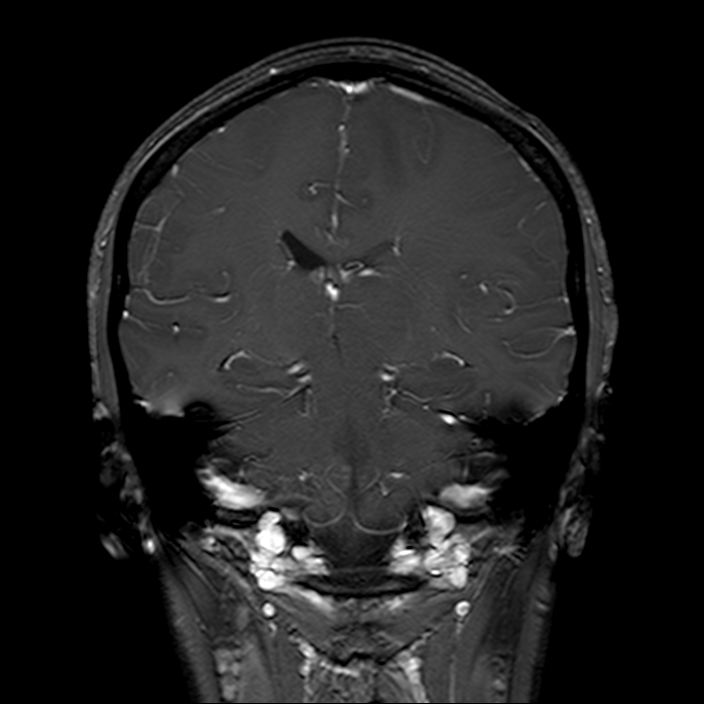

Supplement: Supplementary file 5 [file DataSheet5.zip › MRI-T1CE/T1CE-coronal12.tif]

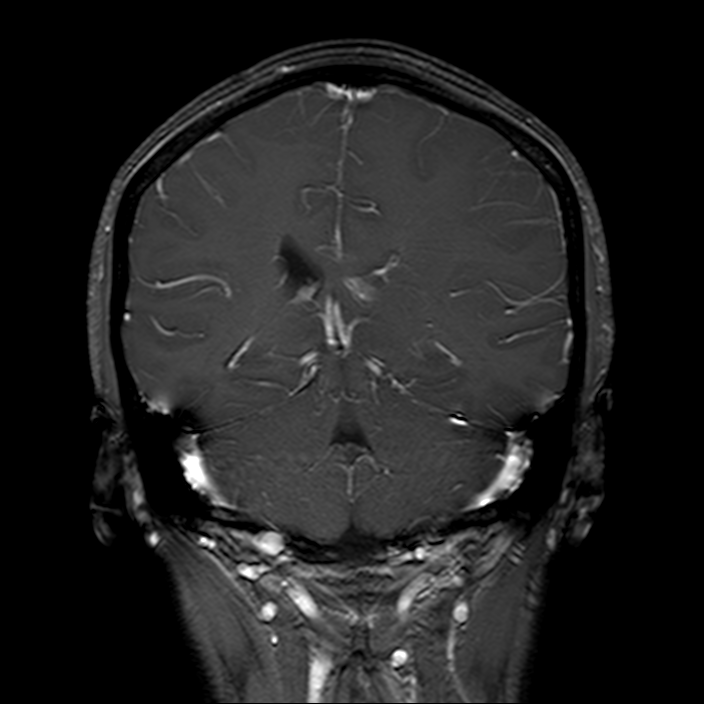

Supplement: Supplementary file 5 [file DataSheet5.zip › MRI-T1CE/T1CE-coronal13.tif]

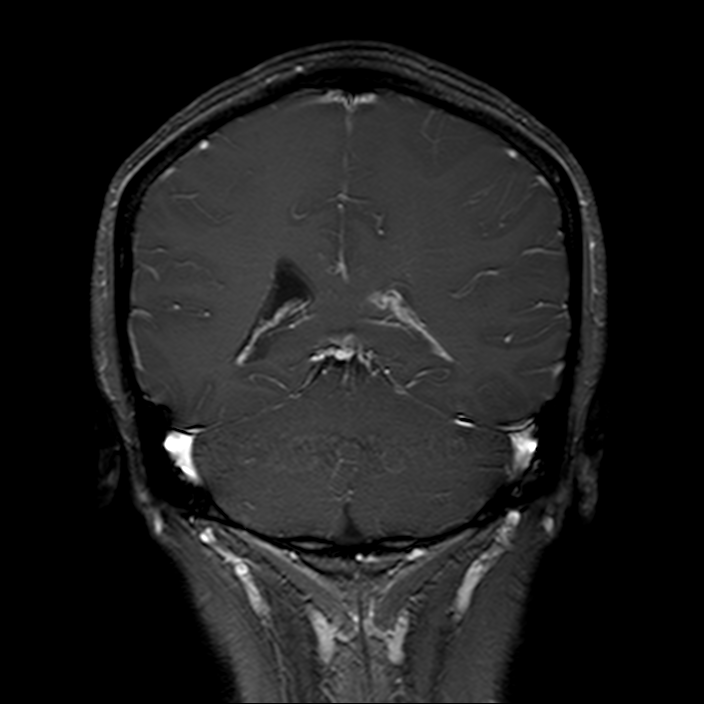

Supplement: Supplementary file 5 [file DataSheet5.zip › MRI-T1CE/T1CE-coronal14.tif]
