# Supplementary figures and images for: Primary intracranial myxopapillary ependymoma: two case reports and literature review (part 2 of 2)
Source: Front Oncol. 2026 May 21;16:1763325. doi: 10.3389/fonc.2026.1763325 (PMC13233268; doi:10.3389/fonc.2026.1763325)

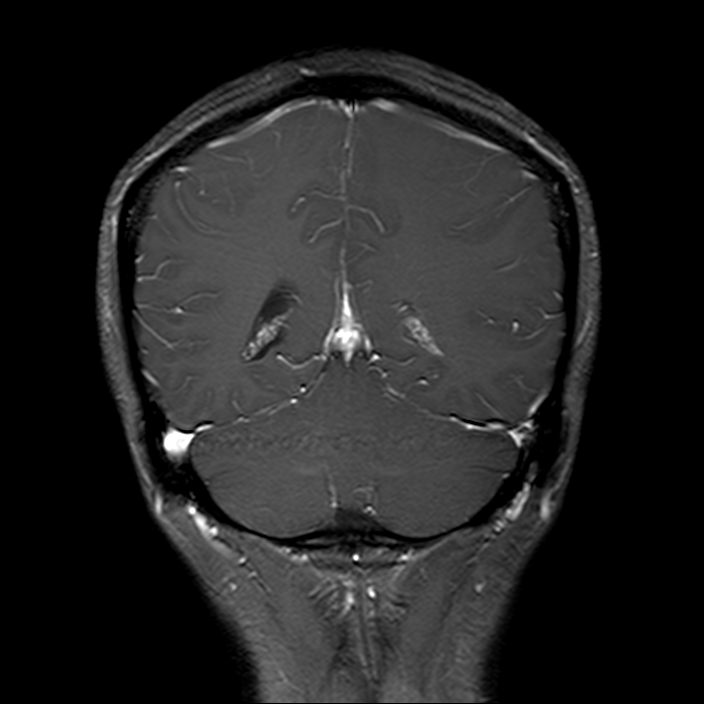

Supplement: Supplementary file 5 [file DataSheet5.zip › MRI-T1CE/T1CE-coronal15.tif]

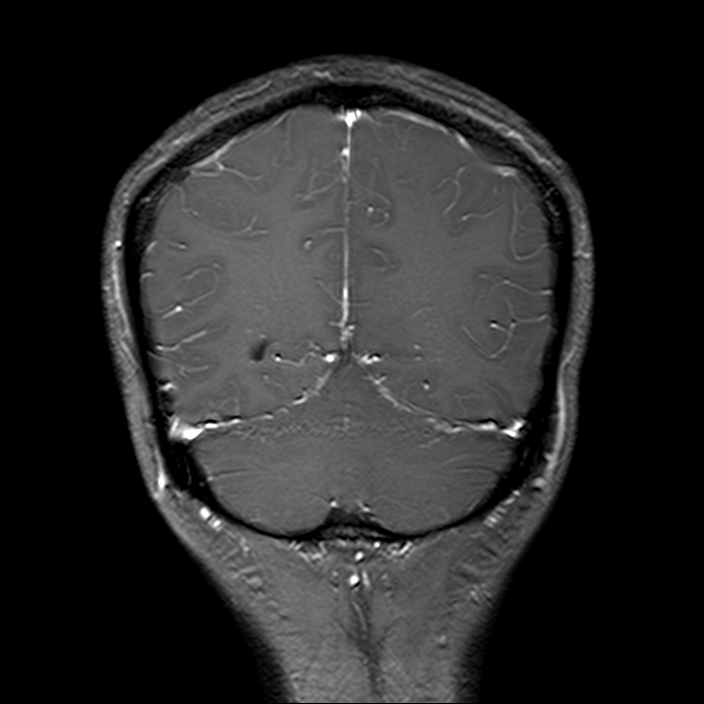

Supplement: Supplementary file 5 [file DataSheet5.zip › MRI-T1CE/T1CE-coronal16.tif]

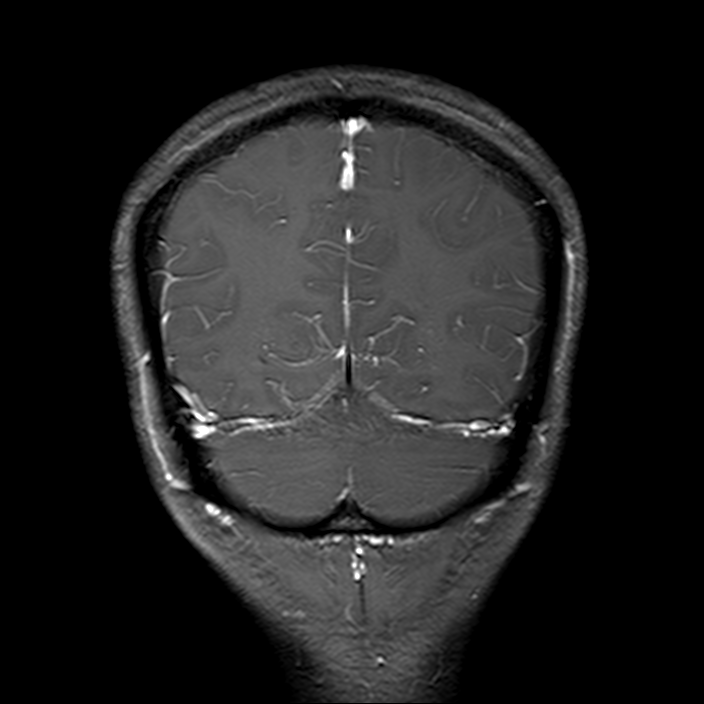

Supplement: Supplementary file 5 [file DataSheet5.zip › MRI-T1CE/T1CE-coronal17.tif]

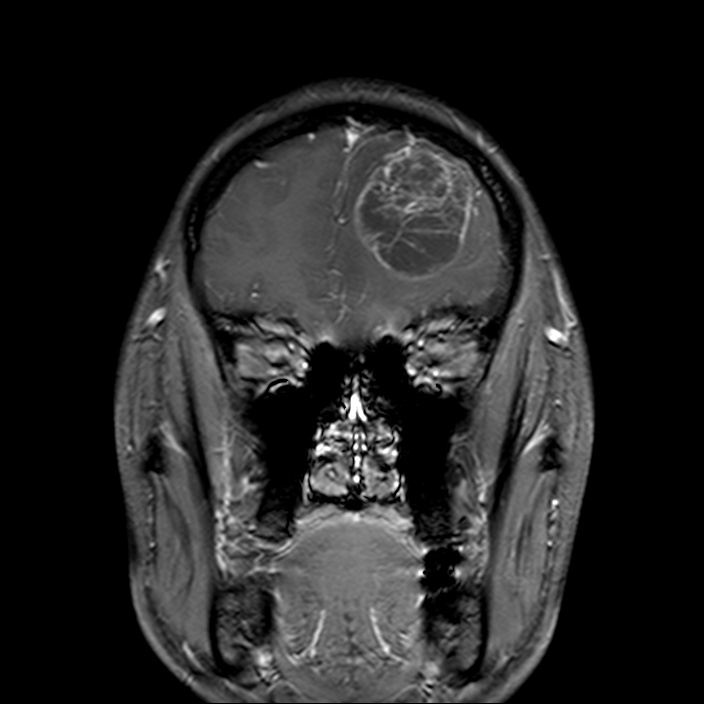

Supplement: Supplementary file 5 [file DataSheet5.zip › MRI-T1CE/T1CE-coronal2.tif]

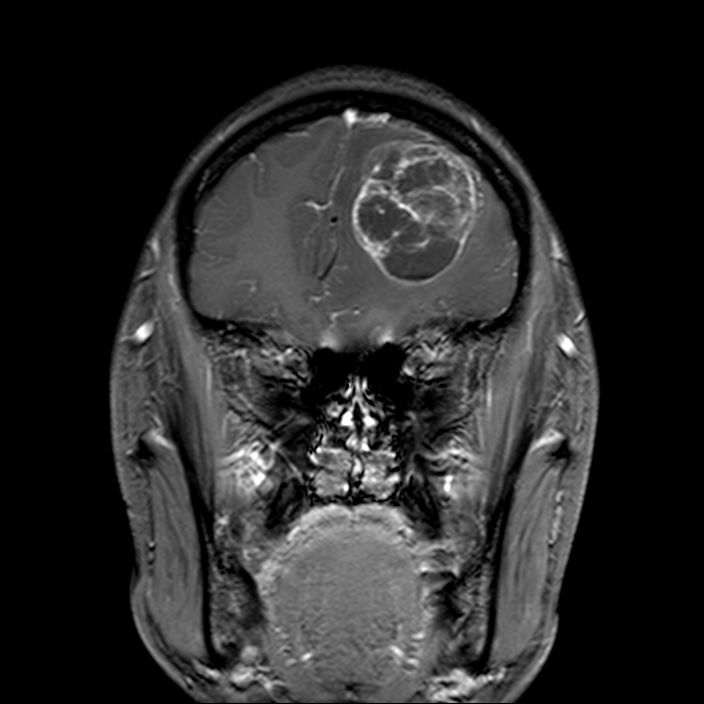

Supplement: Supplementary file 5 [file DataSheet5.zip › MRI-T1CE/T1CE-coronal3.tif]

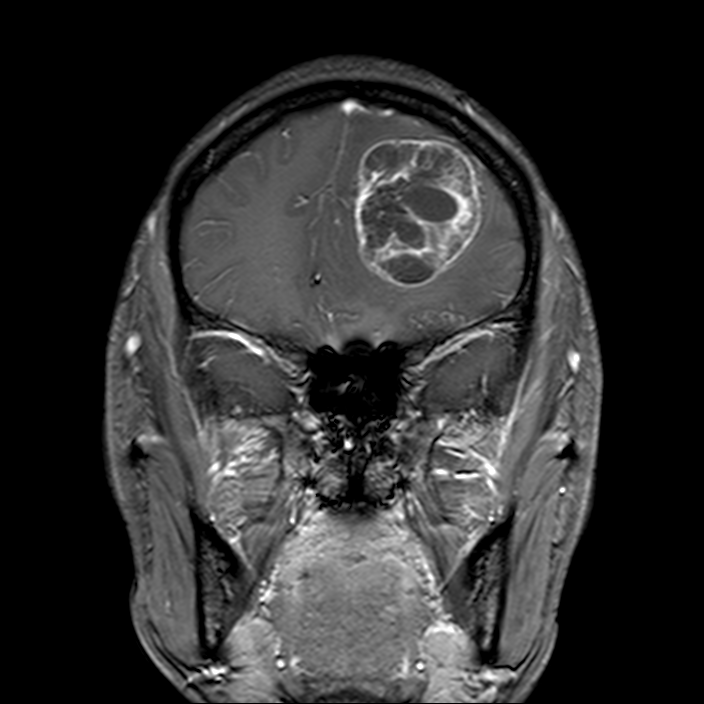

Supplement: Supplementary file 5 [file DataSheet5.zip › MRI-T1CE/T1CE-coronal4.tif]

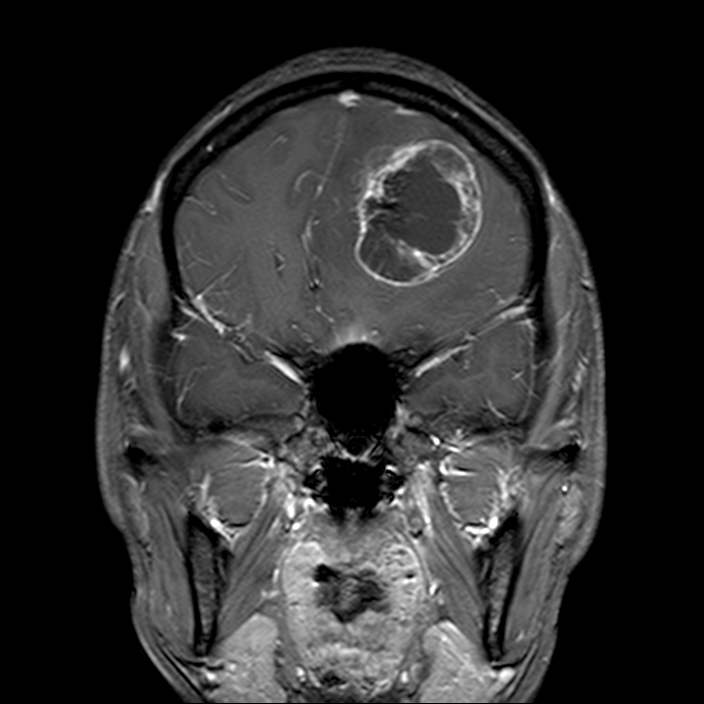

Supplement: Supplementary file 5 [file DataSheet5.zip › MRI-T1CE/T1CE-coronal5.tif]

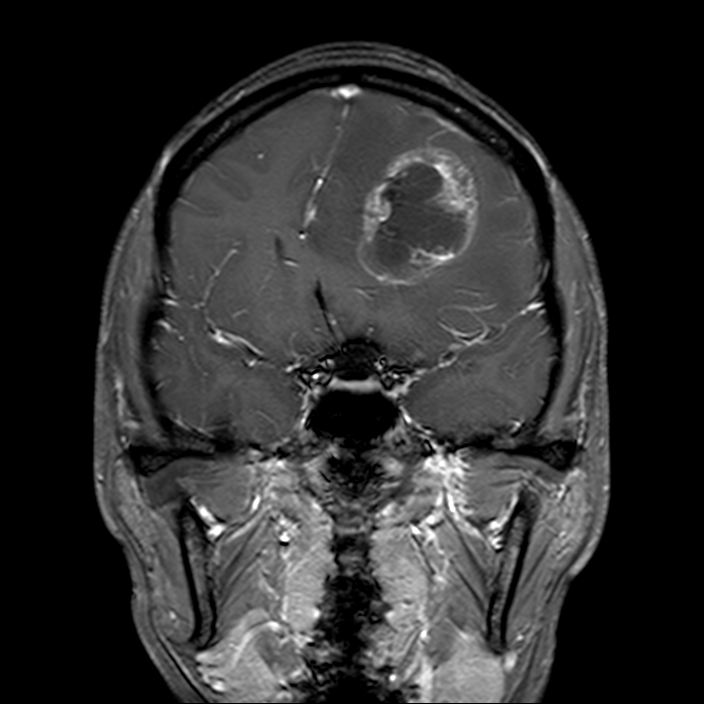

Supplement: Supplementary file 5 [file DataSheet5.zip › MRI-T1CE/T1CE-coronal6.tif]

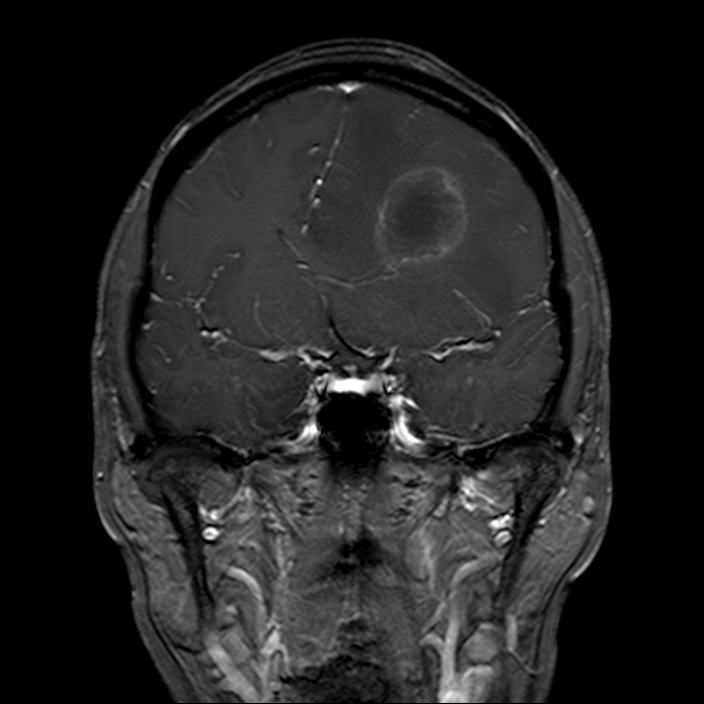

Supplement: Supplementary file 5 [file DataSheet5.zip › MRI-T1CE/T1CE-coronal7.tif]

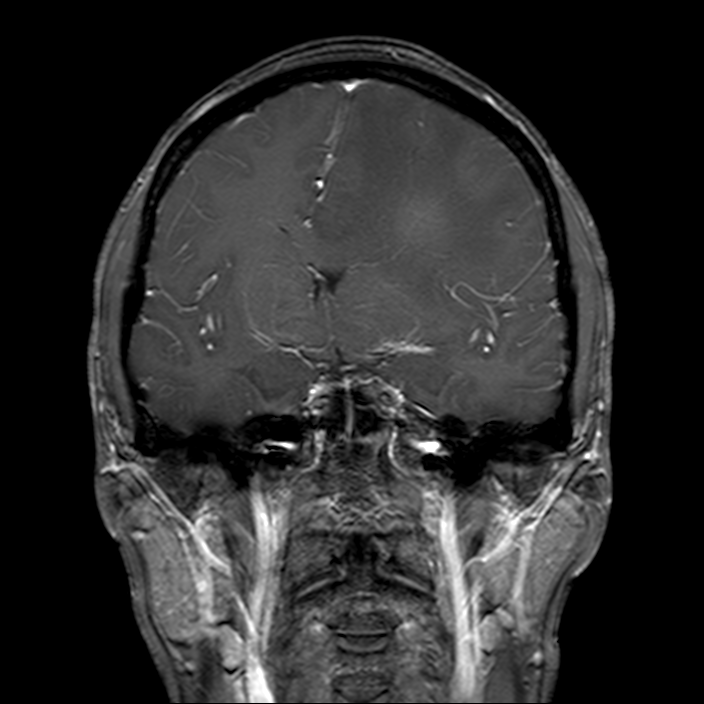

Supplement: Supplementary file 5 [file DataSheet5.zip › MRI-T1CE/T1CE-coronal8.tif]

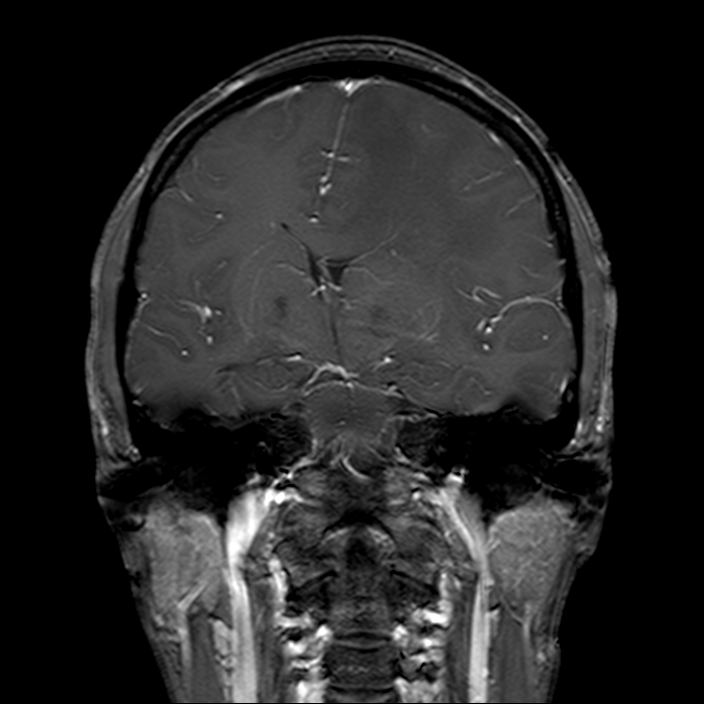

Supplement: Supplementary file 5 [file DataSheet5.zip › MRI-T1CE/T1CE-coronal9.tif]

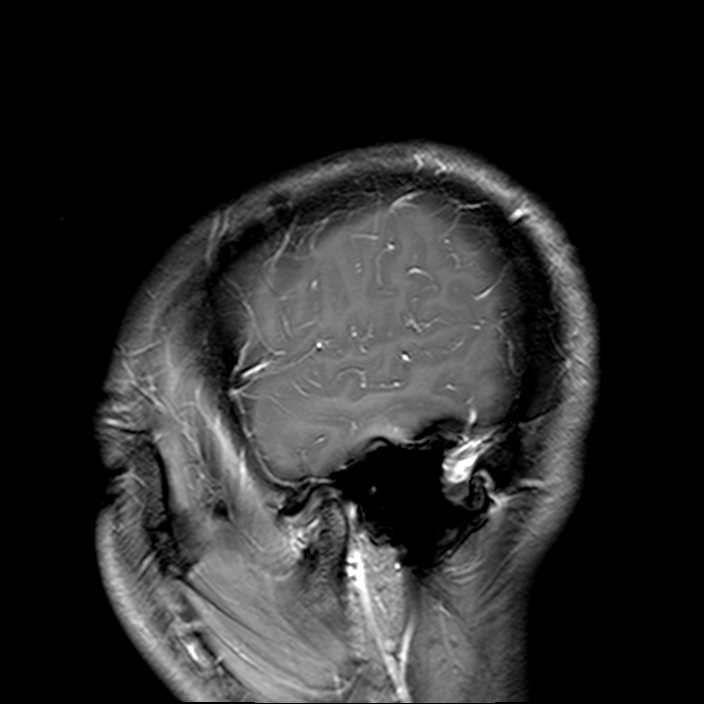

Supplement: Supplementary file 5 [file DataSheet5.zip › MRI-T1CE/T1CE-sagittal0.tif]

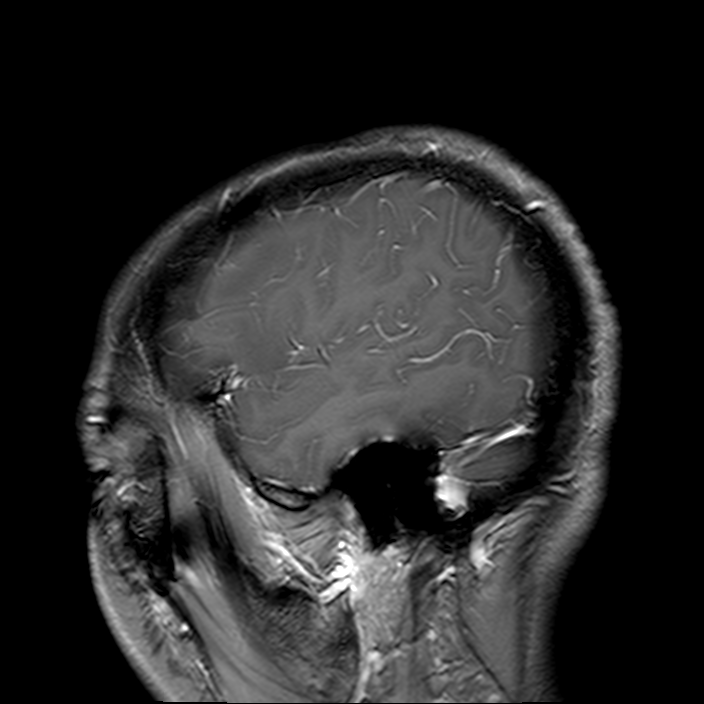

Supplement: Supplementary file 5 [file DataSheet5.zip › MRI-T1CE/T1CE-sagittal1.tif]

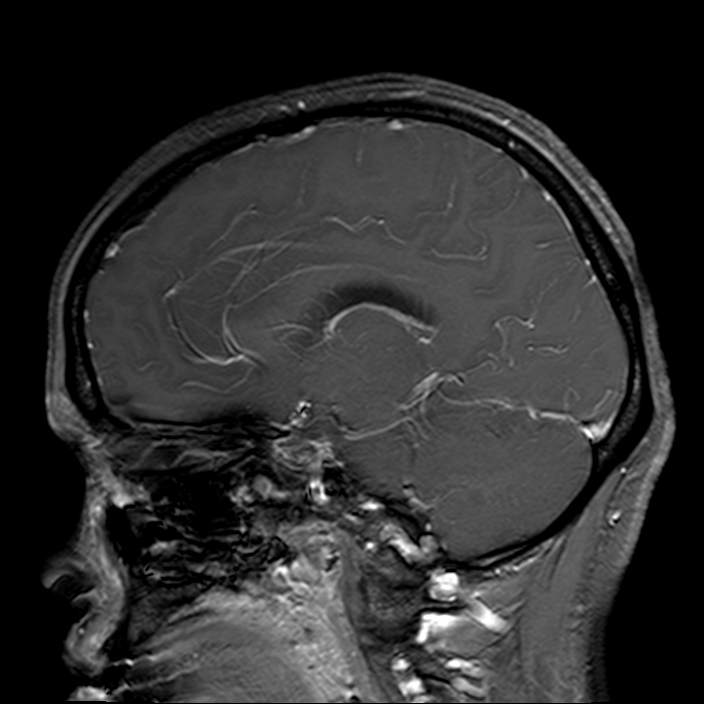

Supplement: Supplementary file 5 [file DataSheet5.zip › MRI-T1CE/T1CE-sagittal10.tif]

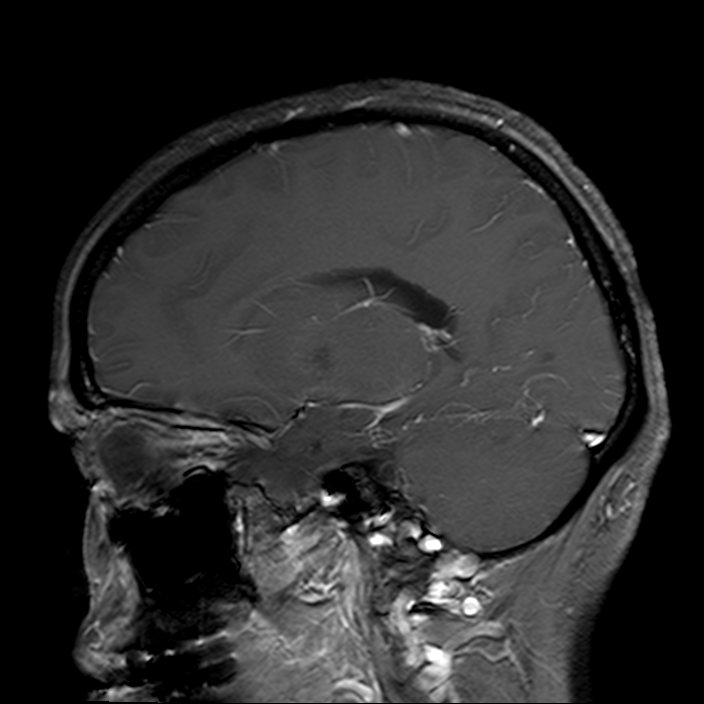

Supplement: Supplementary file 5 [file DataSheet5.zip › MRI-T1CE/T1CE-sagittal11.tif]

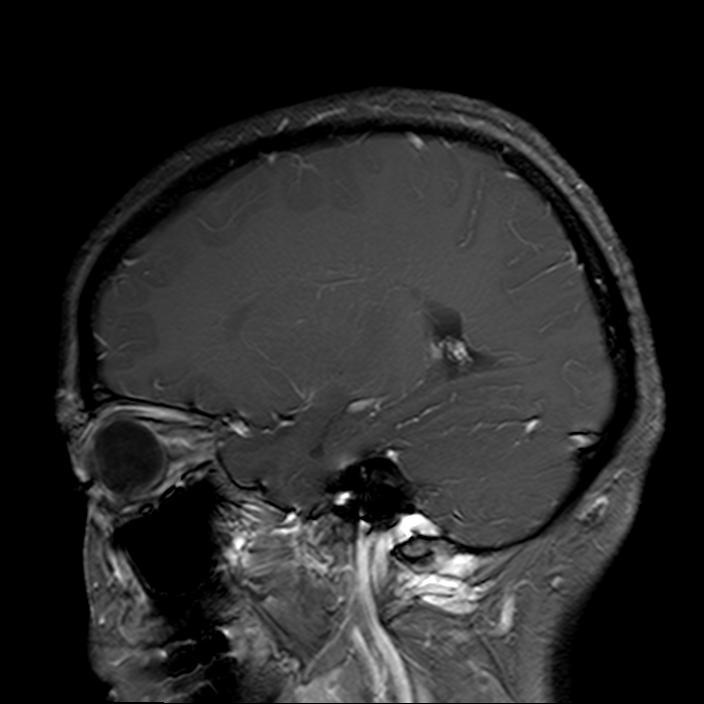

Supplement: Supplementary file 5 [file DataSheet5.zip › MRI-T1CE/T1CE-sagittal12.tif]

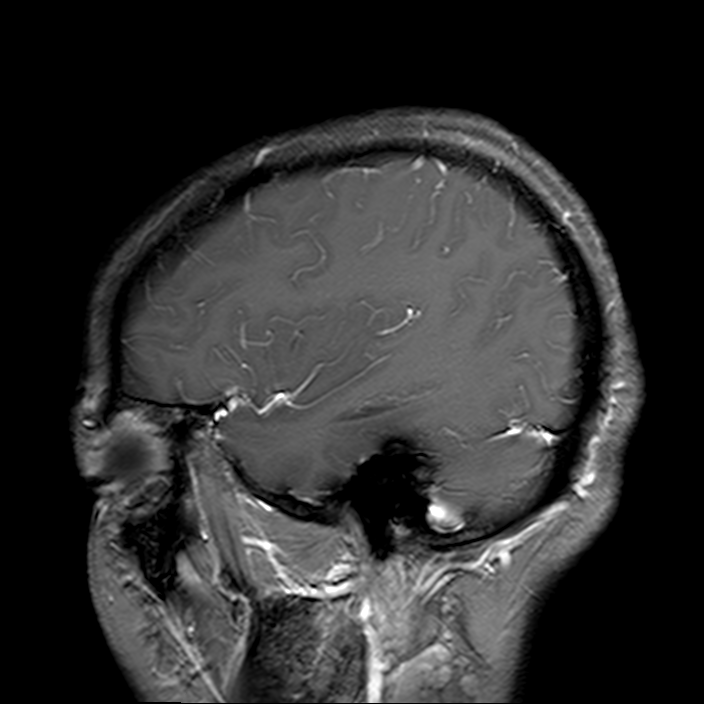

Supplement: Supplementary file 5 [file DataSheet5.zip › MRI-T1CE/T1CE-sagittal14.tif]

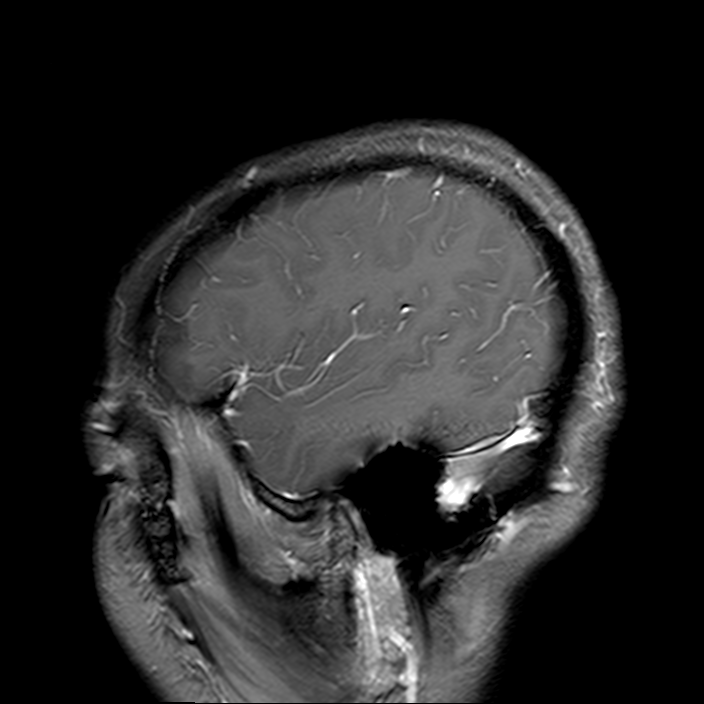

Supplement: Supplementary file 5 [file DataSheet5.zip › MRI-T1CE/T1CE-sagittal15.tif]

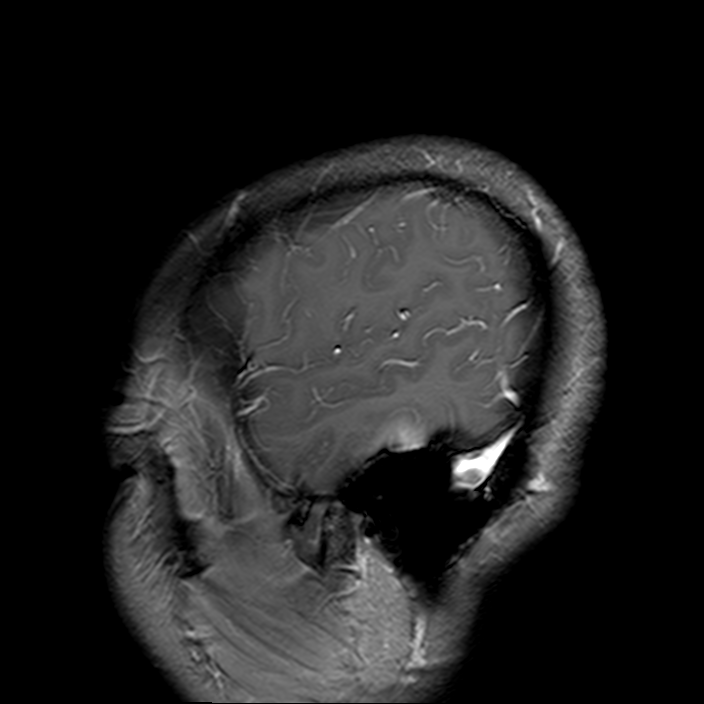

Supplement: Supplementary file 5 [file DataSheet5.zip › MRI-T1CE/T1CE-sagittal16.tif]

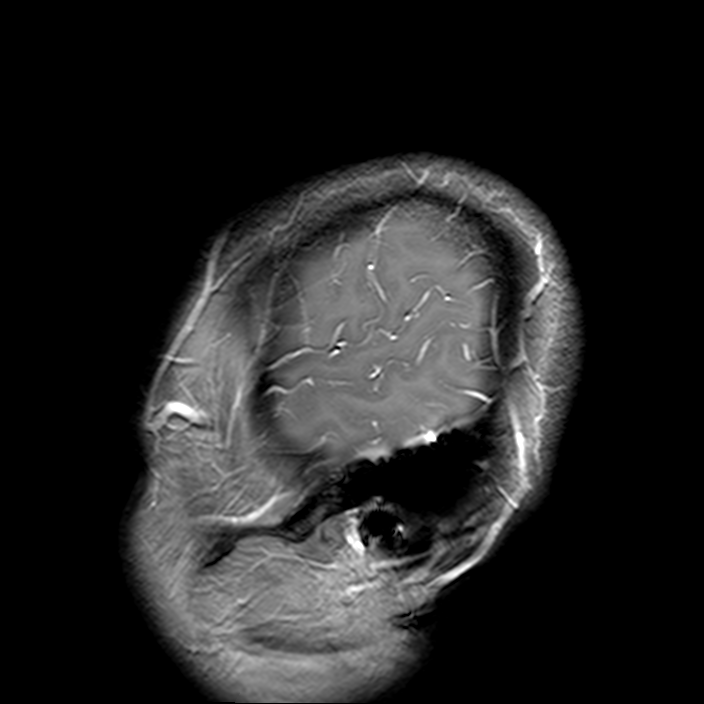

Supplement: Supplementary file 5 [file DataSheet5.zip › MRI-T1CE/T1CE-sagittal17.tif]

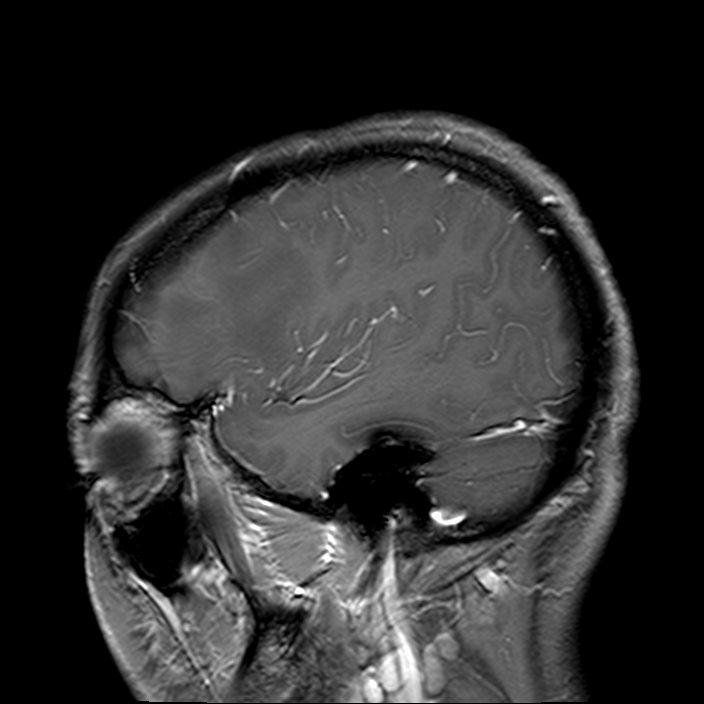

Supplement: Supplementary file 5 [file DataSheet5.zip › MRI-T1CE/T1CE-sagittal2.tif]

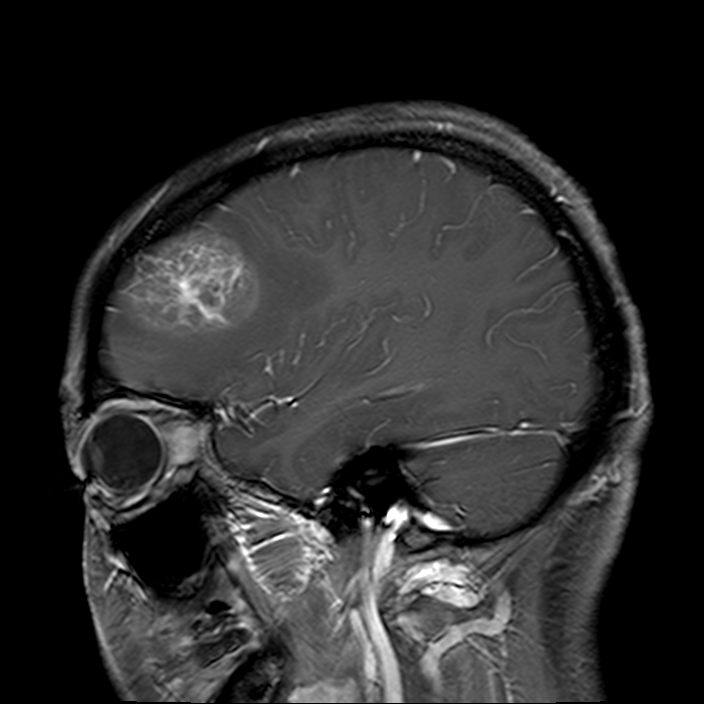

Supplement: Supplementary file 5 [file DataSheet5.zip › MRI-T1CE/T1CE-sagittal3.tif]

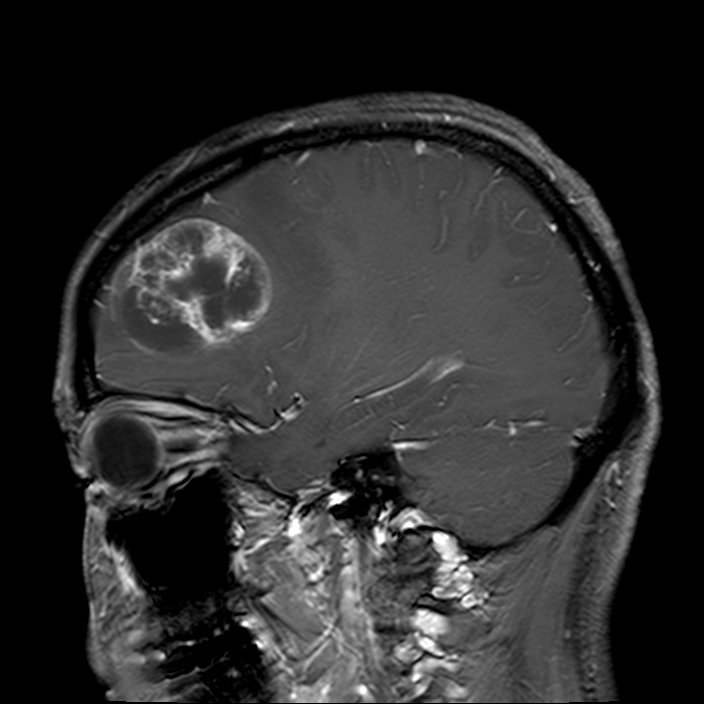

Supplement: Supplementary file 5 [file DataSheet5.zip › MRI-T1CE/T1CE-sagittal4.tif]

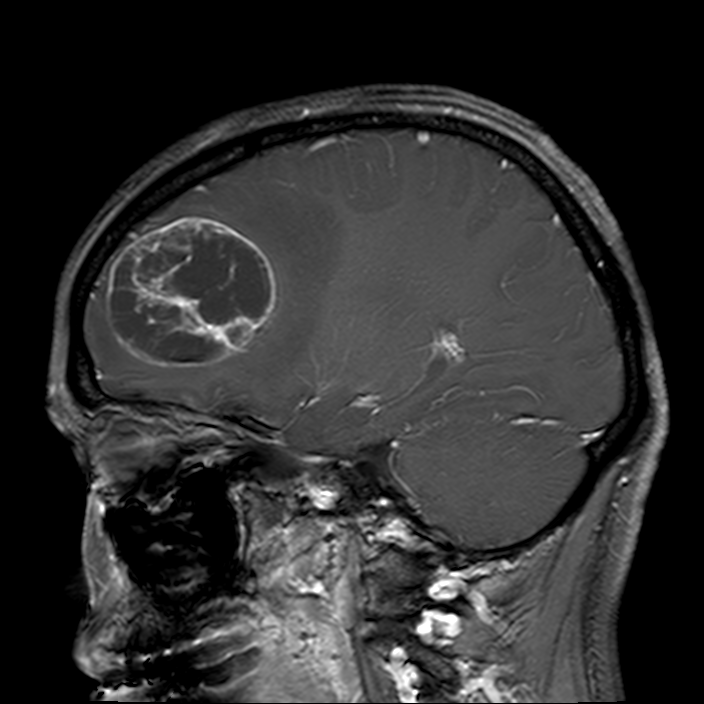

Supplement: Supplementary file 5 [file DataSheet5.zip › MRI-T1CE/T1CE-sagittal5.tif]

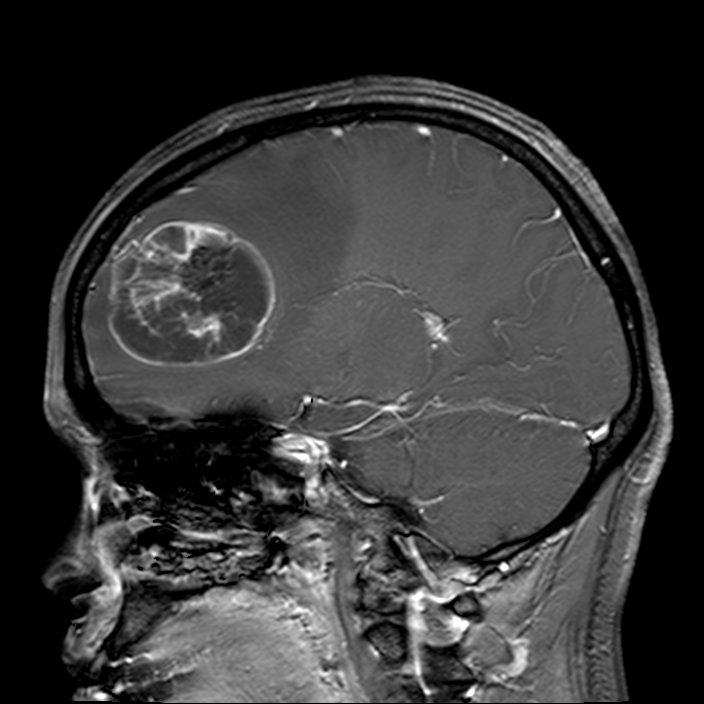

Supplement: Supplementary file 5 [file DataSheet5.zip › MRI-T1CE/T1CE-sagittal6.tif]

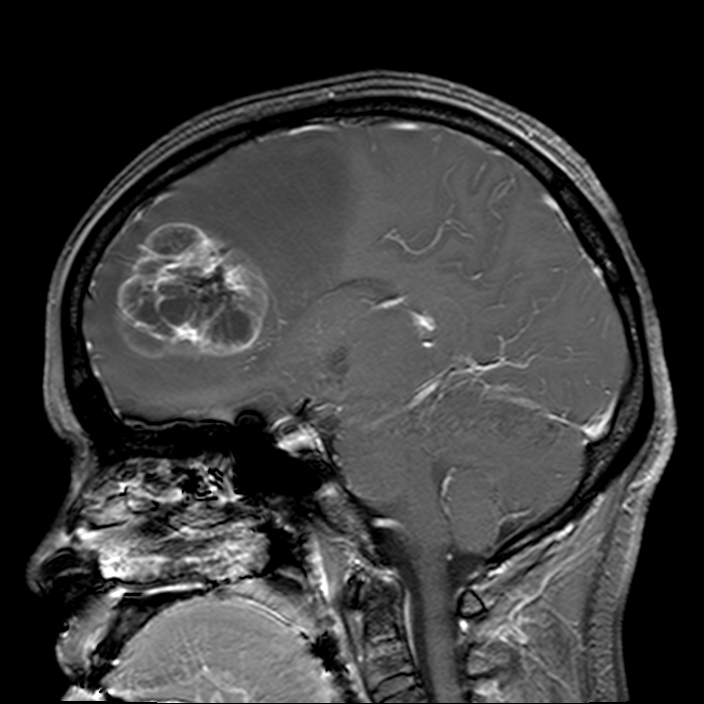

Supplement: Supplementary file 5 [file DataSheet5.zip › MRI-T1CE/T1CE-sagittal7.tif]

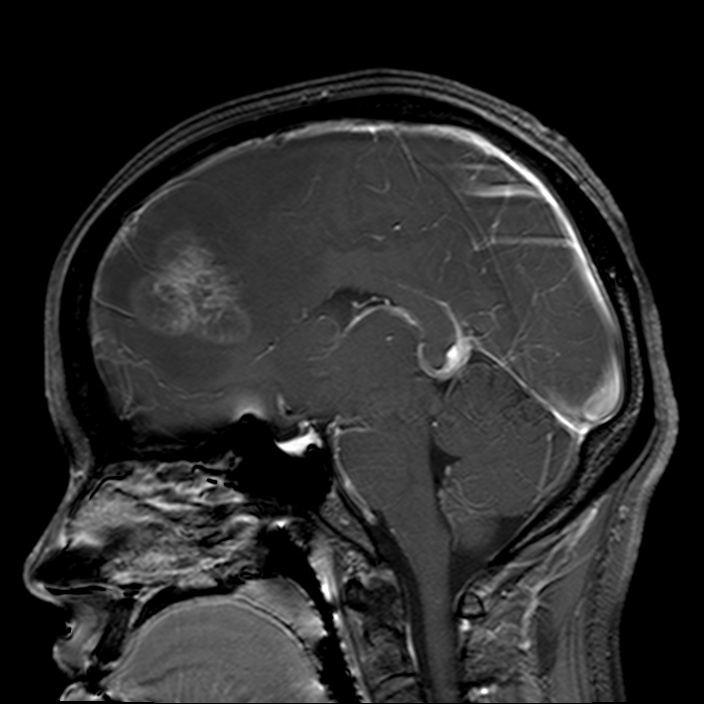

Supplement: Supplementary file 5 [file DataSheet5.zip › MRI-T1CE/T1CE-sagittal8.tif]

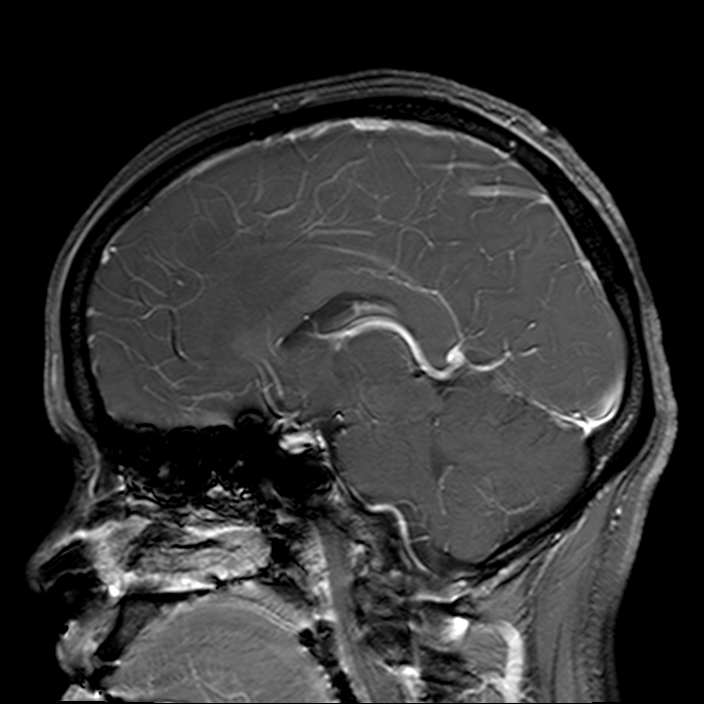

Supplement: Supplementary file 5 [file DataSheet5.zip › MRI-T1CE/T1CE-sagittal9.tif]

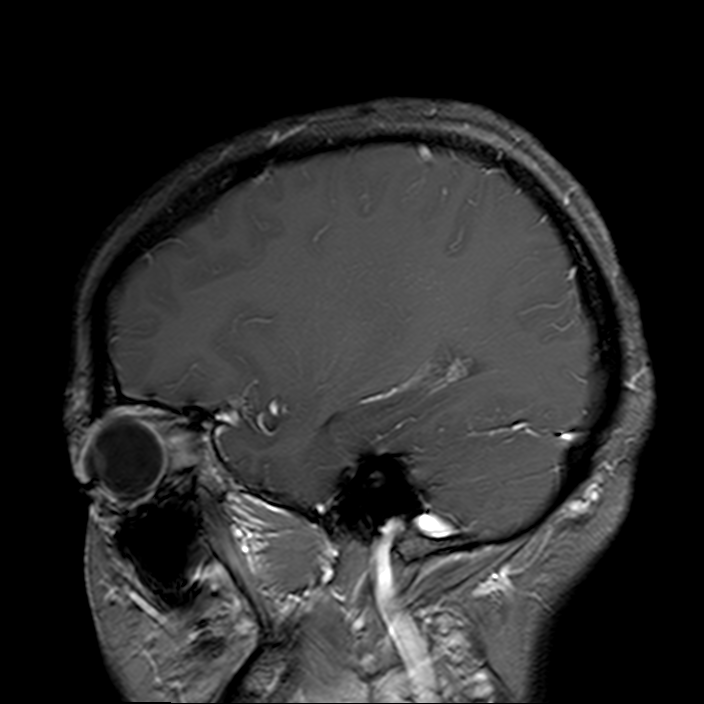

Supplement: Supplementary file 5 [file DataSheet5.zip › MRI-T1CE/T1CEsagittal13.tif]

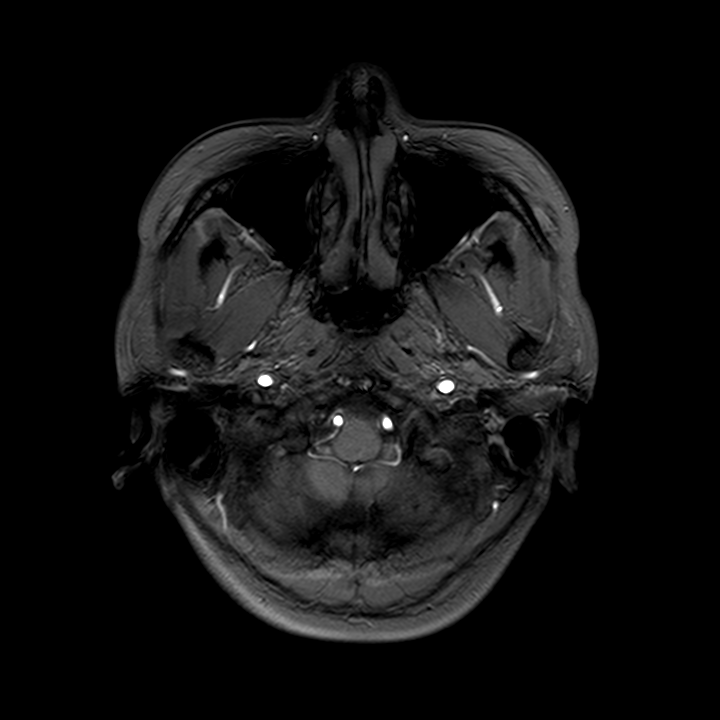

Supplement: Supplementary file 6 [file DataSheet6.zip › MRI-T1-FFE/T1-FFE-0.tif]

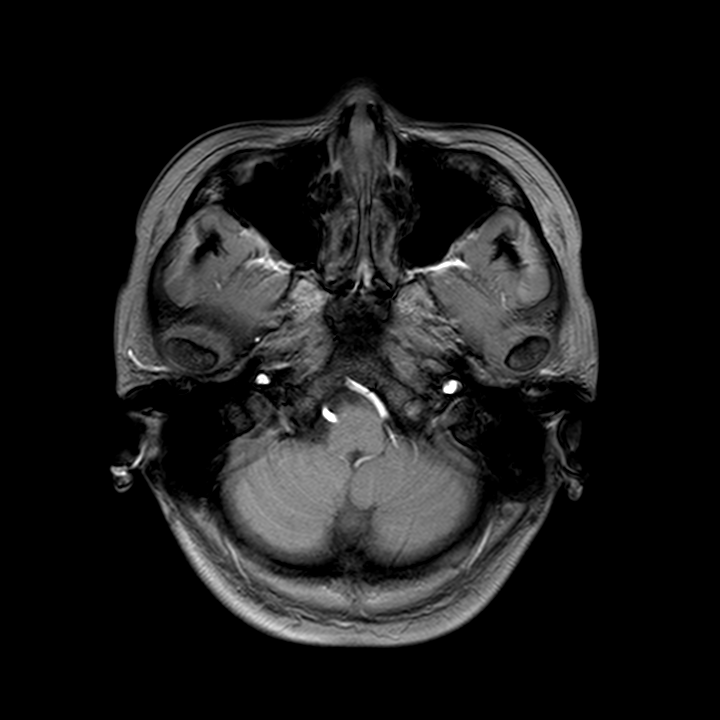

Supplement: Supplementary file 6 [file DataSheet6.zip › MRI-T1-FFE/T1-FFE-1.tif]

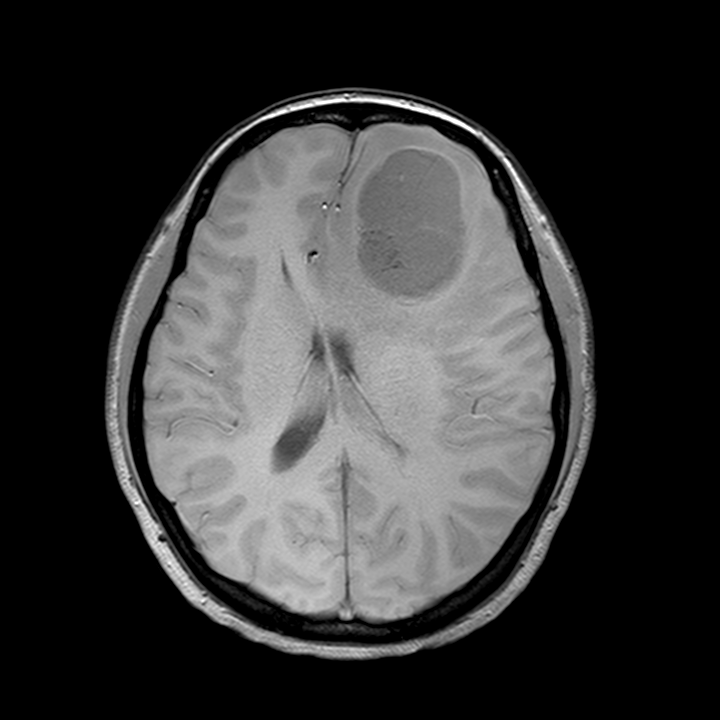

Supplement: Supplementary file 6 [file DataSheet6.zip › MRI-T1-FFE/T1-FFE-10.tif]

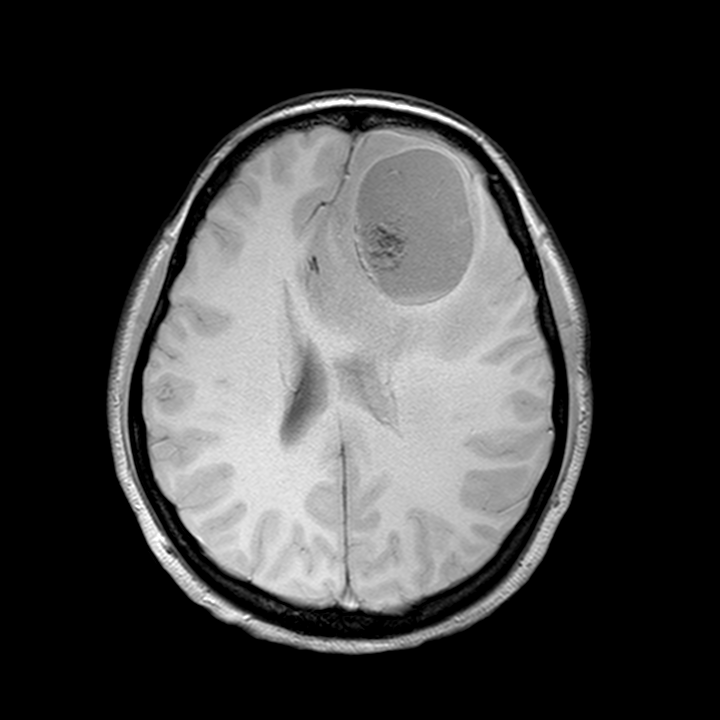

Supplement: Supplementary file 6 [file DataSheet6.zip › MRI-T1-FFE/T1-FFE-11.tif]

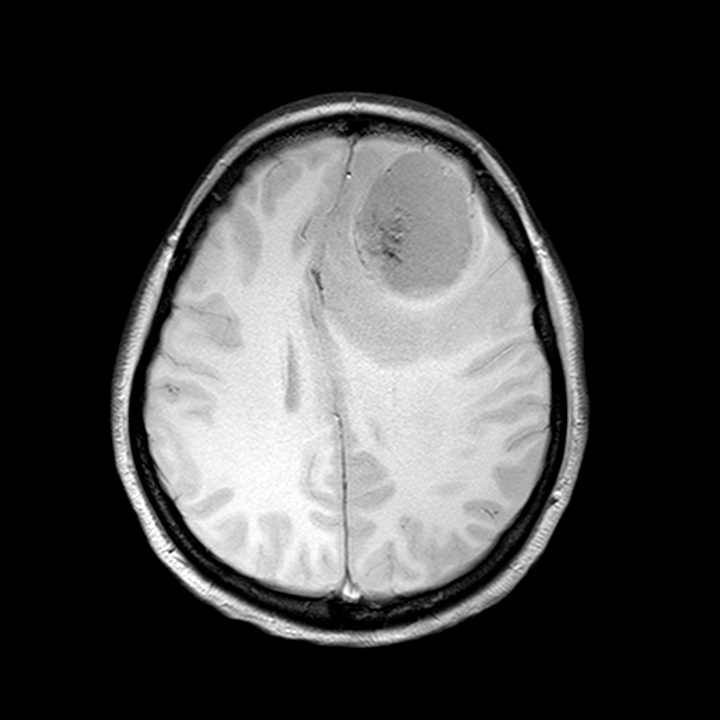

Supplement: Supplementary file 6 [file DataSheet6.zip › MRI-T1-FFE/T1-FFE-12.tif]

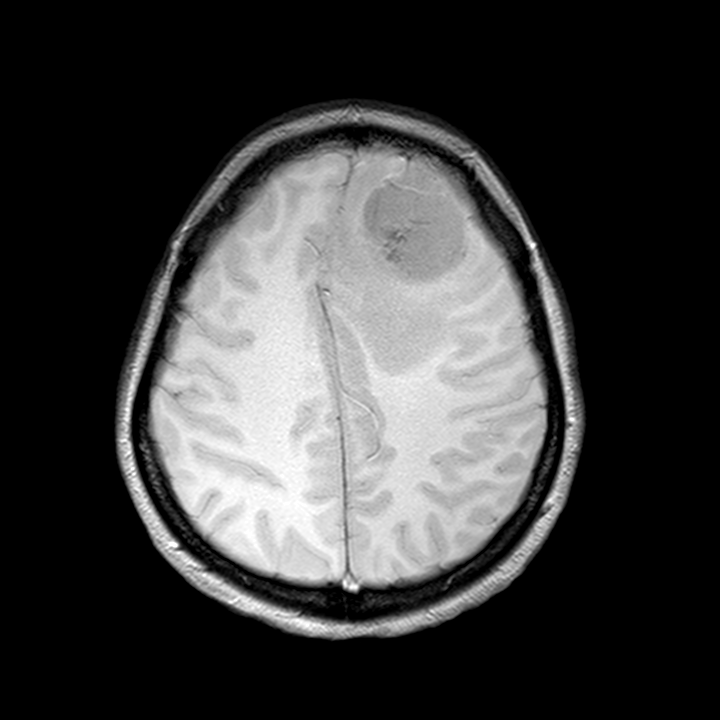

Supplement: Supplementary file 6 [file DataSheet6.zip › MRI-T1-FFE/T1-FFE-13.tif]

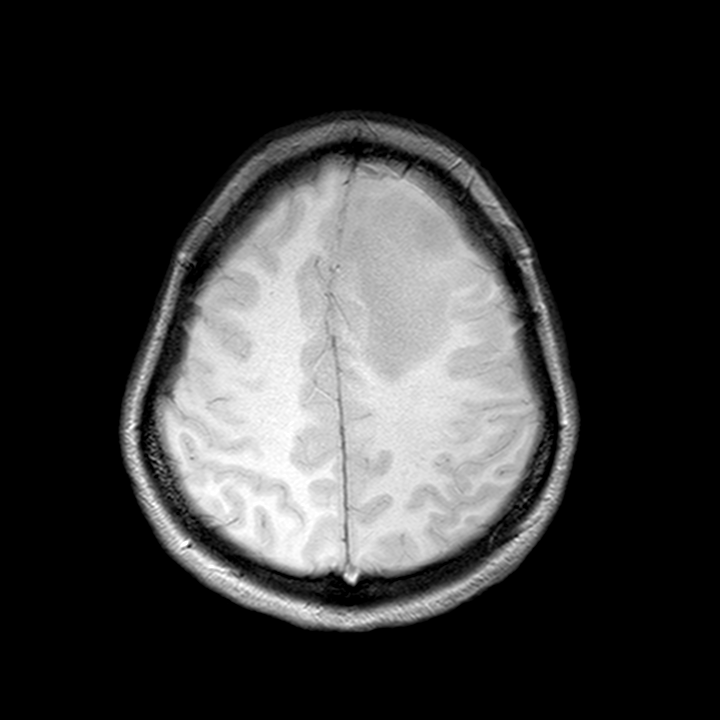

Supplement: Supplementary file 6 [file DataSheet6.zip › MRI-T1-FFE/T1-FFE-14.tif]

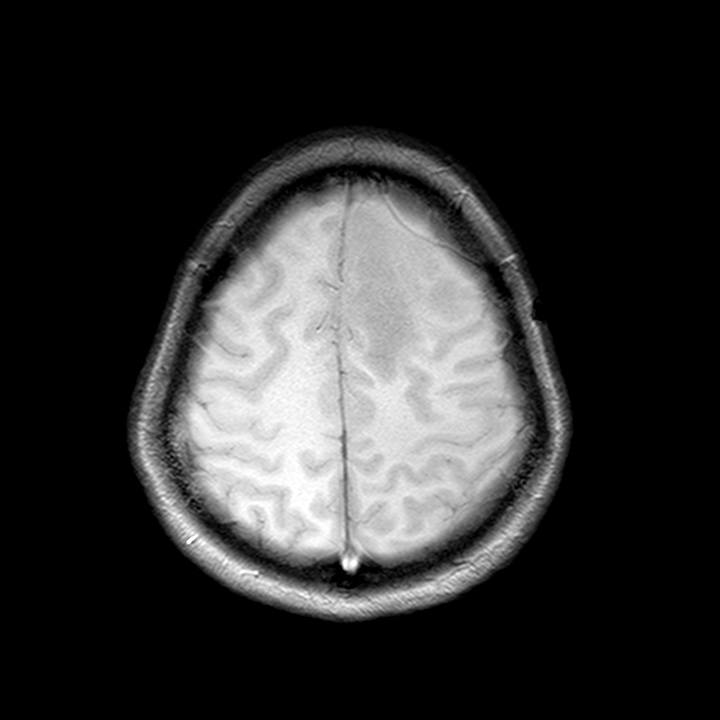

Supplement: Supplementary file 6 [file DataSheet6.zip › MRI-T1-FFE/T1-FFE-15.tif]

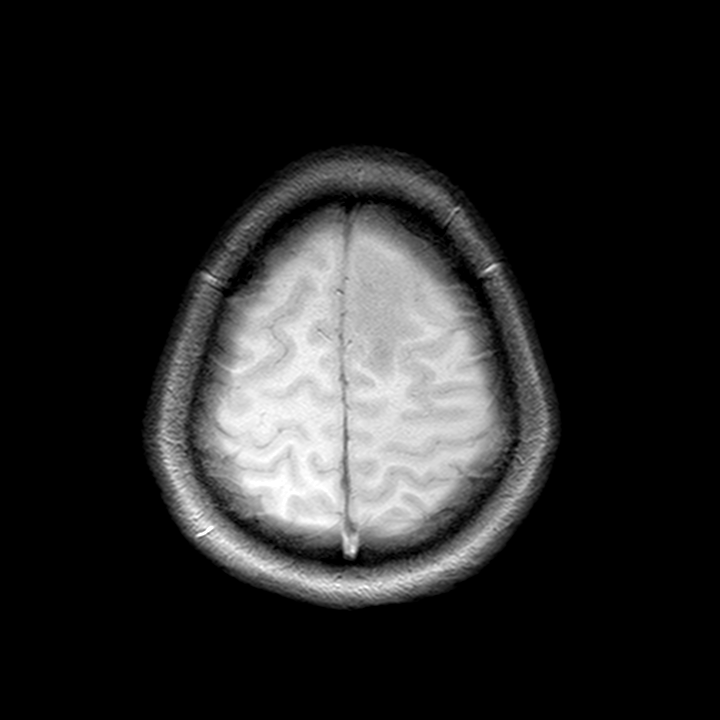

Supplement: Supplementary file 6 [file DataSheet6.zip › MRI-T1-FFE/T1-FFE-16.tif]

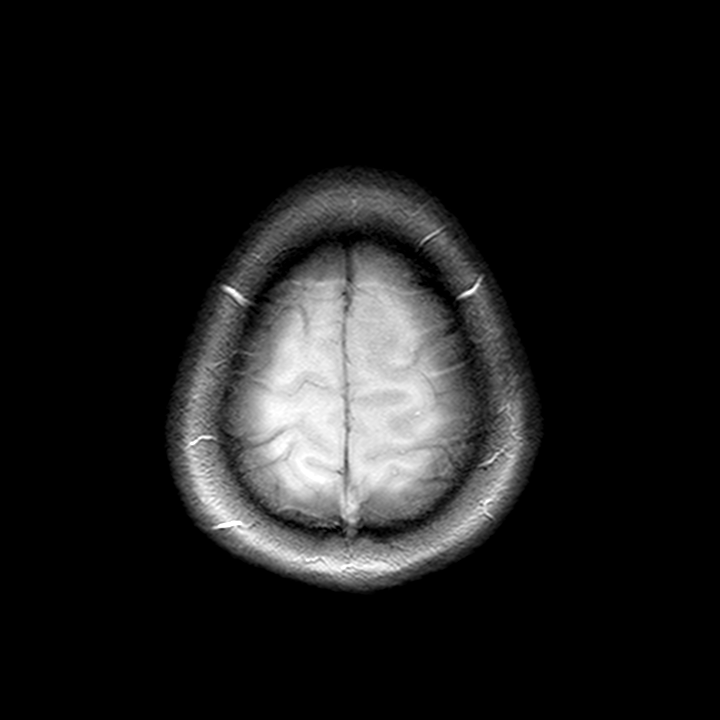

Supplement: Supplementary file 6 [file DataSheet6.zip › MRI-T1-FFE/T1-FFE-17.tif]

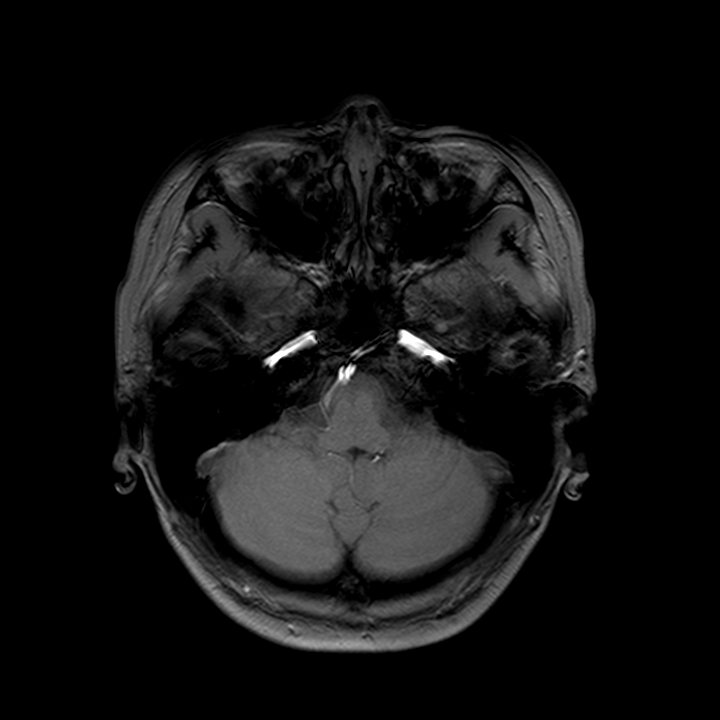

Supplement: Supplementary file 6 [file DataSheet6.zip › MRI-T1-FFE/T1-FFE-2.tif]

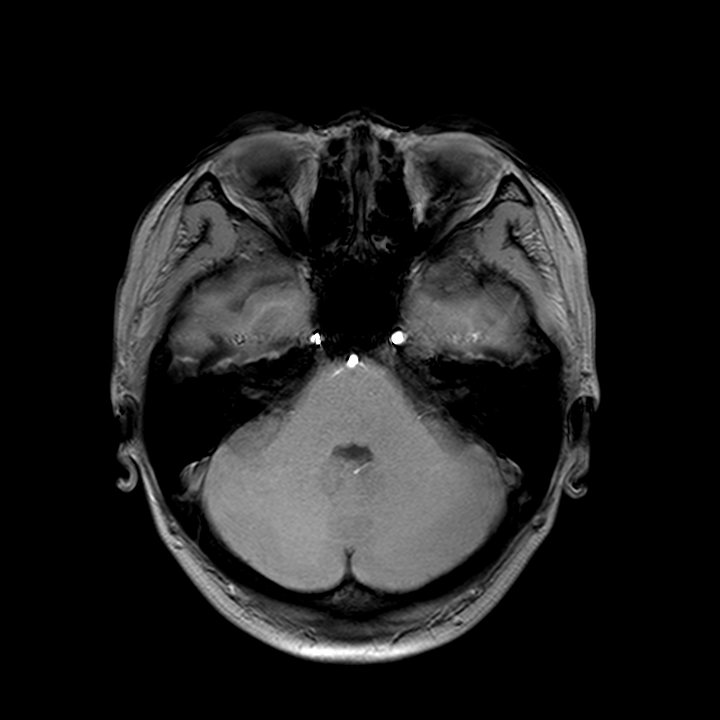

Supplement: Supplementary file 6 [file DataSheet6.zip › MRI-T1-FFE/T1-FFE-3.tif]

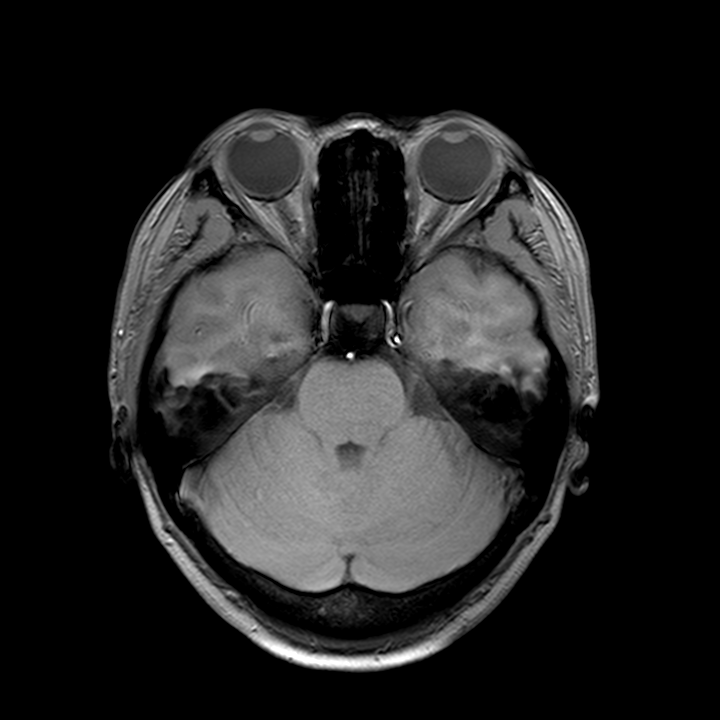

Supplement: Supplementary file 6 [file DataSheet6.zip › MRI-T1-FFE/T1-FFE-4.tif]

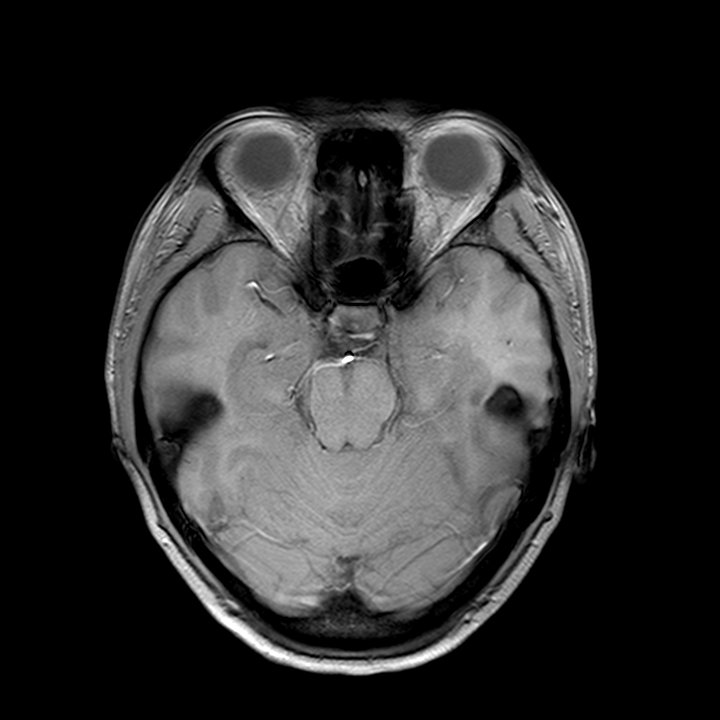

Supplement: Supplementary file 6 [file DataSheet6.zip › MRI-T1-FFE/T1-FFE-5.tif]

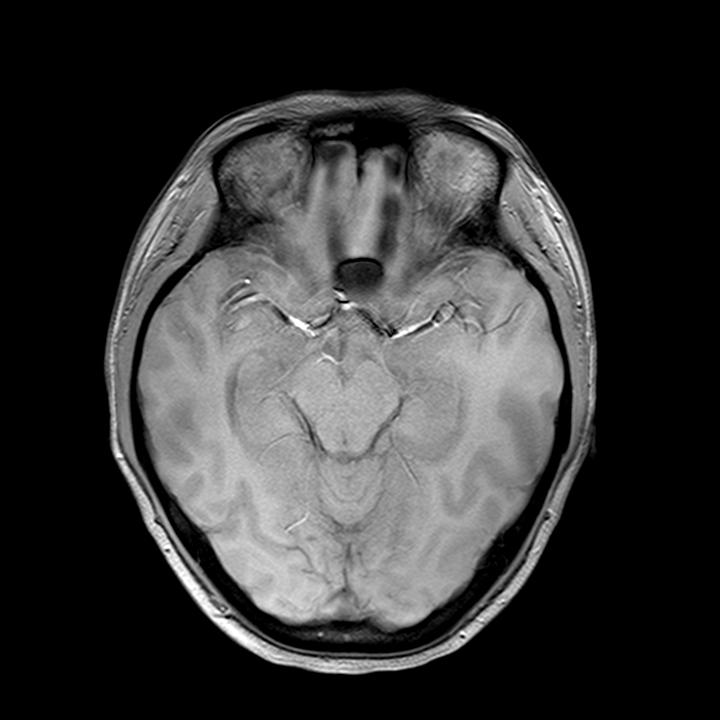

Supplement: Supplementary file 6 [file DataSheet6.zip › MRI-T1-FFE/T1-FFE-6.tif]

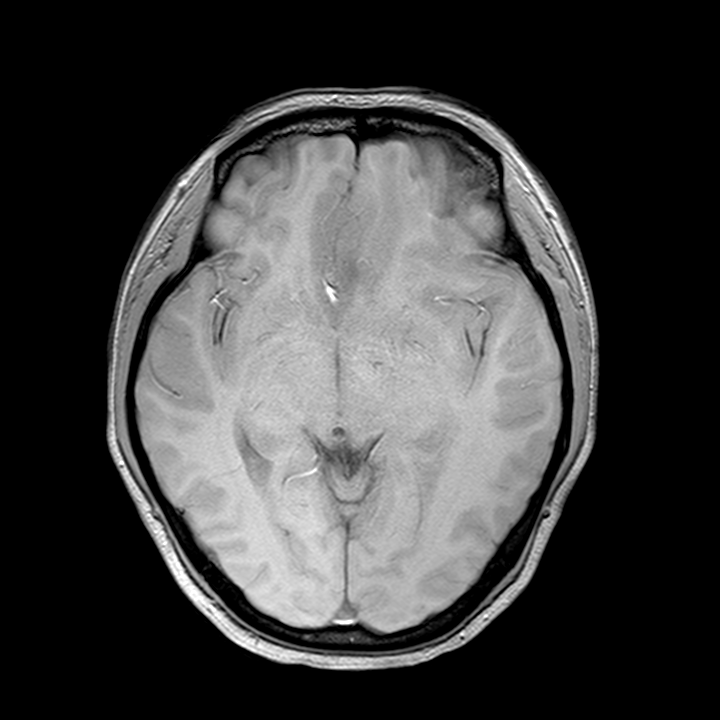

Supplement: Supplementary file 6 [file DataSheet6.zip › MRI-T1-FFE/T1-FFE-7.tif]

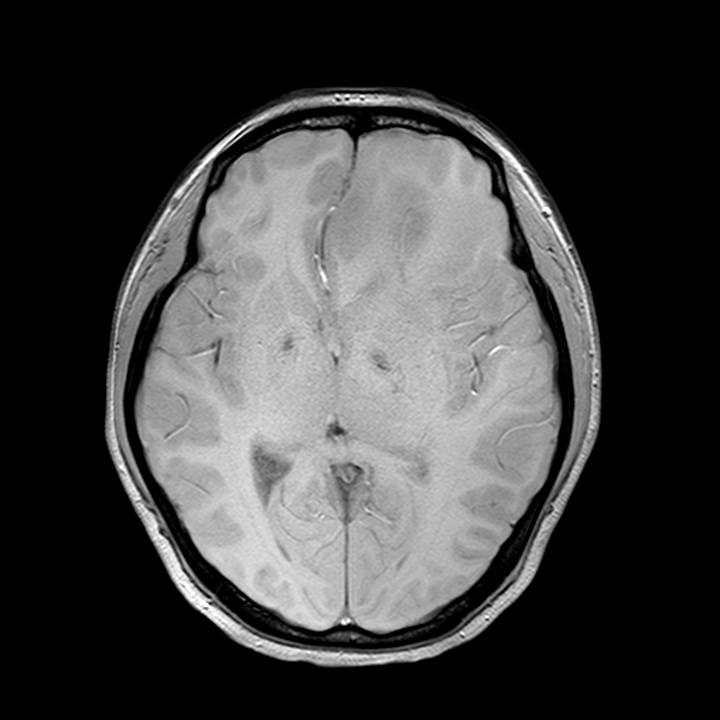

Supplement: Supplementary file 6 [file DataSheet6.zip › MRI-T1-FFE/T1-FFE-8.tif]

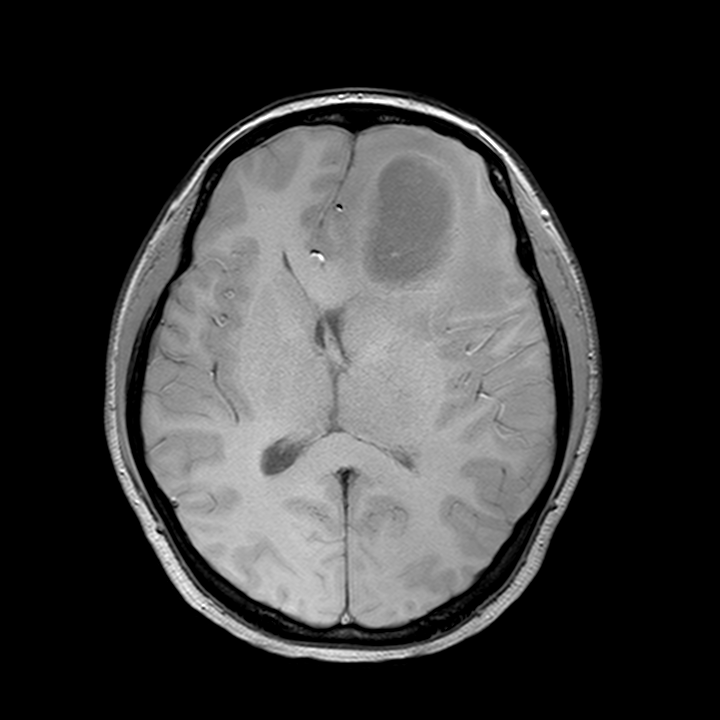

Supplement: Supplementary file 6 [file DataSheet6.zip › MRI-T1-FFE/T1-FFE-9.tif]

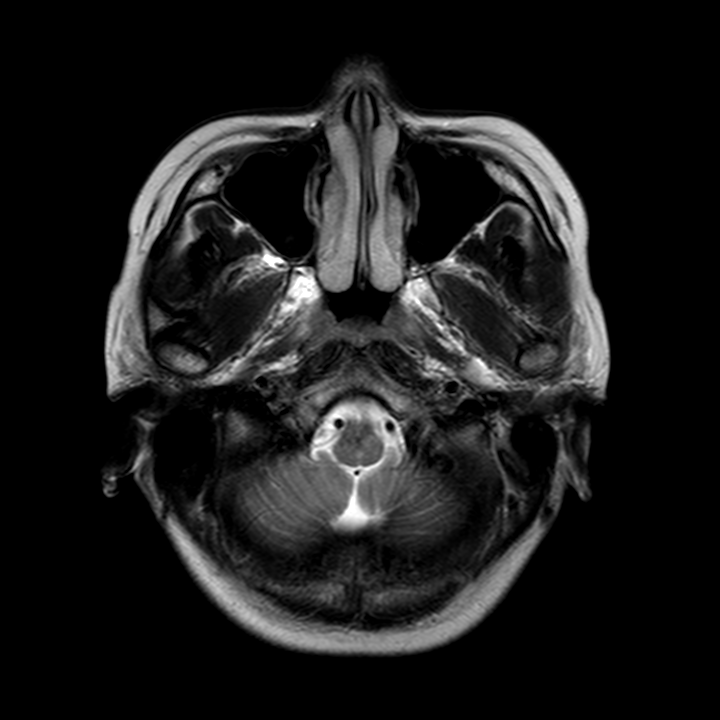

Supplement: Supplementary file 7 [file DataSheet7.zip › MRI-T2/T2-0.tif]

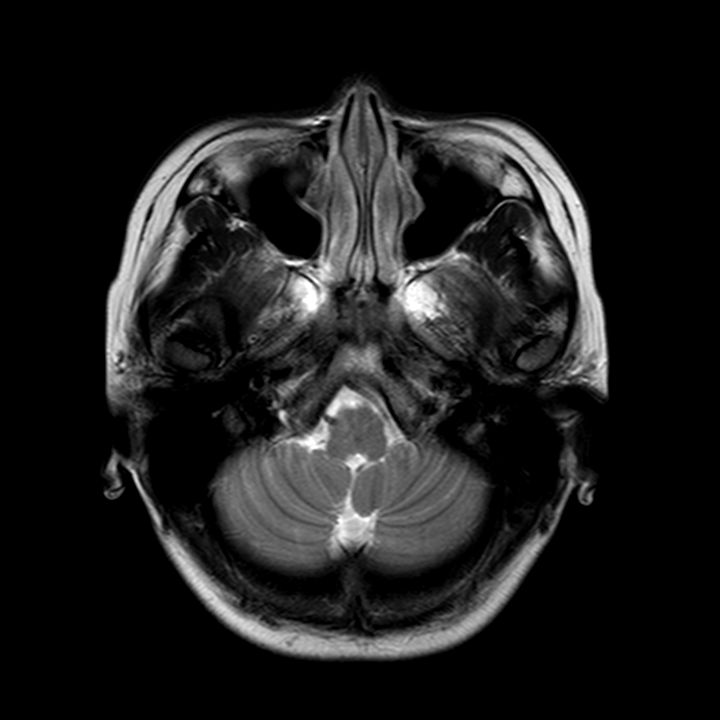

Supplement: Supplementary file 7 [file DataSheet7.zip › MRI-T2/T2-1.tif]

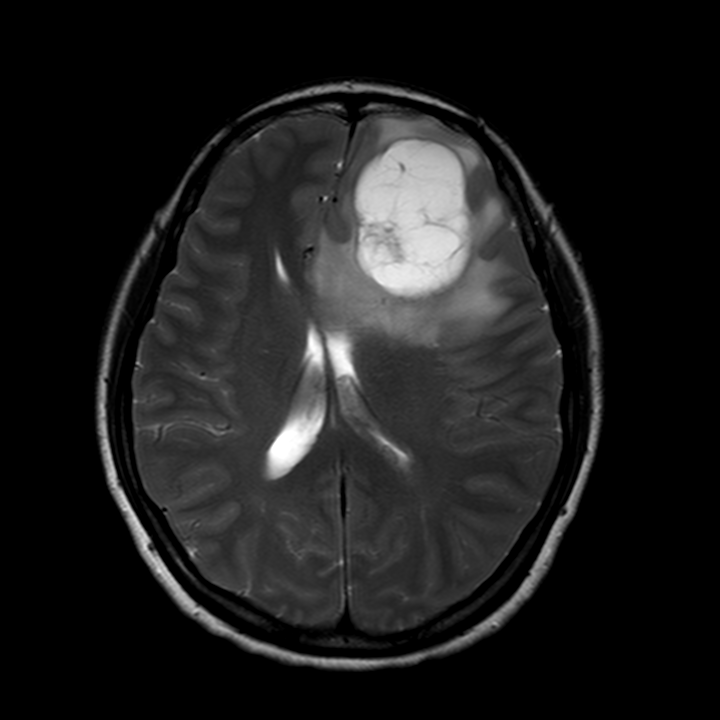

Supplement: Supplementary file 7 [file DataSheet7.zip › MRI-T2/T2-10.tif]

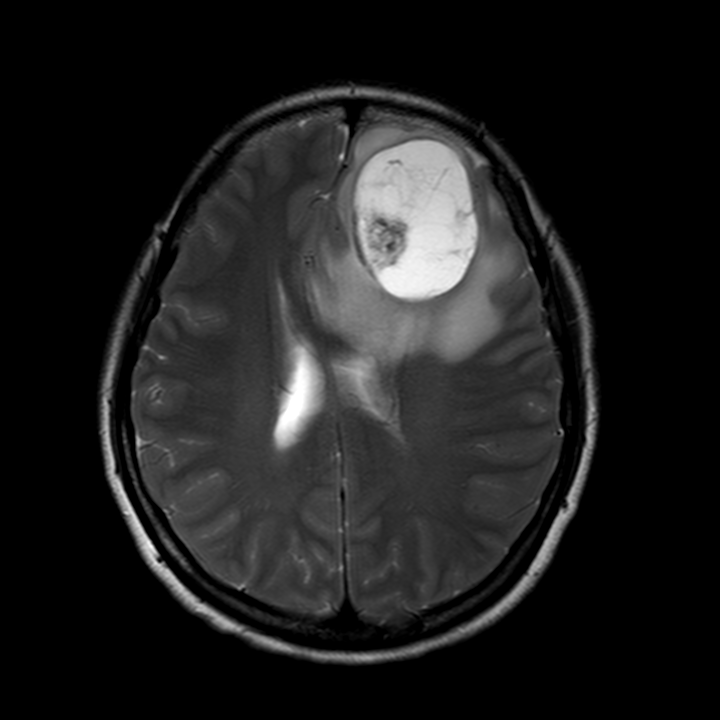

Supplement: Supplementary file 7 [file DataSheet7.zip › MRI-T2/T2-11.tif]

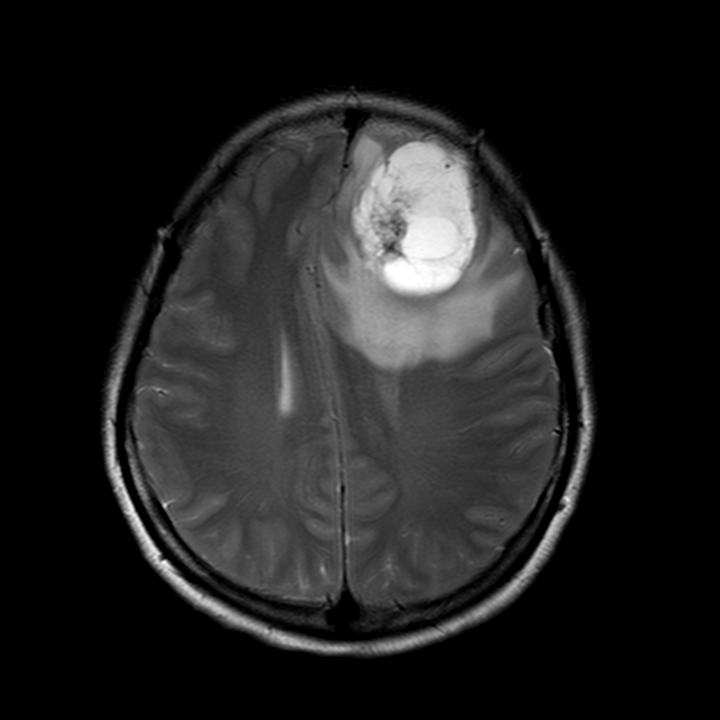

Supplement: Supplementary file 7 [file DataSheet7.zip › MRI-T2/T2-12.tif]

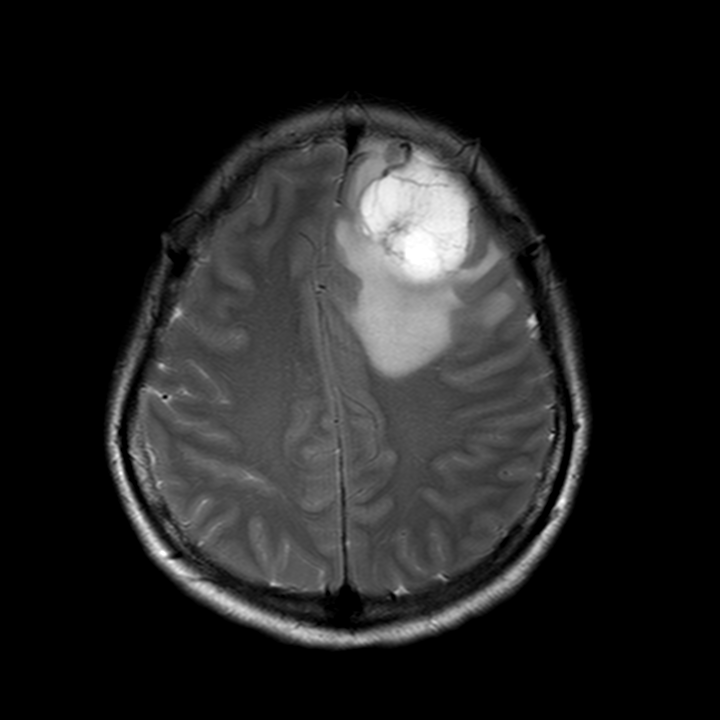

Supplement: Supplementary file 7 [file DataSheet7.zip › MRI-T2/T2-13.tif]

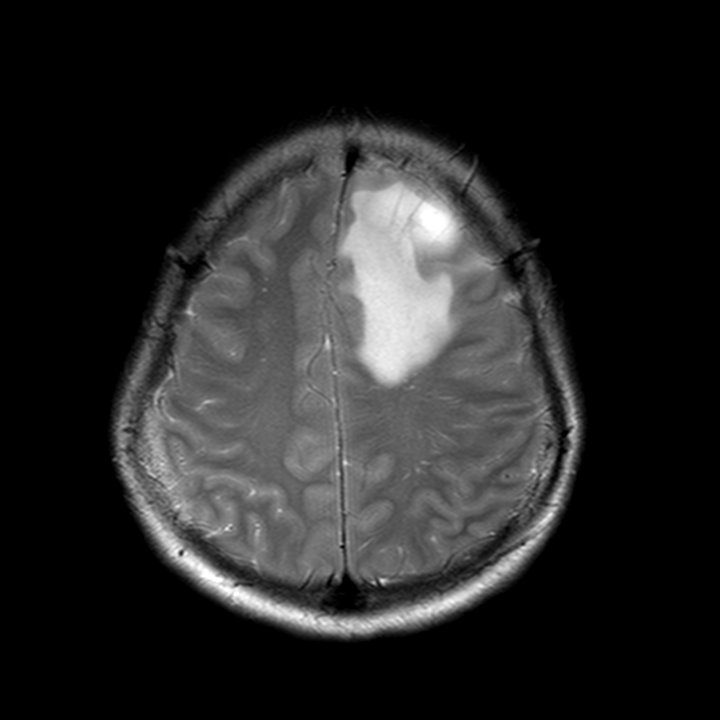

Supplement: Supplementary file 7 [file DataSheet7.zip › MRI-T2/T2-14.tif]

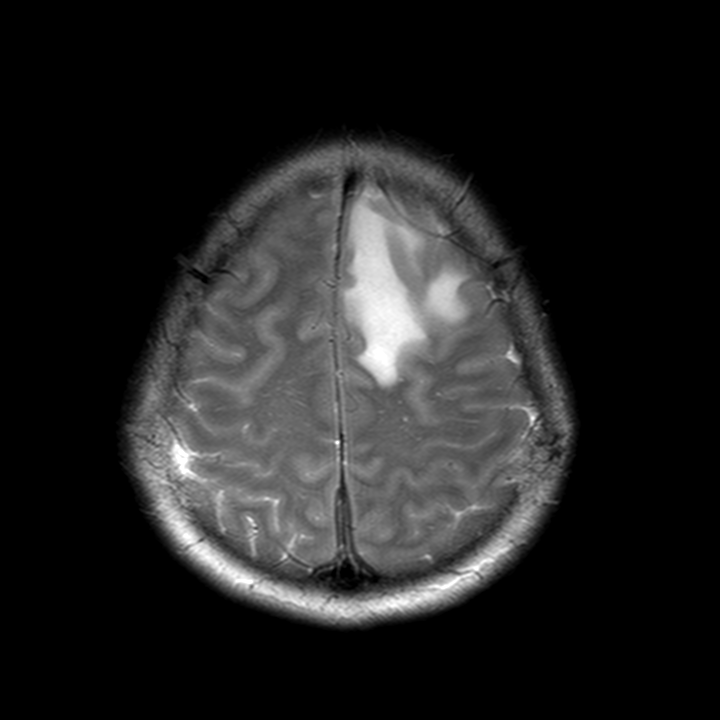

Supplement: Supplementary file 7 [file DataSheet7.zip › MRI-T2/T2-15.tif]

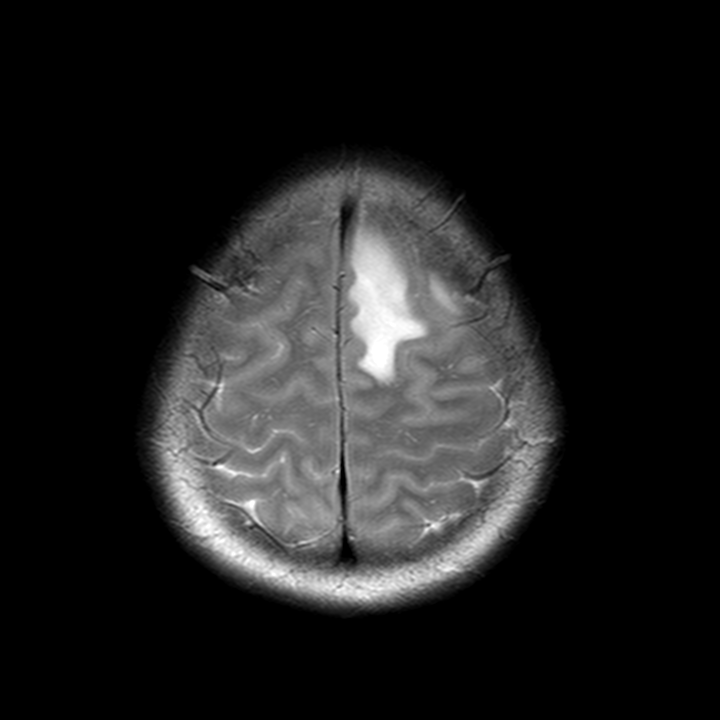

Supplement: Supplementary file 7 [file DataSheet7.zip › MRI-T2/T2-16.tif]

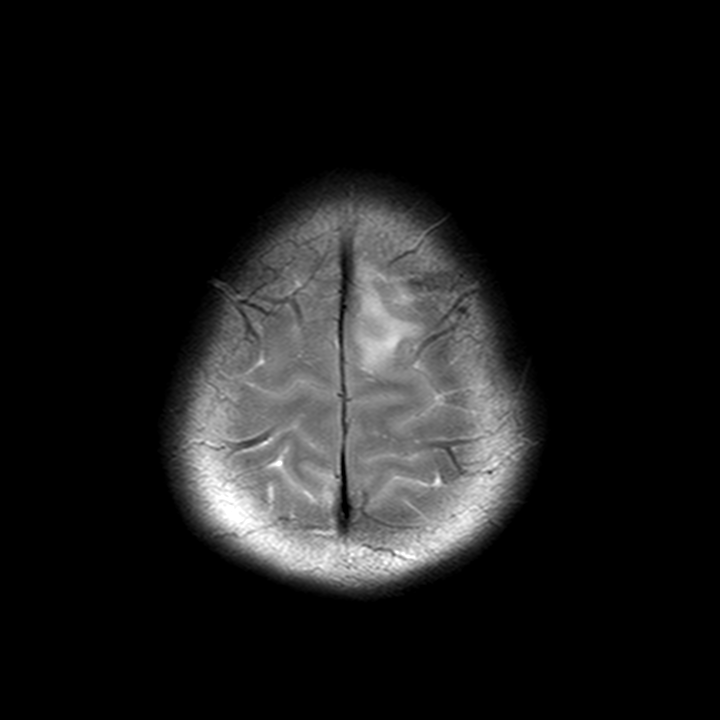

Supplement: Supplementary file 7 [file DataSheet7.zip › MRI-T2/T2-17.tif]

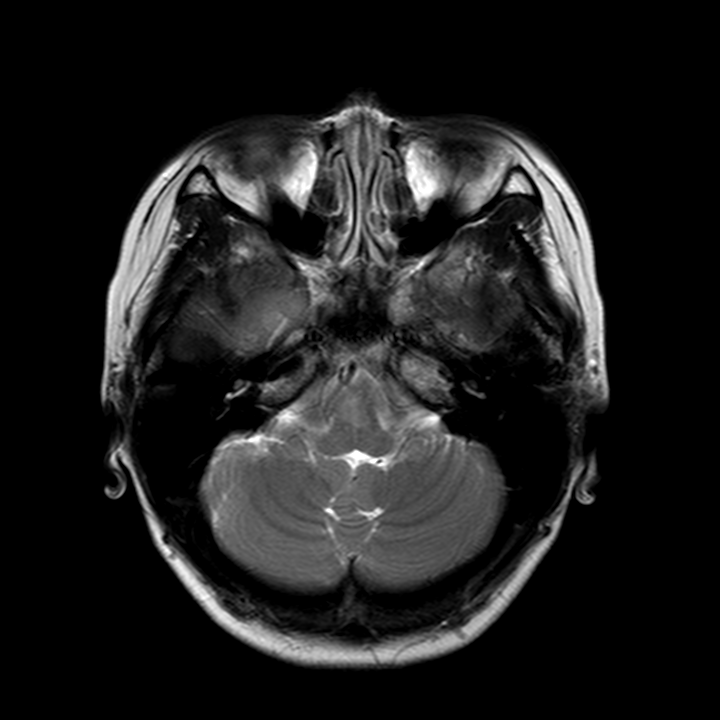

Supplement: Supplementary file 7 [file DataSheet7.zip › MRI-T2/T2-2.tif]

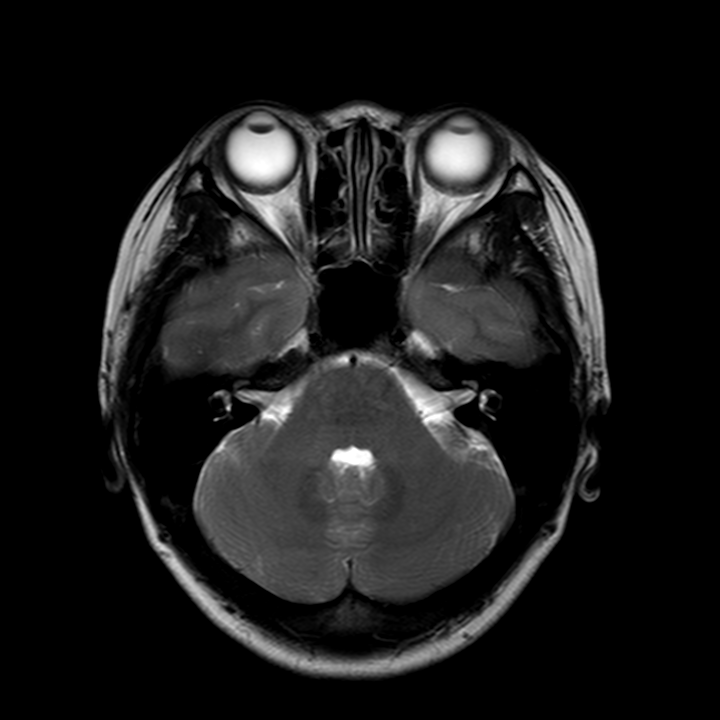

Supplement: Supplementary file 7 [file DataSheet7.zip › MRI-T2/T2-3.tif]

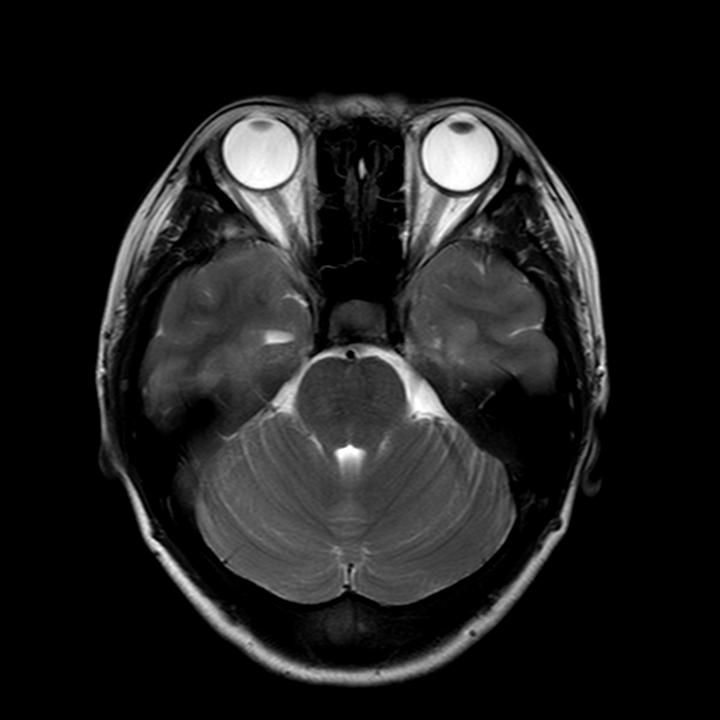

Supplement: Supplementary file 7 [file DataSheet7.zip › MRI-T2/T2-4.tif]

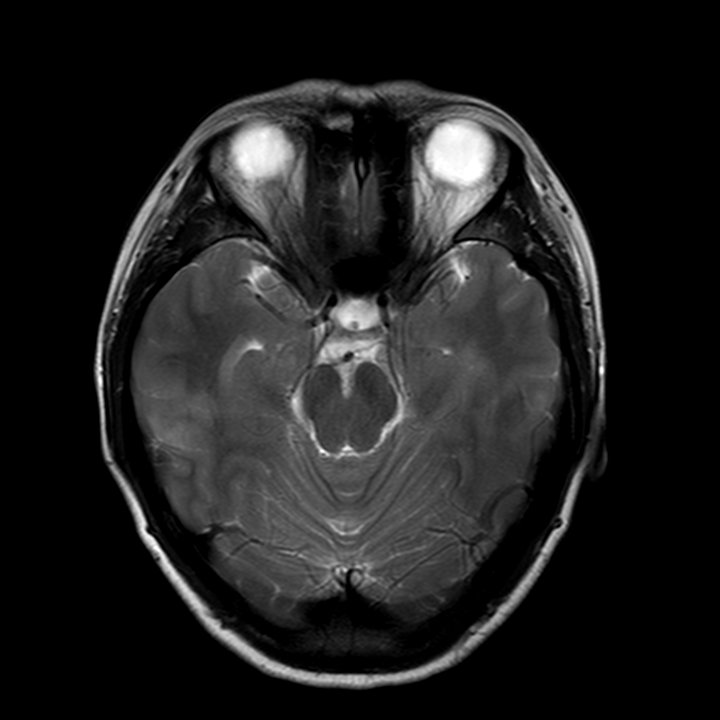

Supplement: Supplementary file 7 [file DataSheet7.zip › MRI-T2/T2-5.tif]

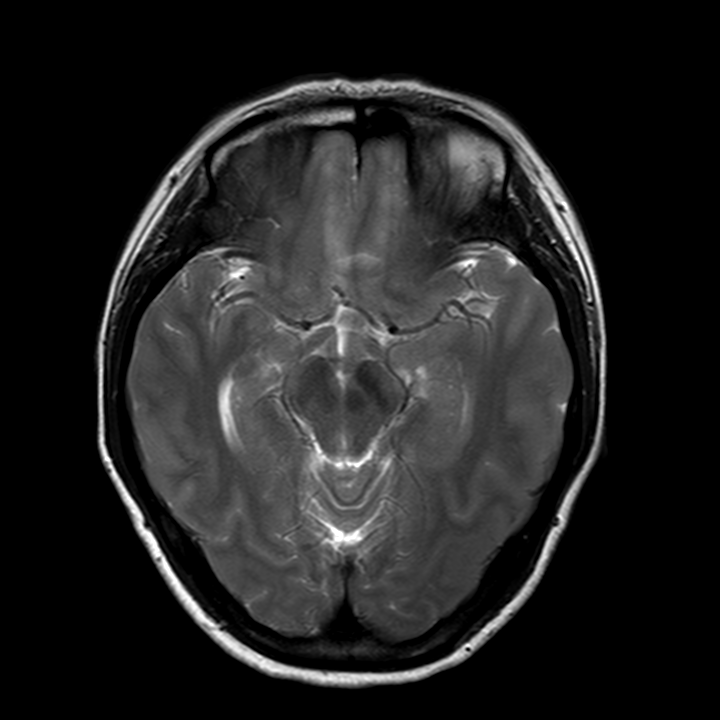

Supplement: Supplementary file 7 [file DataSheet7.zip › MRI-T2/T2-6.tif]

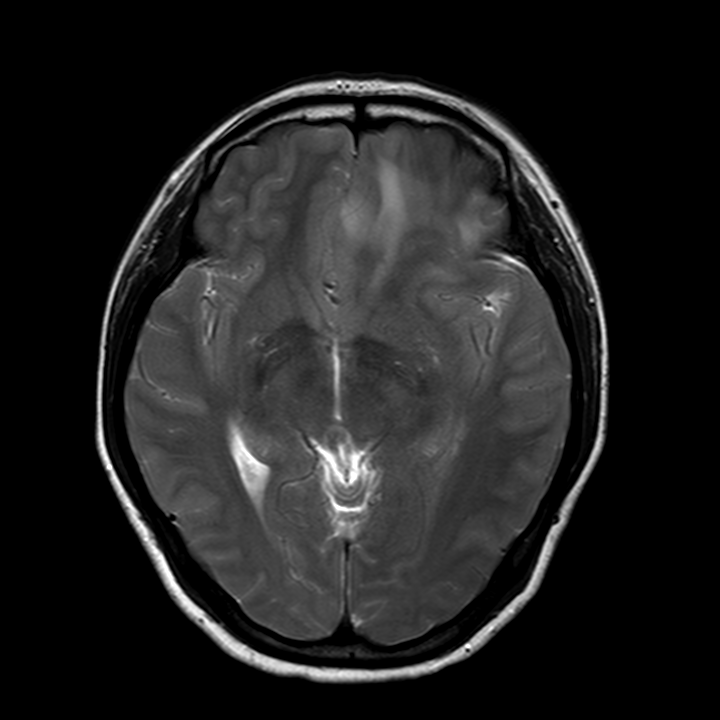

Supplement: Supplementary file 7 [file DataSheet7.zip › MRI-T2/T2-7.tif]

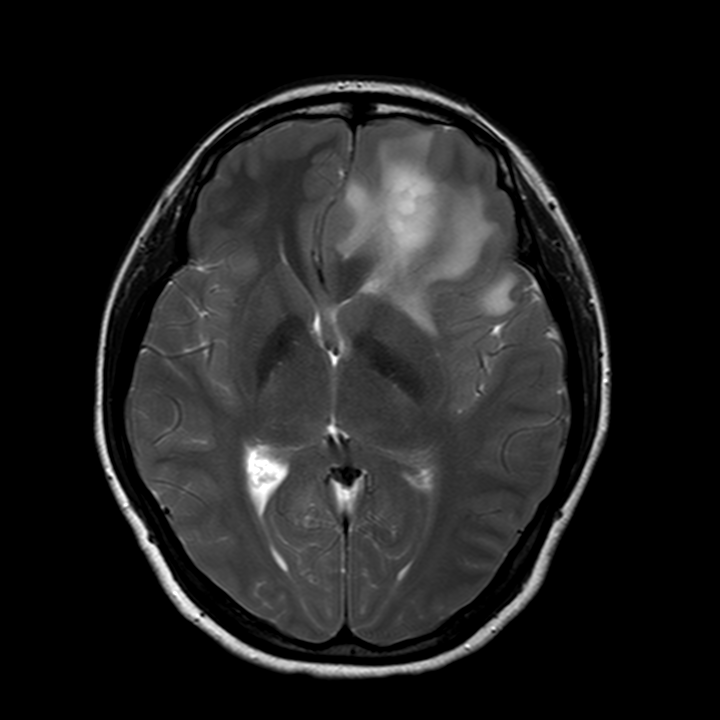

Supplement: Supplementary file 7 [file DataSheet7.zip › MRI-T2/T2-8.tif]

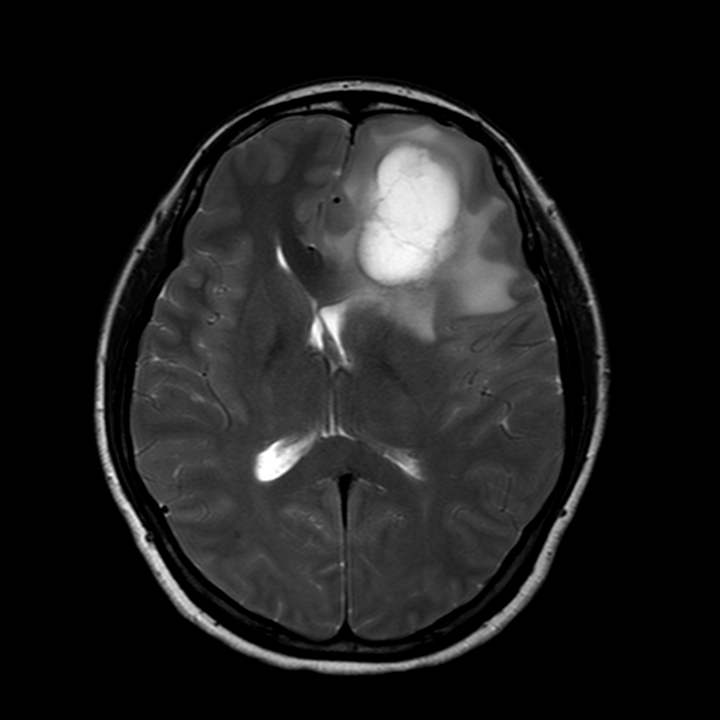

Supplement: Supplementary file 7 [file DataSheet7.zip › MRI-T2/T2-9.tif]

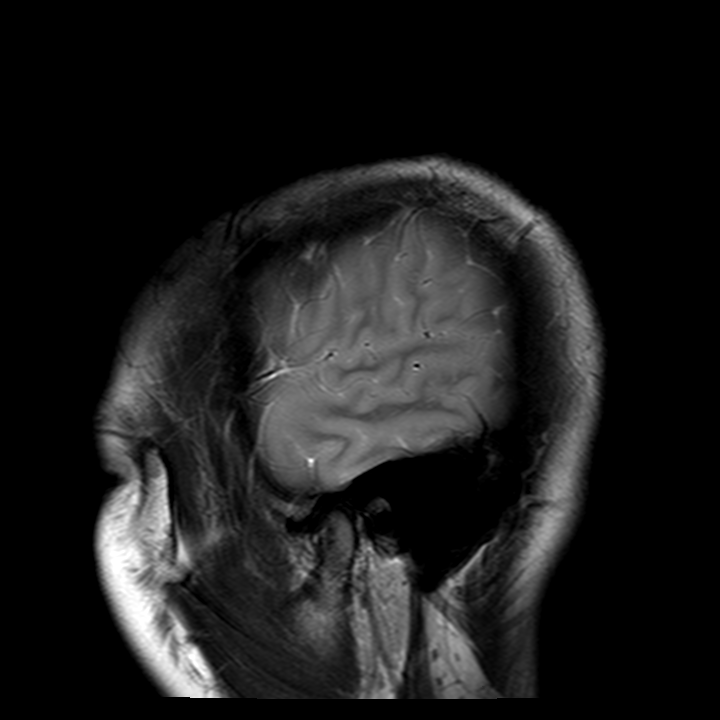

Supplement: Supplementary file 7 [file DataSheet7.zip › MRI-T2/T2-sagittal0.tif]

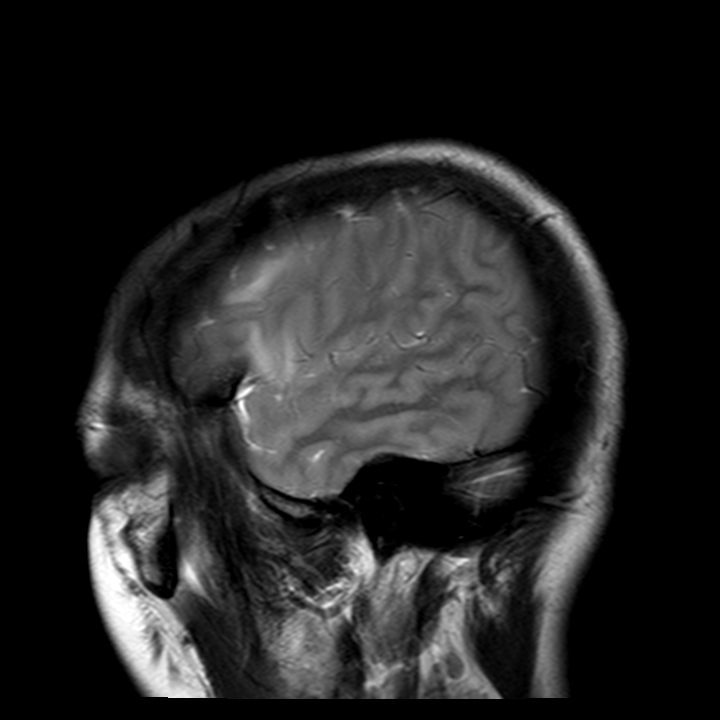

Supplement: Supplementary file 7 [file DataSheet7.zip › MRI-T2/T2-sagittal1.tif]

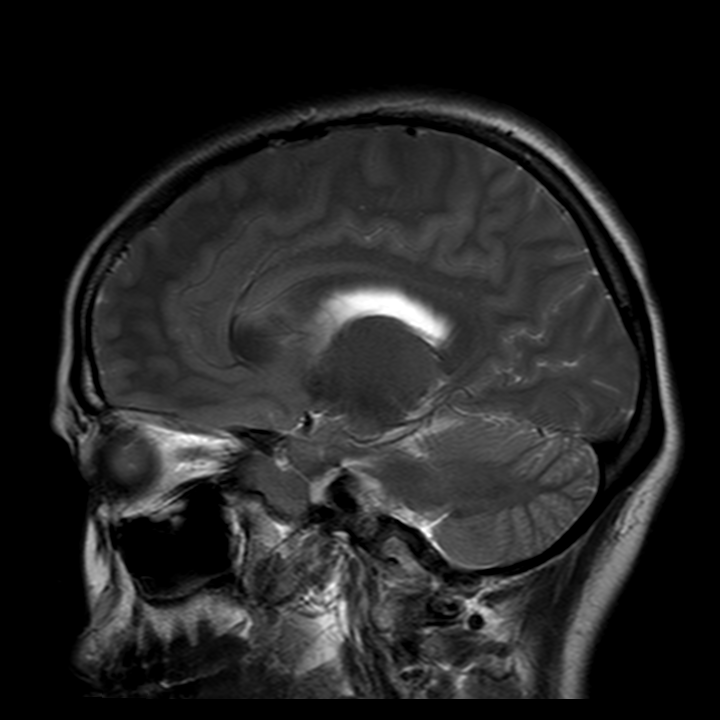

Supplement: Supplementary file 7 [file DataSheet7.zip › MRI-T2/T2-sagittal10.tif]

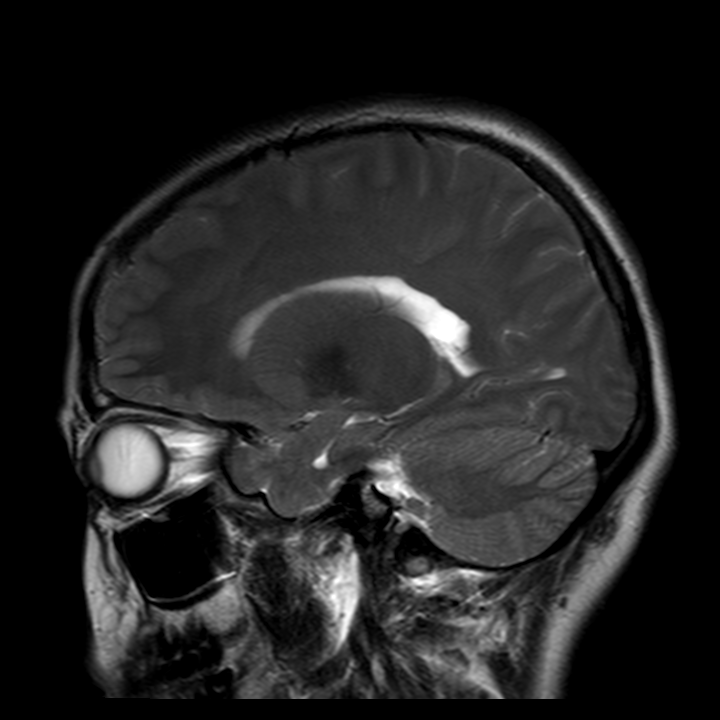

Supplement: Supplementary file 7 [file DataSheet7.zip › MRI-T2/T2-sagittal11.tif]

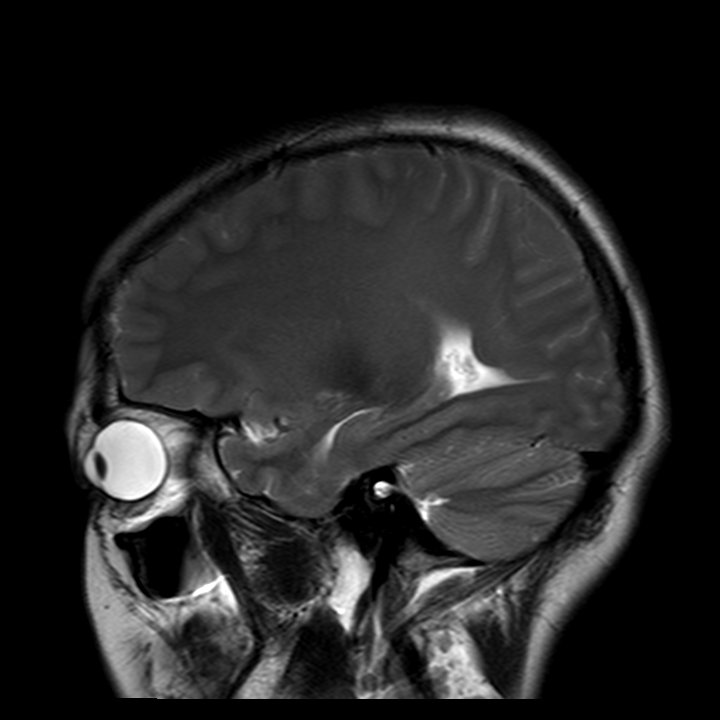

Supplement: Supplementary file 7 [file DataSheet7.zip › MRI-T2/T2-sagittal12.tif]

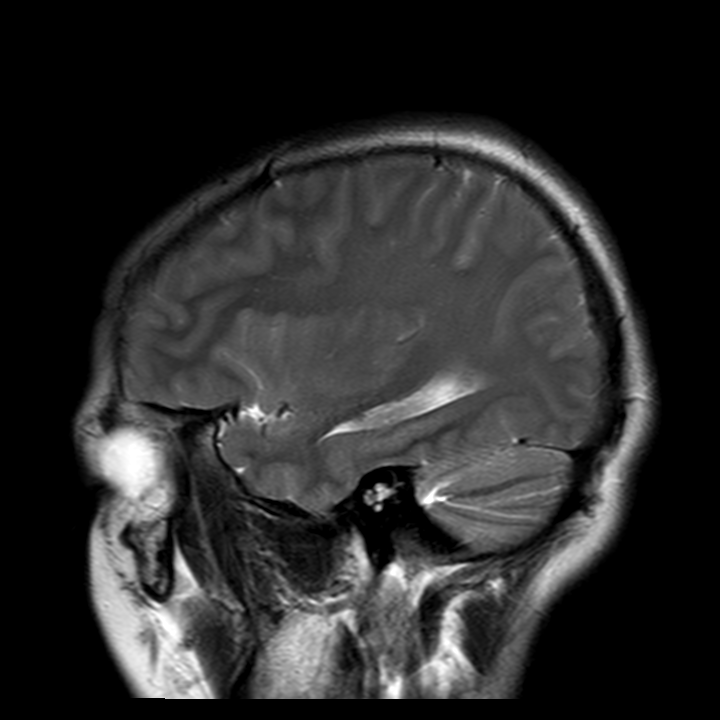

Supplement: Supplementary file 7 [file DataSheet7.zip › MRI-T2/T2-sagittal13.tif]

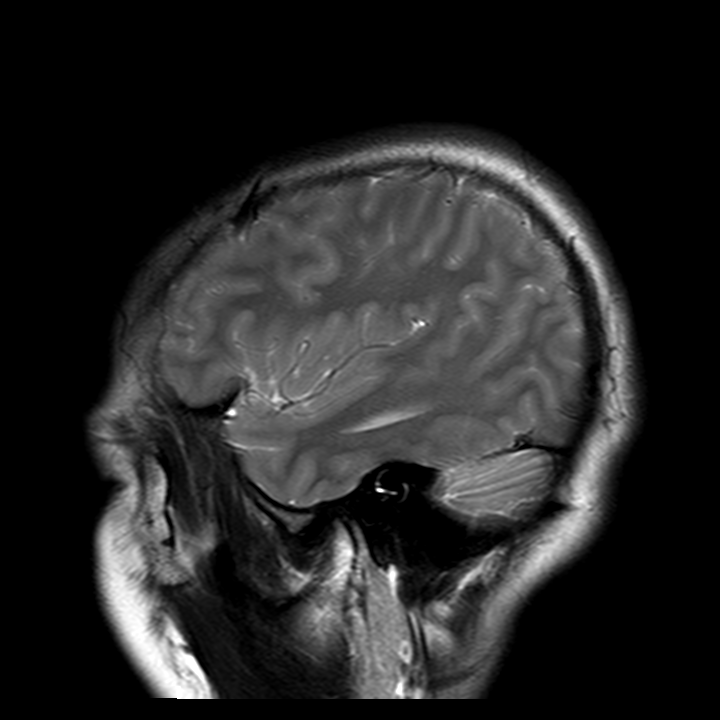

Supplement: Supplementary file 7 [file DataSheet7.zip › MRI-T2/T2-sagittal14.tif]

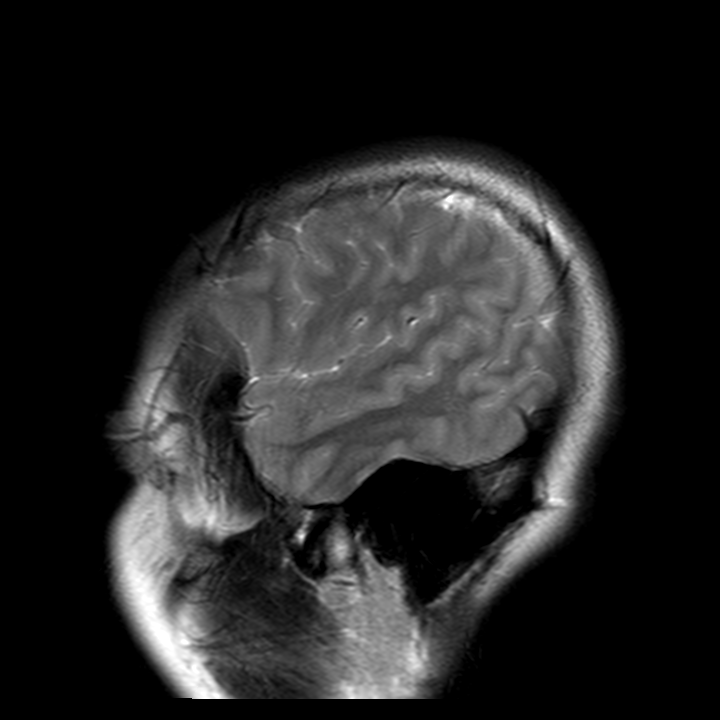

Supplement: Supplementary file 7 [file DataSheet7.zip › MRI-T2/T2-sagittal15.tif]

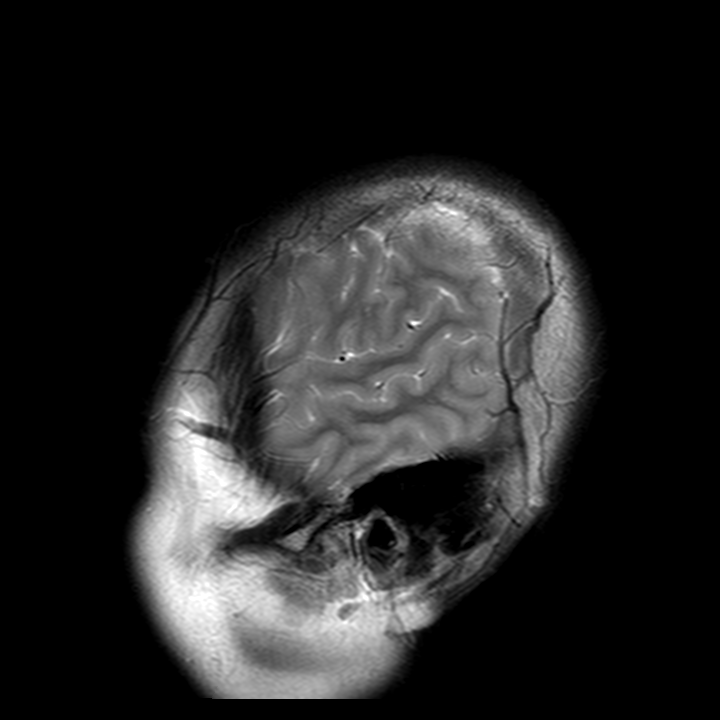

Supplement: Supplementary file 7 [file DataSheet7.zip › MRI-T2/T2-sagittal16.tif]

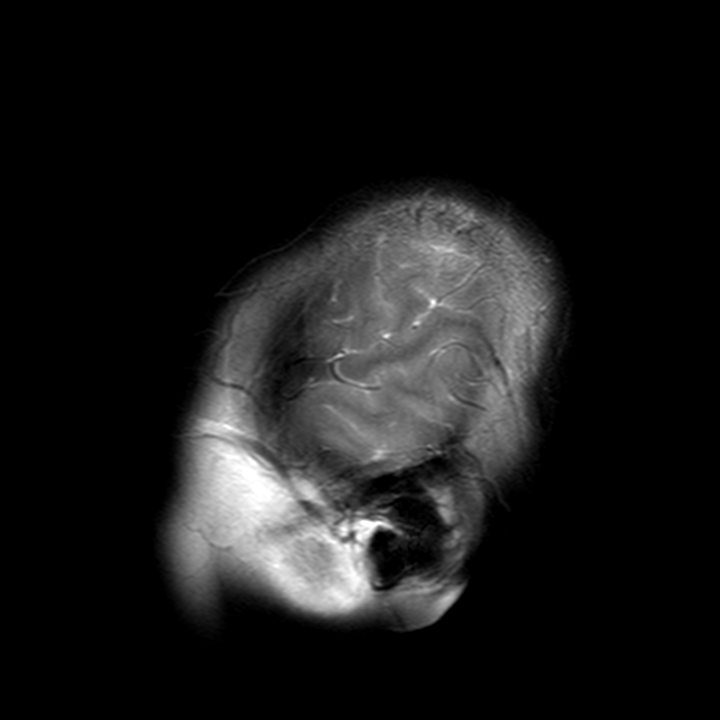

Supplement: Supplementary file 7 [file DataSheet7.zip › MRI-T2/T2-sagittal17.tif]

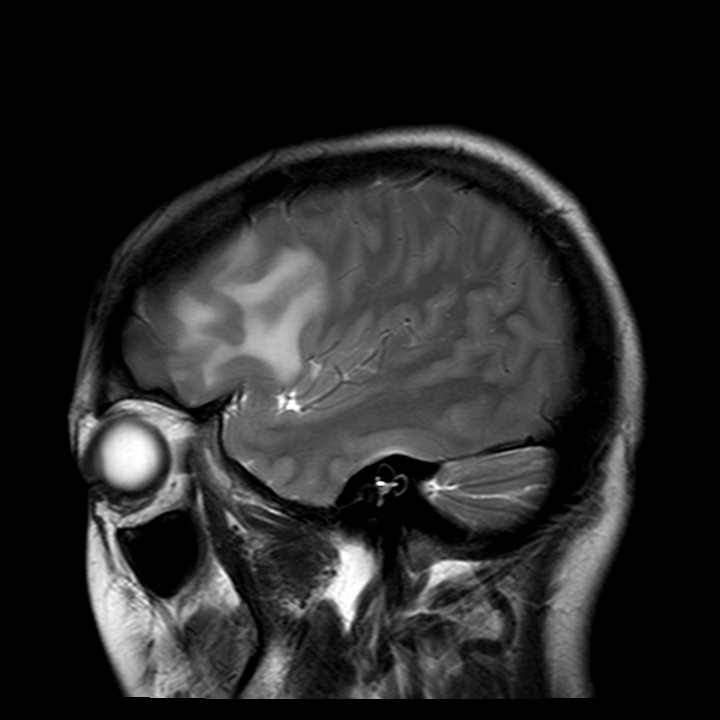

Supplement: Supplementary file 7 [file DataSheet7.zip › MRI-T2/T2-sagittal2.tif]

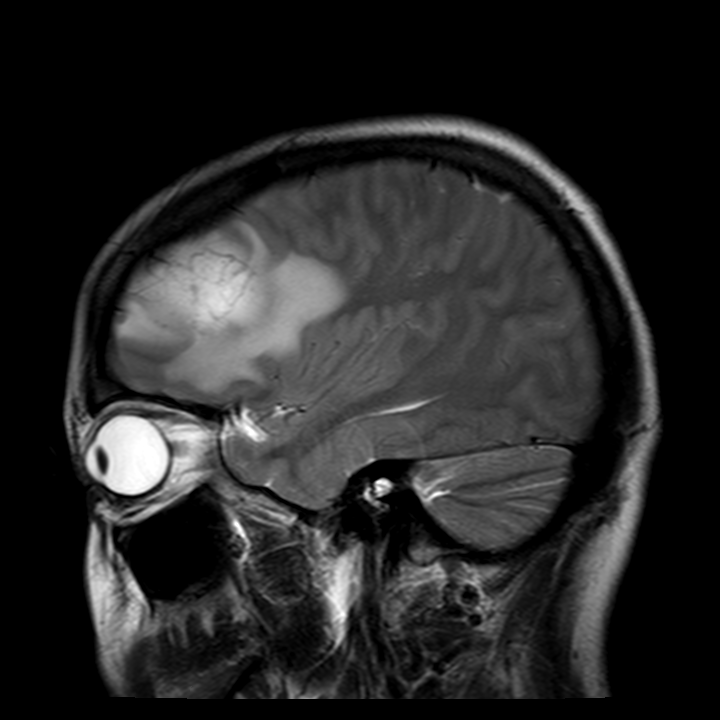

Supplement: Supplementary file 7 [file DataSheet7.zip › MRI-T2/T2-sagittal3.tif]

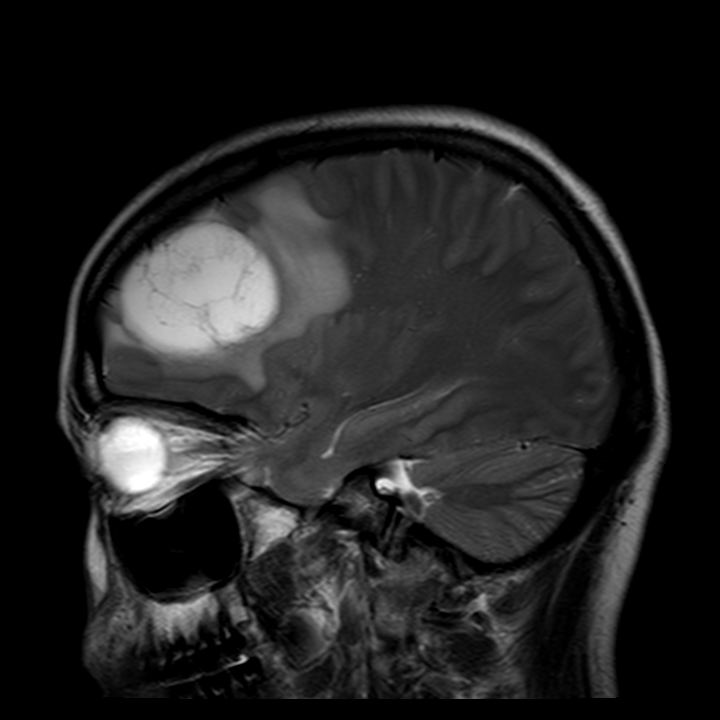

Supplement: Supplementary file 7 [file DataSheet7.zip › MRI-T2/T2-sagittal4.tif]

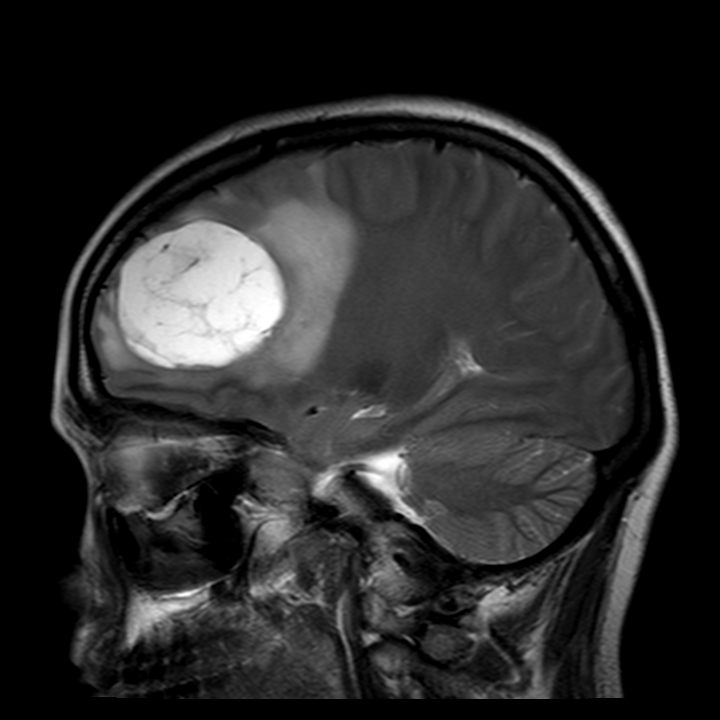

Supplement: Supplementary file 7 [file DataSheet7.zip › MRI-T2/T2-sagittal5.tif]

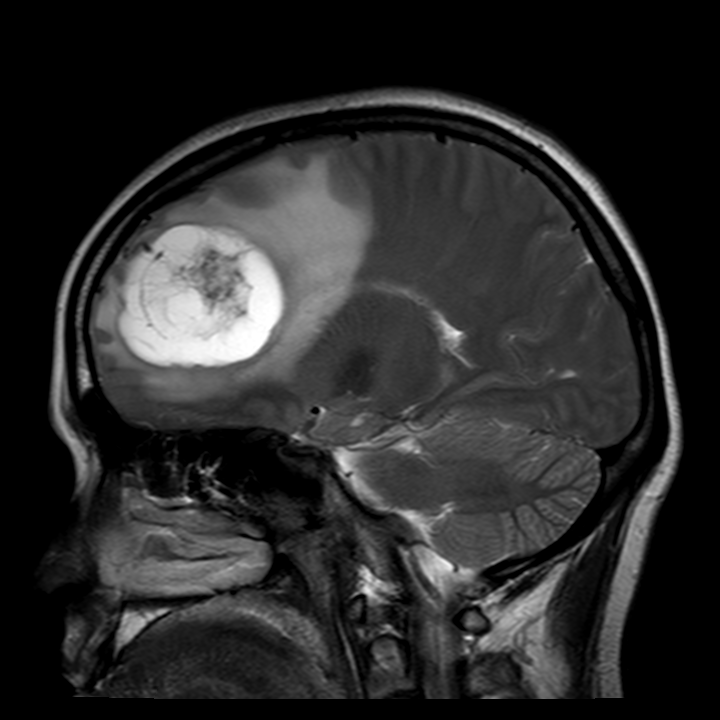

Supplement: Supplementary file 7 [file DataSheet7.zip › MRI-T2/T2-sagittal6.tif]

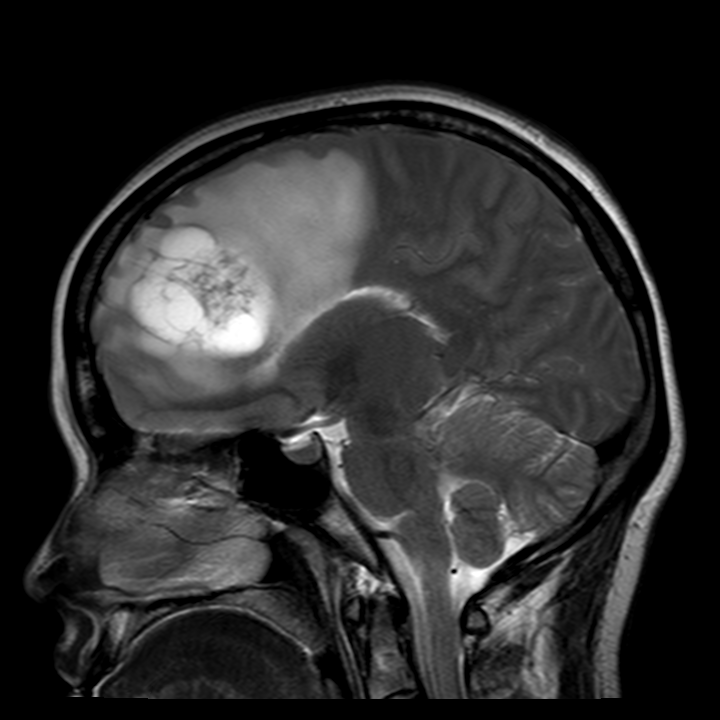

Supplement: Supplementary file 7 [file DataSheet7.zip › MRI-T2/T2-sagittal7.tif]

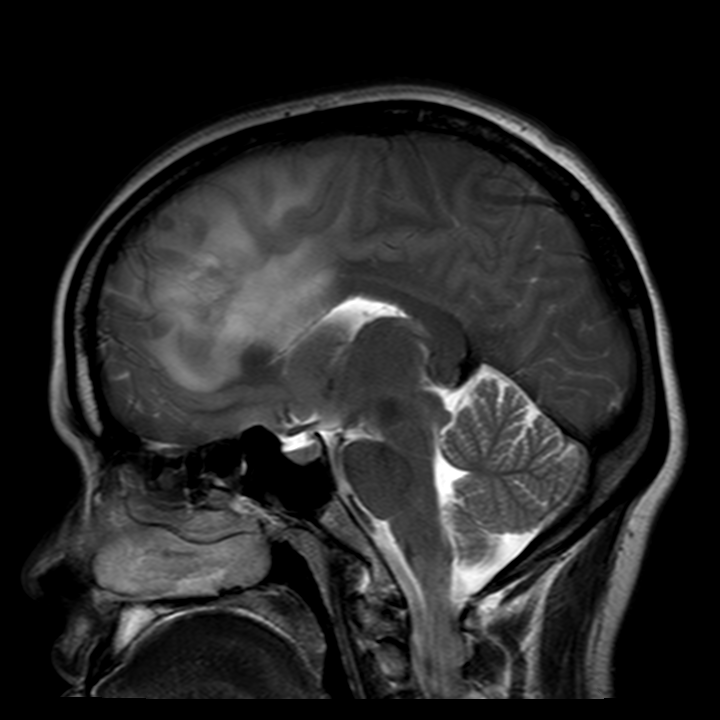

Supplement: Supplementary file 7 [file DataSheet7.zip › MRI-T2/T2-sagittal8.tif]

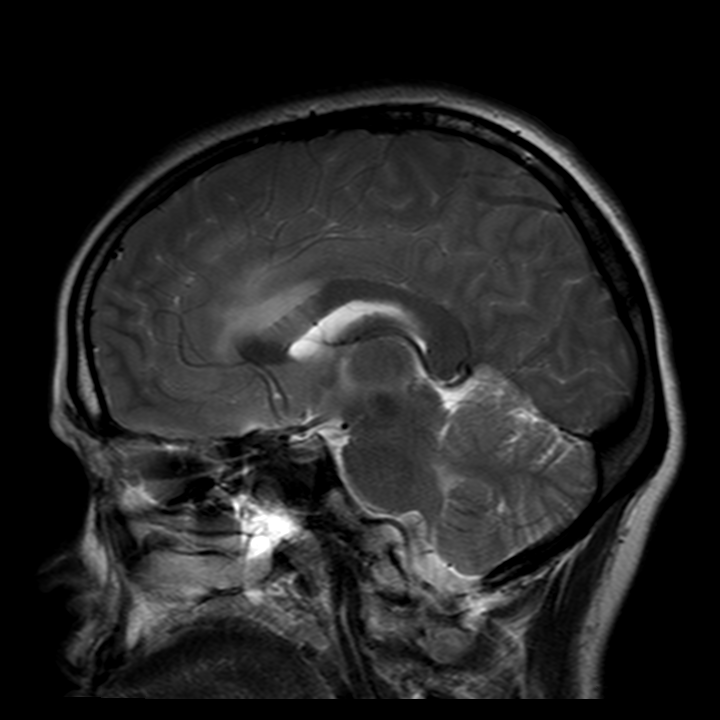

Supplement: Supplementary file 7 [file DataSheet7.zip › MRI-T2/T2-sagittal9.tif]
